# PIWI proximity proteome reveals Set1-mediated piRNA biogenesis for transposon silencing at telomeres

**DOI:** 10.1038/s44319-026-00901-6

**Published:** 2026-08-26

**Authors:** Wakana Isshiki, Hiroko Kozuka-Hata, Masaaki Oyama, Toshie Kai, Taichiro Iki

**Affiliations:** 1 Graduate School of Frontier Biosciences, The University of Osaka, Osaka, Japan; 2 Medical Proteomics Laboratory, The Institute of Medical Science, The University of Tokyo, Tokyo, Japan; 3 Graduate School of Biostudies, Kyoto University, Kyoto, Japan

**Keywords:** Chromatin, Transcription & Genomics, RNA Biology

## Abstract

Silencing complexes formed by PIWI-clade Argonaute (Ago) proteins and PIWI-interacting RNAs (piRNAs) are essential guardians of genome integrity, restricting the activities of transposable elements (TEs) in the animal germline. However, our understanding of PIWI-piRNA-directed TE silencing remains incomplete. Here, we systemically characterize the proximity proteome of the PIWI members Piwi, Aubergine (Aub), and Ago3 in the germline of Drosophila ovaries. Functional screening identifies previously uncharacterized factors involved in TE silencing, including the H3K4me3 writer and transcriptional coactivator Set1. Transcriptome analysis reveals that Set1 acts as an indispensable repressor of TEs, particularly of those at telomeres. Set1 is required for the production of antisense, TE-targeting piRNAs. Genome-wide chromatin profiling by CUT&Tag demonstrates that Set1 preferentially associates with TE sequences, including their 3’UTRs, and is localized at subtelomeric piRNA-producing loci. It also controls the accumulation of Rhino, a key activator of piRNA precursor transcription, at these sites. Notably, the catalytic activity of Set1 is dispensable. Our findings uncover a noncanonical function of Set1 in Piwi-mediated TE silencing in germline nuclei.

## Synopsis

PIWI proteins and piRNAs protect the germline from transposable elements. Proximity proteomes of PIWI members in the Drosophila germline identify the histone methyltransferase Set1 that plays a noncanonical role in the production of piRNAs and silencing of transposons, particularly those at telomeres.

- Proximity proteomes of the three Drosophila PIWI proteins (Piwi, Aub, and Ago3) identify previously uncharacterized factors involved in germline transposon silencing.
- Set1 plays a noncanonical role in the Piwi pathway. It promotes piRNA biogenesis and silencing of transposons, particularly those at telomeres.
- Transposable element 3’UTRs can serve as cis-elements for Set1-dependent piRNA biogenesis.

## Abstract (web summary)

PIWI proteins and piRNAs protect the germline from transposable elements. Proximity proteomes of PIWI members in the Drosophila germline identify the histone methyltransferase Set1 that plays a noncanonical role in the production of piRNAs and silencing of transposons, particularly those at telomeres.

## Graphical abstract

**Figure:**
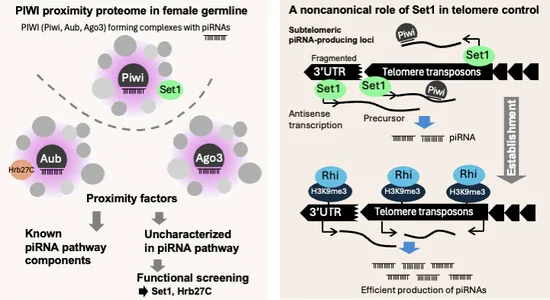

## Introduction

Transposable elements (TEs) are mobile DNA sequences constituting a substantial part of genomes in most organisms (Wells and Feschotte, 2020). Although TEs act as drivers of genomic evolution and species diversification, the harmful potential as mutagens requires the hosts to establish control mechanisms. The control is particularly important in germline cells where activated TEs accumulate damage on the inheritable genomes and impair the production of functional gametes. In animals, TE control in germline cells relies on silencing mediated by complexes of PIWI clade Argonaute (Ago) family proteins and the sequence-specific guide, PIWI-interacting (pi)RNAs (Wang et al, 2023b).

PIWI-piRNA-directed TE silencing has been studied in different animals, including the invertebrate model, *Drosophila melanogaster. D. melanogaster* possesses three PIWI members, including Piwi, Aubergine (Aub), and Ago3, all of which are expressed in germline cells. Piwi differs from the other two in that it functions in the nucleus. Guided by piRNAs, Piwi complexes recognize the nascent transcripts from target loci, mediating the (co-)transcriptional silencing and heterochromatin formation (Mugat et al, 2020; Le Thomas et al, 2013; Sienski et al, 2012; Wu et al, 2025). In contrast, Aub and Ago3 are cytoplasmic proteins, localizing to nuage, a perinuclear membraneless organelle formed in germline cells (Kawaguchi et al, 2025; Lim and Kai, 2015). In nuage, Aub and Ago3 mediate the reciprocal cleavage of targets containing sense and antisense TE sequences. 3’ cleavage products generated by either of the two proteins are passed to the other (i.e., from Aub to Ago3, or from Ago3 to Aub), then processed into a piRNA. Newly generated piRNAs direct the next round of target cleavage and piRNA production. These cycles are called ping-pong, coupling TE degradation and piRNA amplification in germline cells. Because target cleavage occurs between the nucleotides complementary to the 10th and 11th nucleotides of guide piRNA, and new piRNA is produced from the 5’ end of the cleavage product, germline piRNAs exhibit a 10-nt overlap signature (ping-pong signature). In addition to ping-pong, precursor fragments incorporated into Aub can be processed into multiple piRNAs on the mitochondrial outer membrane. These phasing/trailer piRNAs are mainly loaded onto Piwi, translocating the complexes to the nucleus (Ge et al, 2019; Yashiro et al, 2018). Ping-pong cycles process diverse transcripts, including intact TE mRNAs and long non-coding RNAs derived by noncanonical transcription of large intergenic regions, archiving truncated TE sequences, defined as piRNA cluster loci (Brennecke et al, 2007).

piRNA-directed TE silencing is achieved through interactions between PIWI proteins and other components. These include nuclear factors involved in Piwi-piRNA-directed co-transcriptional silencing and heterochromatin formation (e.g., Panx/Silencio, Gtsf1/Arx, Nxf2, SetDB1/Eggless) (Fabry et al, 2019; Batki et al, 2019; Yu et al, 2015; Sienski et al, 2015; Murano et al, 2019; Ohtani et al, 2013; Zhao et al, 2019). In cytoplasm, Tudor domain proteins (Krimper, Tejas, Tapas, Qin/Kumo) and RNA helicases (Spn-E, Vas) are enriched in nuage and support the ping-pong cycles mediated by Aub and Ago3 (Lin et al, 2023; Handler et al, 2011; Zhang et al, 2011; Anand and Kai, 2012; Lim and Kai, 2007; Patil and Kai, 2010; Sato et al, 2015; Webster et al, 2015). Nonetheless, characterizing the interactions between PIWI members and other factors is often challenging because of their dynamics in piRNA biogenesis and TE silencing. Hence, the repertoire of piRNA pathway components is heretofore unelucidated, and our understanding of piRNA-directed TE silencing remains incomplete.

Proximity-dependent biotin labeling (BioID/TurboID) is a powerful tool for characterizing the physical interactions and proximity relationships of proteins in vivo (Roux et al, 2012; Branon et al, 2018; Choi-Rhee et al, 2004). These techniques rely on the promiscuous biotin ligase activity exhibited by BirA derived from *Escherichia coli*. TurboID utilizes an efficient and compact BirA variant called mini(m)Turbo established through directed evolution (Branon et al, 2018), which we previously introduced in male germline cells to study testis-specific factors (Iki et al, 2023; Kai et al, 2025; Iki et al, 2020). In order to deepen the understanding of piRNA-directed TE silencing, this study characterized the proteins in the physical proximity of individual PIWI proteins in female germline cells, where TE silencing has been extensively studied. The obtained proximity proteome of germline PIWI members not only confirmed reported interactions but also revealed the unrecognized links. Moreover, the PIWI proximity proteome contained a list of factors which have not been characterized in the piRNA pathway thus far. Of those, this study further characterizes Set1, a histone methyltransferase and transcriptional coactivator conserved across eukaryotes. Our data highlight the hitherto unappreciated importance of Set1 in Piwi-piRNA-directed TE silencing in telomeres of germline cells.

## Results

### Biotin labeling and characterization of germline PIWI proximity factors

**Figure 1:**
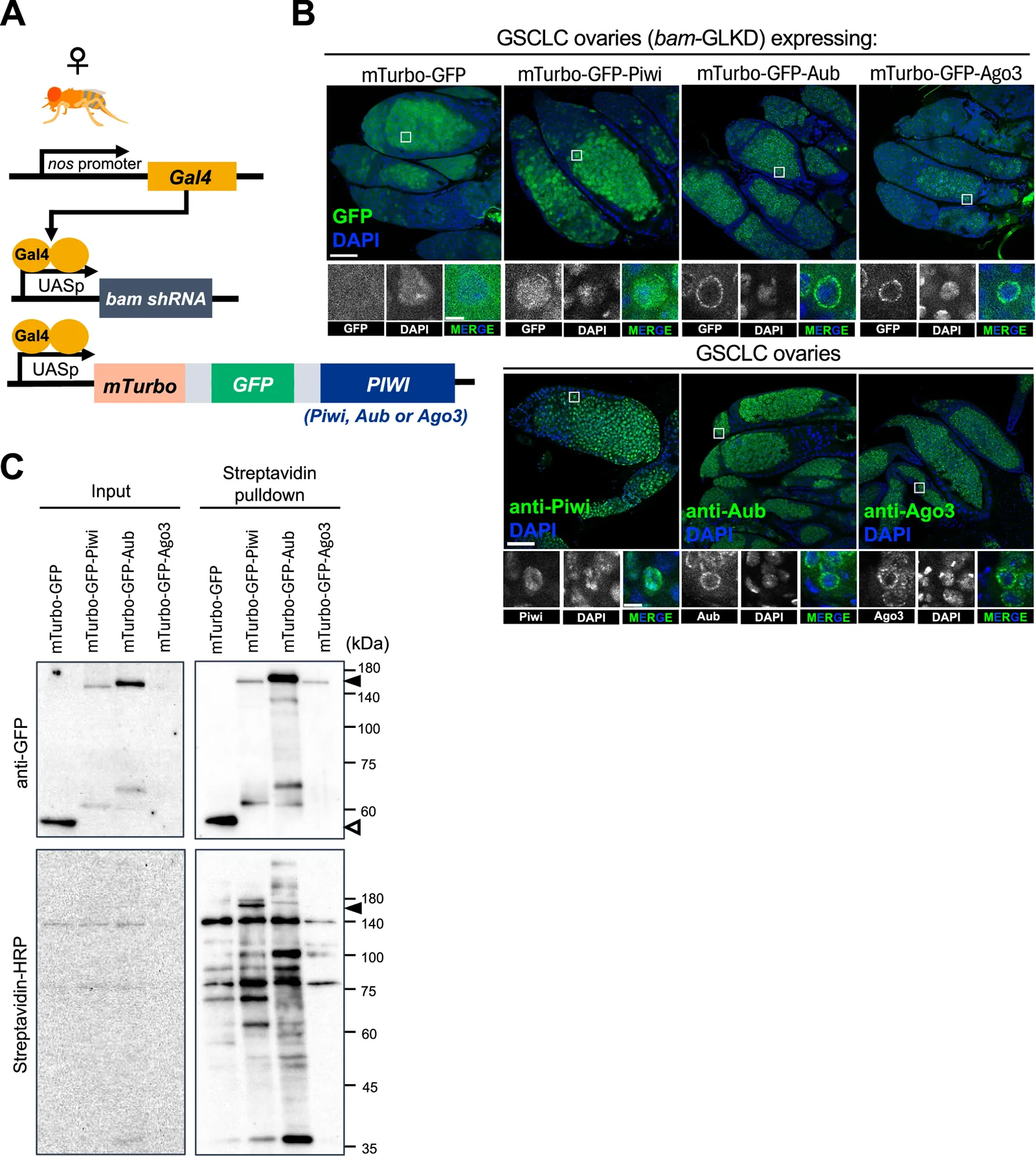
PIWI proximity biotin labeling in germline stem cell-like cells (GSCLCs). (**A**) Experimental design for PIWI proximity biotin labeling in female germline cells. mTurbo-GFP-Piwi, -Aub, or -Ago3 is expressed in germline cells using UASp/Gal4 system with NGT40; nos-Gal4 (NN) driver. mTurbo-GFP serves as a negative control. Depletion of *bam* using short hairpin (sh)RNA enables TurboID in GSCLCs. (**B**) Subcellular localization of mTurbo-GFP fusion proteins in GSCLC ovary. Upper panels: fluorescent signals from GFP (green). Lower panels: immunostaining signals from endogenous Piwi, Aub, or Ago3 (green). DAPI (blue) for nuclei. Scale bar  = 50 μm or 5 μm in the insets. (**C**) Blotting images for proteins extracted from GSCLC ovaries expressing mTurbo fusion proteins (Input), and the streptavidin-bound fraction (Pulldown). White and black arrowheads indicate mTurbo-GFP and mTurbo-GFP-PIWI proteins, respectively. Source data are available online for this figure.

**Figure EV1:**
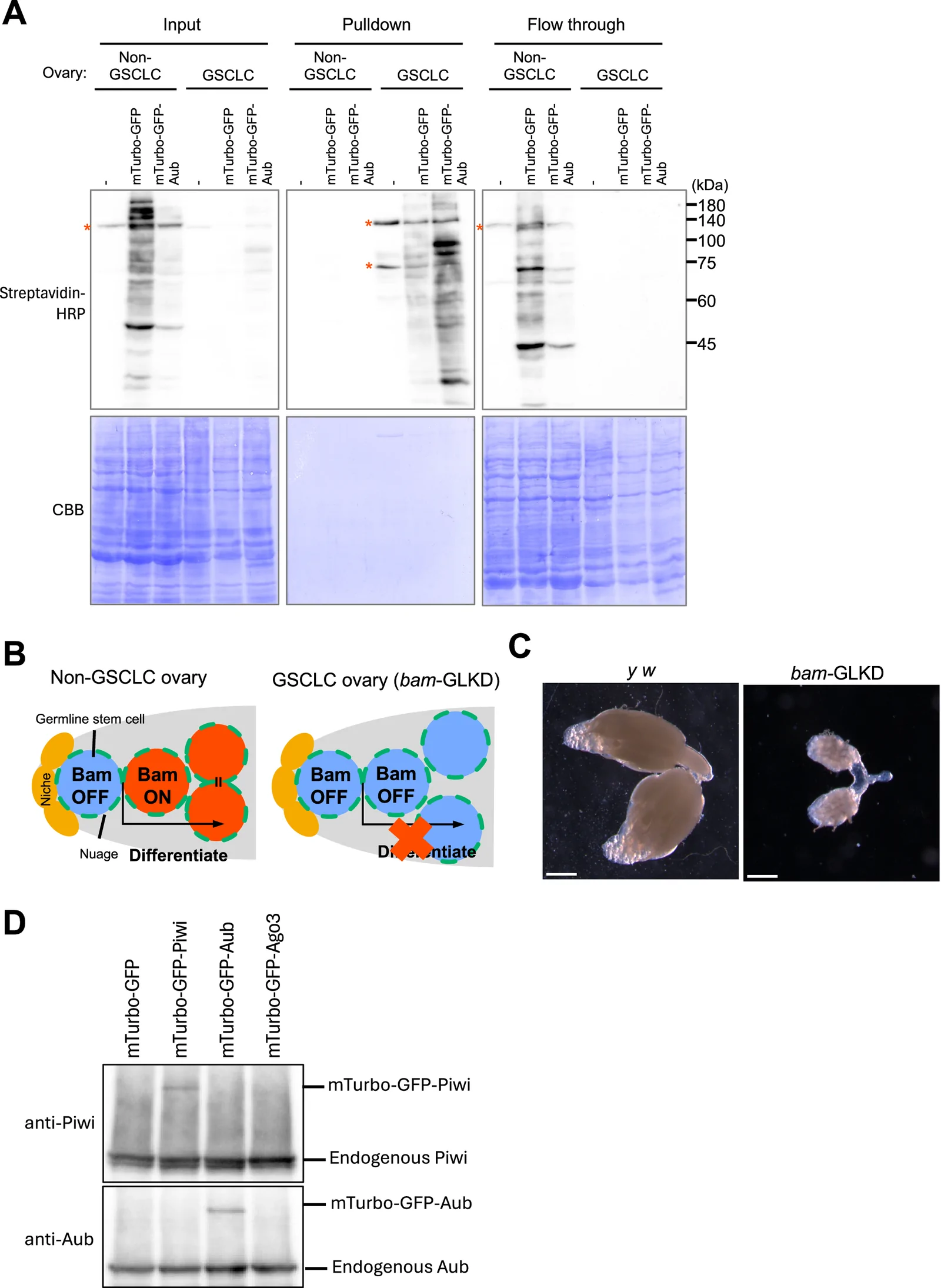
Germline PIWI proximity biotin labeling and purification. (**A**) Comparison of efficiency for biotinylation and biotinylated protein purification between non-GSCLC (germline stem cell-like cell) and GSCLC ovaries. Coomassie brilliant blue (CBB) staining serves as a protein loading control. Ovaries expressing mTurbo-GFP or mTurbo-GFP-Aub were analyzed in parallel with a negative control condition (without mTurbo protein expression). Asterisks: non-specific signals. (**B**) Schematic illustration for germline differentiation and the function of Bam. Left: germarium in wild-type, non-GSCLC ovaries. A germline stem cell (GSC, blue) divides to produce a daughter cell (red), which expresses Bam and starts the differentiation. Two to three GSCs are normally maintained in a germarium. Right: by *bam*-GLKD (germline knockdown), daughter cells cannot differentiate and thus accumulate as GSCLCs. (**C**) A pair of wild-type ovaries (*y w*, left) and that of *bam*-GLKD ovaries (right). Scale bar = 1 mm. (**D**) Immunoblotting images comparing the expression level of mTurbo-GFP-tagged Piwi or Aub with that of endogenous Piwi or Aub in GSCLC ovaries.

To characterize the proximity proteome of PIWI proteins using TurboID, we generated a series of transgenes encoding mTurbo-GFP-fused Piwi, Aub, or Ago3 (Fig. 1A). These transgenes were expressed in ovarian germline cells under the control of *nos* promoter activity (UASp/Gal4 binary system using NGT40;nos-Gal4 (NN) driver) (Rørth, 1998; Grieder et al, 2000). The biotinylation activities of individual fusion proteins were confirmed by detecting the streptavidin-HRP-dependent signals (Fig. EV1A). However, pulldown using streptavidin was inefficient, and biotinylated proteins largely remained in the flow-through, possibly due to interfering factors accumulated during differentiation. To circumvent this technical challenge, we depleted one of the differentiation factors, *bag of marbles* (*bam*) (McKearin and Spradling, 1990), by germline knockdown (GLKD), and collected germline stem cell (GSC)-like cells (GSCLCs) impaired in differentiation (Figs. 1A and EV1BC). Although TurboID in GSCLCs showed weaker biotinylation signals in the input, the pulldown efficiency was dramatically improved (Fig. EV1A).

In GSCLCs, mTurbo-GFP alone displayed a broad distribution across both the nucleus and cytoplasm (Fig. 1B). In contrast, mTurbo-GFP-PIWI proteins exhibited subcellular localization patterns consistent with those of their endogenous counterparts; Piwi accumulated in the nucleus, while Aub and Ago3 were enriched in the perinuclear nuage. The concordance suggests that N-terminal mTurbo-GFP tagging does not substantially disrupt the interaction networks of individual PIWI proteins. Supporting this notion, N-terminal mTurbo-FLAG fusion preserved Aub functions in male germline (Iki et al, 2023). Notably, the steady-state levels of fusion proteins were below those of endogenous counterparts (Fig. EV1D). mTurbo-GFP-Ago3 was the least stable and barely detectable in the input (Fig. 1C, anti-GFP), accounting for the weak streptavidin-HRP signals in Ago3-TurboID condition (Fig. 1C). Nevertheless, the streptavidin pulldown displayed distinct signal patterns, suggesting biotinylation-dependent enrichment of specific factors in each PIWI-TurboID condition.

**Figure 2:**
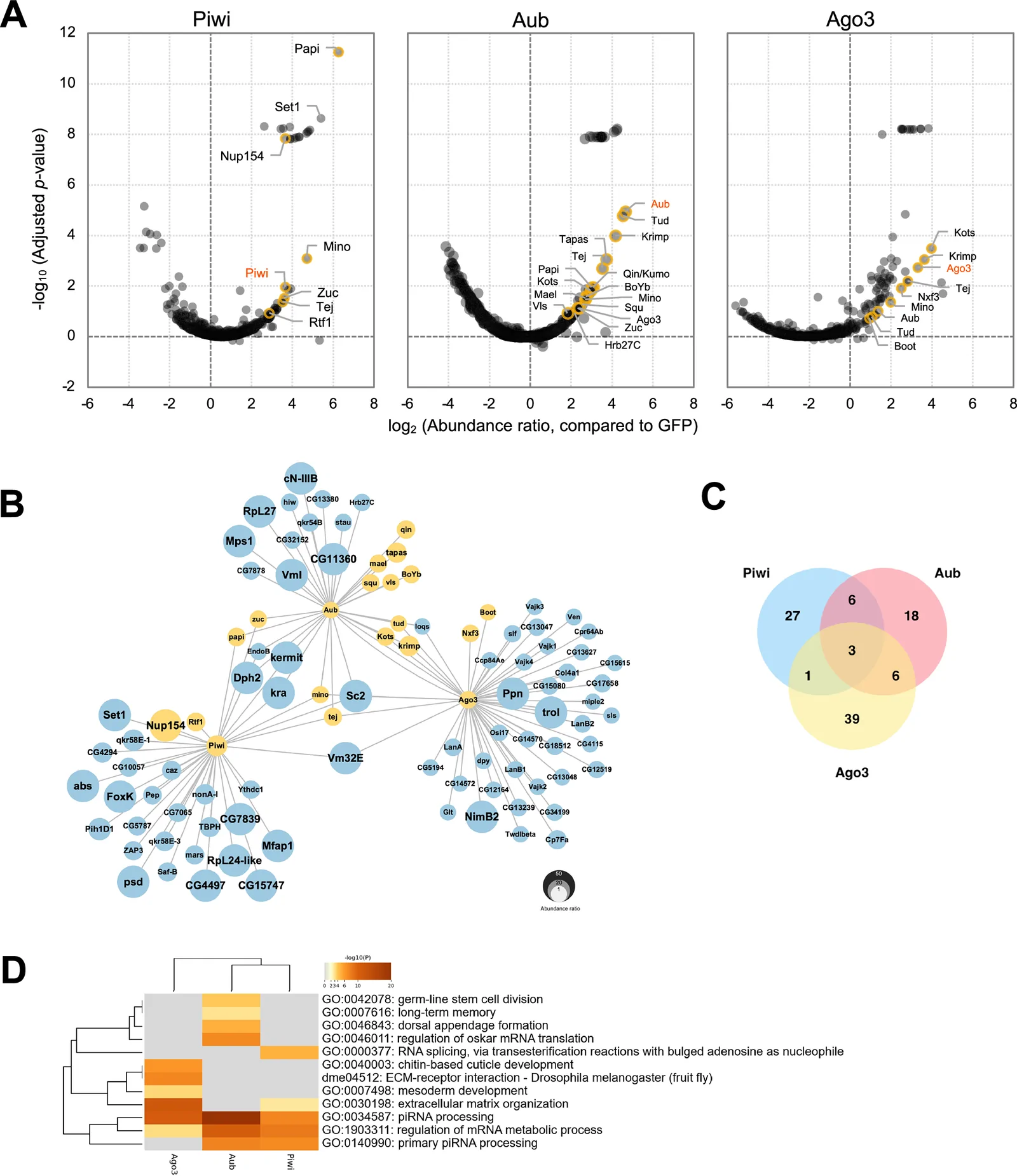
Proximity proteome of PIWI proteins in germline cells. (**A**) Volcano plots for the output of mass spectrometry (nanoLC-MS/MS) and label-free quantification (LFQ) for Piwi-, Aub-, or Ago3-TurboID. Mean values of abundance ratio (GFP-PIWI/GFP) and adjusted p from biological replicates (*n* = 2). See Fig. EV2 for the output from individual replicates. Statistics rely on a background-based *t* test. (**B**) Network diagram summarizing the proximity factors of individual PIWI proteins (Piwi, Aub, and Ago3). Yellow: factors reported in the piRNA pathway. Blue: factors not reported in the piRNA pathway. Circle size reflects the mean abundance ratio. Diagram drawn by CytoScape (Ono et al, 2025). (**C**) A Venn diagram shows the number of proximity factors shared by or unique to individual PIWI members. (**D**) GO enrichment analysis of PIWI proximity factors using MetaScape (Zhou et al, 2019). Hypergeometric test and Benjamini–Hochberg *P* value correction. Source data are available online for this figure.

**Figure EV2:**
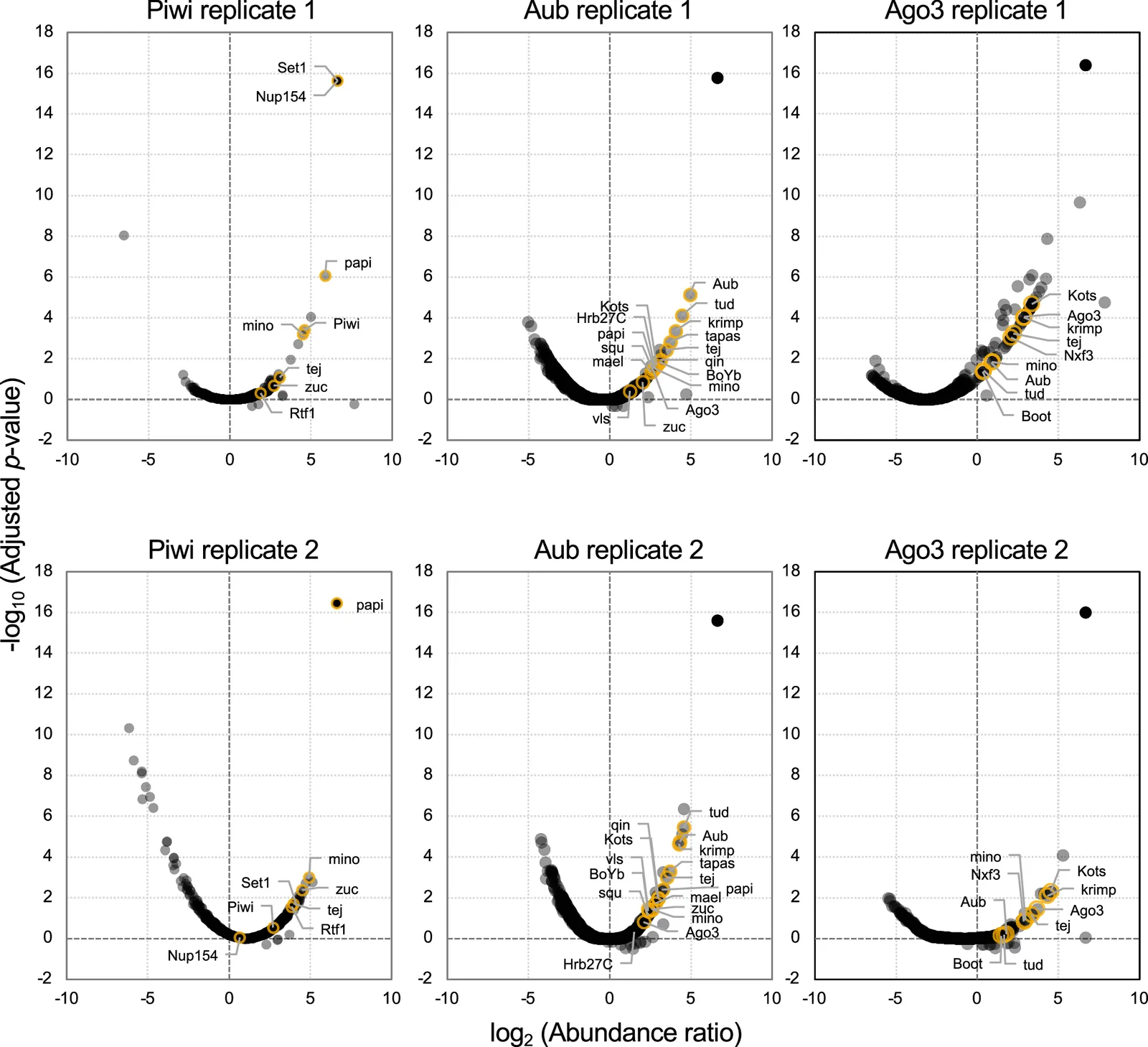
Germline PIWI proximity proteome in biological duplicates. Volcano plots for the proximity factors of Piwi, Aub, or Ago3 in individual biological replicates (*n* = 2). Abundance ratio: GFP-PIWI/GFP. Adjusted *p* is based on a background-based *t* test. Factors known to be involved in the piRNA pathway are indicated and highlighted with yellow circles.

Mass spectrometry analysis and the label-free quantification (LFQ) of streptavidin pulldown samples identified the proximity factors of individual PIWI proteins (mean abundance ratios >1 compared to control GFP-TurboID conditions in biological duplicates, and adjusted *P* < 0.05 in either one of the replicates) (Figs. 2A and EV2; Dataset EV1). These included 37, 33, and 49 factors for Piwi, Aub, and Ago3, respectively (Fig. 2BC). Notably, the identified proximity factors contained known piRNA pathway components (Fig. 2AB, highlighted in yellow, Table EV1), and the significant enrichment was confirmed by Gene Ontology (GO) analysis (Fig. 2D). These results suggest that the proteome reflects the physical and functional interactions between PIWI members and other factors in germline cells.

### Screening of PIWI proximity factors involved in piRNA biogenesis and TE silencing

**Figure 3:**
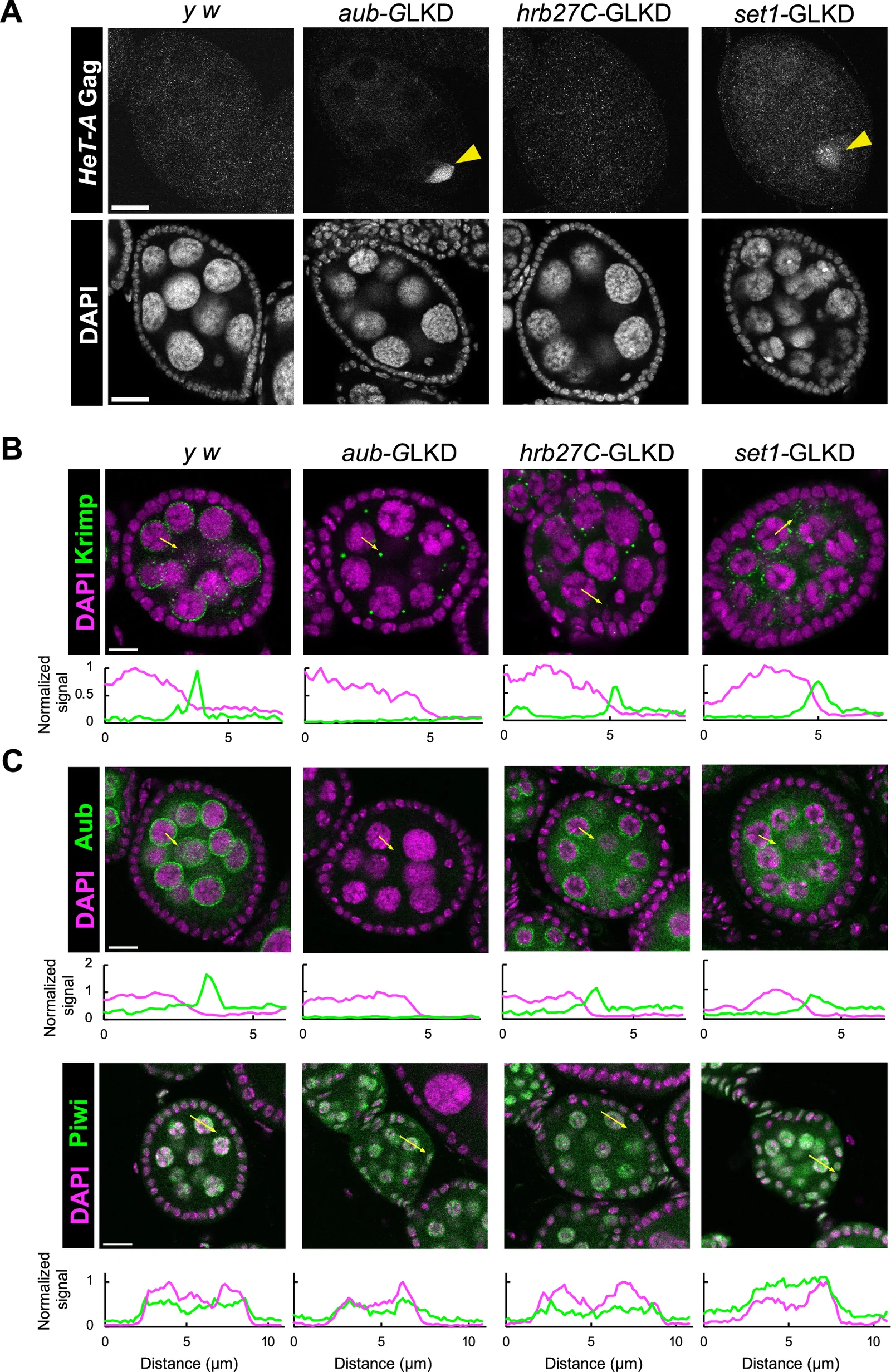
Germline knockdown (GLKD) screening of PIWI proximity factors. (**A**) Immunofluorescence signals from *HeT-A* Gag protein (top panels) in egg chambers of control (*y w*) or GLKD of indicated genes. DNA stained with DAPI (bottom panels). Arrowheads: Gag proteins accumulating around the oocyte. Scale bars = 20 μm. (**B**) Immunofluorescence signals of Krimp (green) in egg chambers of control (*y w*) or of indicated GLKD conditions. DNA stained with DAPI (magenta). Fluorescence signals along yellow arrows are plotted in the bottom panels (signals normalized by setting 1 for the highest value of DAPI). Scale bars = 10 μm. (**C**) Immunofluorescence signals of Aub or Piwi (green) in the egg chambers of the indicated conditions. Source data are available online for this figure.

**Figure EV3:**
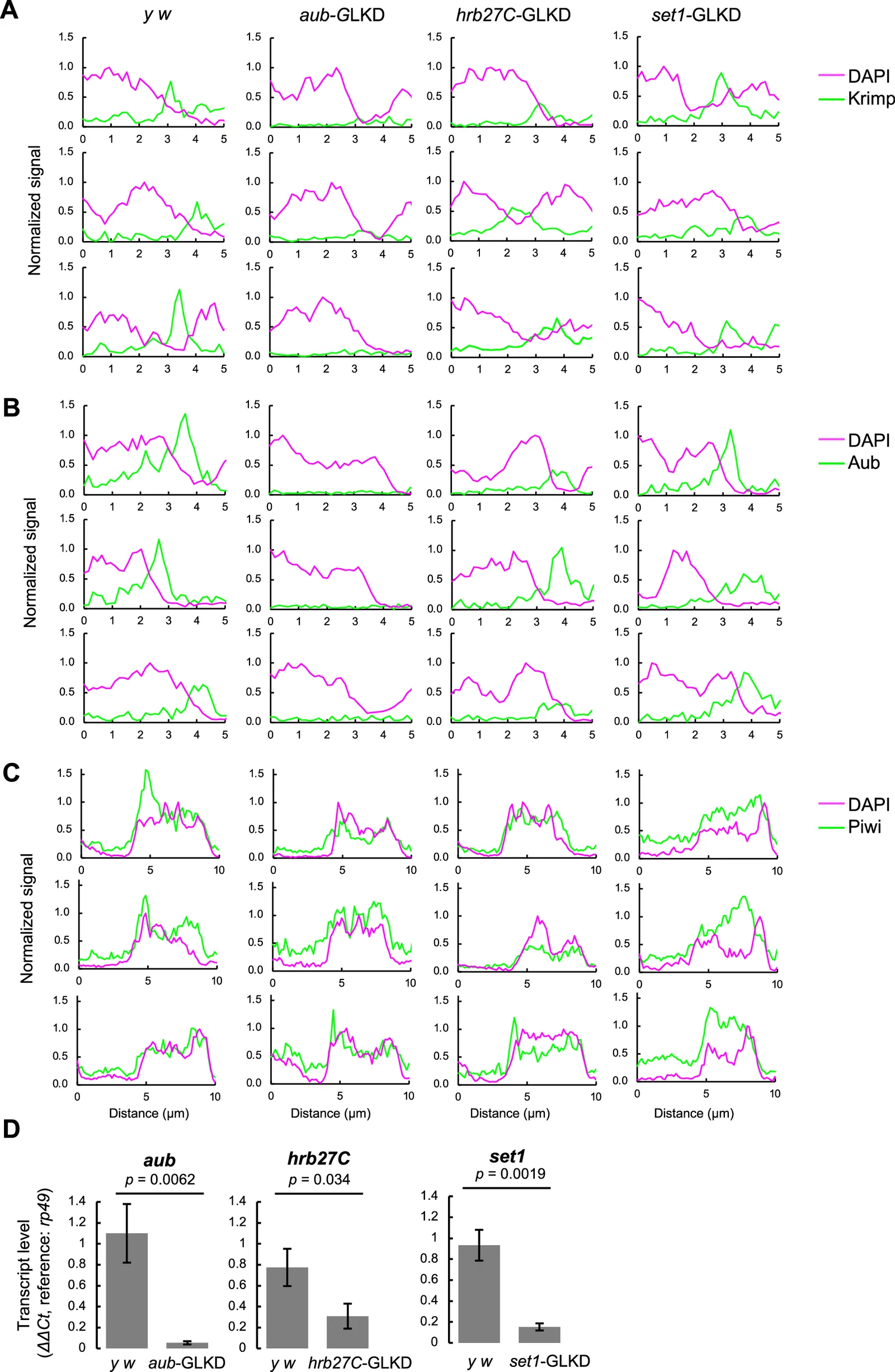
Germline knockdown (GLKD) efficiency and the effect on protein subcellular localization. (**A**–**C**) Plotting the fluorescence signals from egg chambers of the indicated genotypes, related to Fig. 3B. (**A**) Krimp (green) and DAPI (magenta), (**B**) Aub (green) and DAPI (magenta), (**C**) Piwi (green) and DAPI (magenta). (**D**) Transcript levels of *aub*, *hrb27C*, and *set1* in individual GLKD conditions (*ΔΔ*Ct method using *rp49* as reference). Error bars indicate standard deviation (SD) from biological replicates (*n* = 3). The center of error bars: average values. *P* values: unpaired Student’s *t* test.

Most factors identified in the proximity of PIWI members have not been functionally characterized in the piRNA pathway or TE silencing. Hence, we investigated the possible involvement of 54 factors, for which short hairpin-based RNAi lines were available, by germline knockdown (GLKD) screening using NN driver (Table EV2). Given that impaired piRNA biogenesis can be associated with nuage collapse and mislocalization of its components, we examined the localization of Krimp, a key scaffold of nuage (Lim and Kai, 2007; Webster et al, 2015; Sato et al, 2015). In parallel, we observed the accumulation of Gag protein encoded by a non-LTR retroelement *HeT-A*, as a readout of TE derepression (Shpiz et al, 2011). We confirmed that GLKD of *aub* leads to the accumulation of *HeT-A* Gag protein around the oocytes of developing egg chambers (Fig. 3A), and the loss of Krimp from perinuclear nuage and the formation of abnormal foci in the cytoplasm (Figs. 3B and EV3A).

GLKD screening identified several factors, including Hrb27C and Set1, the proximity factor of Aub and Piwi, respectively (Table EV2). Both factors are conserved across eukaryotes, and their molecular and physiological functions have been characterized. Hrb27C (also known as Hrp48) is an hnRNP family member required in GSC for the maintenance (Yan et al, 2014; Ables et al, 2016; Finger et al, 2023). Set1 is an enzyme acting inside the COMPASS complex responsible for di- and tri-methylation of histone H3 at lysine 4 (H3K4me2/3), a modification associated with transcription start sites (TSSs) for RNA polymerase (pol)II activation (Ardehali et al, 2011; Mohan et al, 2011; Wang et al, 2023a). We observed distinct phenotypes in *hrb27C*- and *set1*-GLKD. In *hrb27C*-GLKD, abnormal Krimp foci appeared in the cytoplasm, similar to *aub*-GLKD (Fig. 3B). However, *HeT-A* Gag protein was below the detectable level (Fig. 3A). In *set1*-GLKD, though abnormal Krimp foci formation was not clear, *HeT-A* Gag protein accumulated around the oocyte. In both *hrb27c*- and *set1-*GLKD conditions, perinuclear Krimp signals were weaker but still detectable (Fig. 3B). Consistently, Aub and Piwi did not show drastic changes in their localization (Figs. 3C and EV3BC). Of note, RT-qPCR confirmed target downregulation in individual GLKD conditions (Fig. EV3D).

### *set1* and *hrb27C* functionally interact with *piwi* and *aub*, respectively, to control TEs

**Figure 4:**
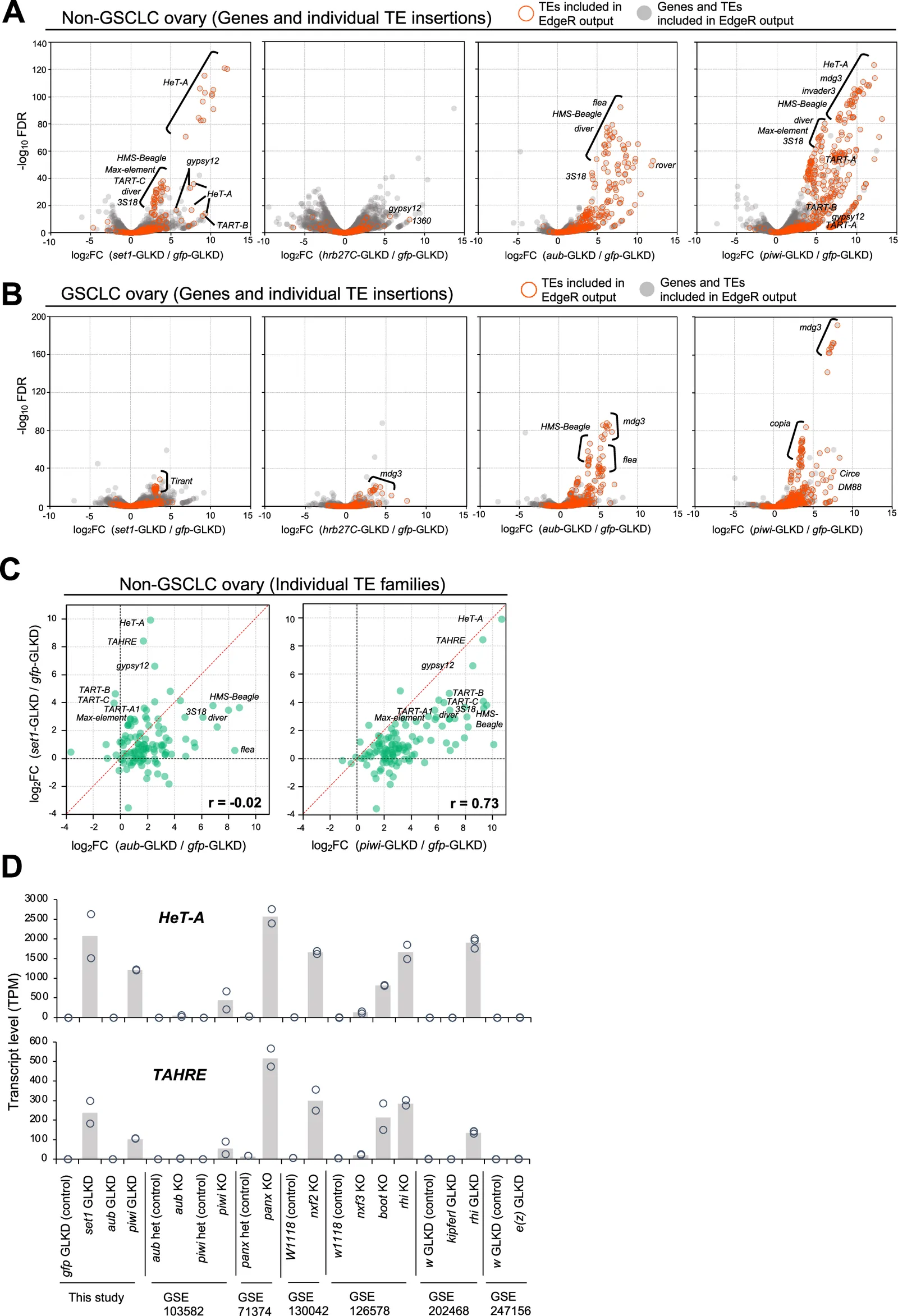
Germline knockdown (GLKD) effect on the ovary transcriptome. (**A**, **B**) Volcano plots comparing transcript levels (TPM) of genes and TE insertions in *set1*-, *hrb27C*-, *aub*-, or *piwi*-GLKD with those in control *gfp*-GLKD. Dots: genes (gray) and TE insertions (gray with red outline), included by EdgeR in differential expression analysis. Non-GSCLC (**A**) or GSCLC conditions (**B**). Biological replicates (*n* = 2). (**C**) Scatter plot comparing fold change (FC) of TE family transcript levels (TPM/TPM). *set1*-GLKD/*gfp*-GLKD compared with *aub*-GLKD/*gfp*-GLKD (left), or *piwi*-GLKD/*gfp*-GLKD (right). *r* = Pearson correlation coefficient. (**D**) Transcript levels (TPM) for *HeT-A* and *TAHRE* in different transcriptome datasets. GSE103582: *aub* or *piwi* knockout (KO), GSE71374: *panx* KO, GSE126578: *nxf3*, *boot*, *rhi* KO, GSE202468: *kipferl* or *rhi* GLKD, GSE247156: *e(z)* GLKD. Bars: mean values. Dots: individual values of biological replicates. Source data are available online for this figure.

**Figure EV4:**
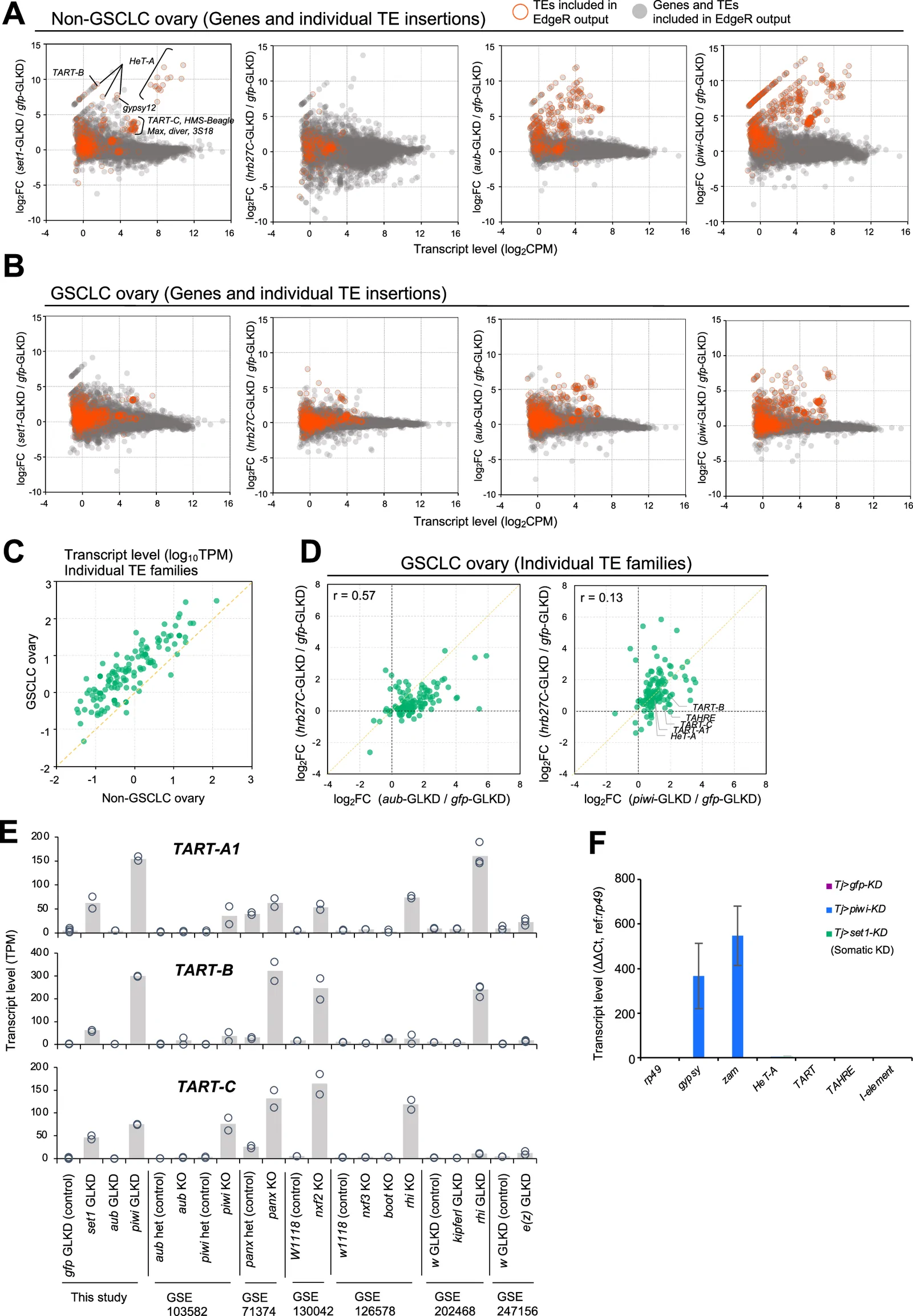
GLKD effect on the ovary transcriptome, and somatic KD effect on TEs. (**A**, **B**) MA plots comparing transcript levels (TPM) of genes and TE insertions in *set1*-, *hrb27C*-, *aub*-, or *piwi*-GLKD with those in control *gfp*-GLKD. Dots: genes (gray) and TE insertions (gray with red outline), included by EdgeR in differential expression analysis. Non-GSCLC (A) or GSCLC ovary conditions (**B**). (**C**) Scatter plot comparing transcript levels (TPM) of individual TE families (consensus sequences) in *gfp*-GLKD between non-GSCLC and GSCLC conditions. (**D**) Scatter plot comparing GLKD effect on transcript levels of individual TE families. Left: *hrb27C*-GLKD compared with *aub*-GLKD. Right: *hrb27C*-GLKD compared with *piwi*-GLKD. Fold changes (FC) from control *gfp*-GLKD are shown (TPM/TPM). *r* = Pearson correlation coefficient. (**E**) Transcript levels (TPM) for *TART-A1*, *TART-B*, and *TART-C*, in different transcriptome datasets. GSE103582 for *aub* and *piwi* knockout conditions. GSE71374 for *panx* knockout condition. Bars: mean values. Dots: individual values of biological replicates. (**F**) RT-qPCR measurement of TE transcript levels in ovaries upon somatic KD of *gfp*, *piwi*, or *set1* using *tj*-Gal4 driver. *ΔΔ*Ct method using *rp49* as reference. Error bars indicate ±SD from biological replicates (*n* = 3).

To investigate the global effect of Hrb27C or Set1 depletion in the germline on TE expression, we compared the transcriptome from *hrb27C*- or *set1*-GLKD ovaries with that from *gfp*-GLKD control ovaries (Figs. 4A and EV4A). In parallel, *aub*- and *piwi*-GLKD ovary transcriptomes were generated as references for TE derepression. Our analysis included the germline stem cell-like cell (GSCLC) condition in which the PIWI proximity proteome was characterized (Figs. 4B and EV4B). First, comparison of *gfp*-GLKD transcriptomes between non-GSCLC and GSCLC contexts revealed that TE transcript levels are generally elevated in GSCLCs (Fig. EV4C). This baseline increase may account for the relatively modest TE derepression observed for *aub*- or *piwi*-GLKD in the GSCLC setting (Fig. 4AB).

Hrb27C was identified as the proximity factor of Aub (Fig. 2). However, unlike *aub*-GLKD, *hrb27C*-GLKD did not result in clear TE derepression in non-GSCLC ovaries (Figs. 4A and EV4A; Dataset EV2). In contrast, TE derepression was evident in GSCLC ovaries (Figs. 4B and EV4B), and the patterns were positively correlated between *hrb27C-* and *aub*-GLKD conditions (*r* = 0.57, Fig. EV4D). These suggest a functional link between Hrb27C and Aub in GSCs.

For Set1 identified as the proximity factor of Piwi (Fig. 2), its GLKD caused remarkable derepression of a subset of TEs in non-GSCLC ovaries (Figs. 4A and EV4A; Dataset EV2). These *set1*-sensitive TEs included *HeT-A*, consistent with its Gag protein accumulation (Fig. 3), as well as *TART* family members, which together with *HeT-A* maintain telomeres as *HTT* array (Villasante et al, 2007). *TAHRE*, another *HTT* component, was not analyzed due to the lack of annotated insertions. In addition to *HTT*, several LTR retrotransposons, including *HMS-Beagle, Max-element*, *diver*, *3S18*, and *gypsy12*, exhibited robust derepression (Fig. 4A; Dataset EV2). This result was corroborated by analyses based on mapping reads to consensus sequences of individual TE families (Fig. 4C). Overall, the derepression profile of *set1*-GLKD showed a strong positive correlation with that of *piwi*-GLKD (*r *= 0.73), but not with *aub-*GLKD (*r *= −0.02). Consistent with this, re-analysis of a publicly available dataset (GSE103582) indicated that *HTT* members are more sensitive to the loss of *piwi* than that of *aub* (Fig. 4D, EV4E) (Teixeira et al, 2017). Moreover, *HTT* levels in *set1*-GLKD were comparable to those in the loss of Panoramix (Panx) and Nxf2, the cofactors of Piwi (GSE71374, GSE130042) (Sienski et al, 2015; Yu et al, 2015; Zhao et al, 2019), or Rhino (Rhi), the reader of H3K9me3 introduced by Egg/SetDB1 acting in the Piwi pathway (GSE126578, GSE202468, GSE247156) (ElMaghraby et al, 2019; Baumgartner et al, 2022; Akkouche et al, 2025). Of note, ovaries lacking Kipferl and Enhancer of Zeste (e(z)) cooperating with Rhi in specific contexts did not show robust *HTT* derepression. In contrast to the pronounced effect observed in germline, knockdown of *set1* in ovarian follicle cells using *traffic jam-*Gal4 did not derepress *zam* and *gypsy*, which are silenced by *piwi* in this somatic lineage (Fig. EV4F). These results support a functional link between Set1 and Piwi in germline TE silencing.

### Catalytic activity-independent role of Set1 in TE silencing

**Figure 5:**
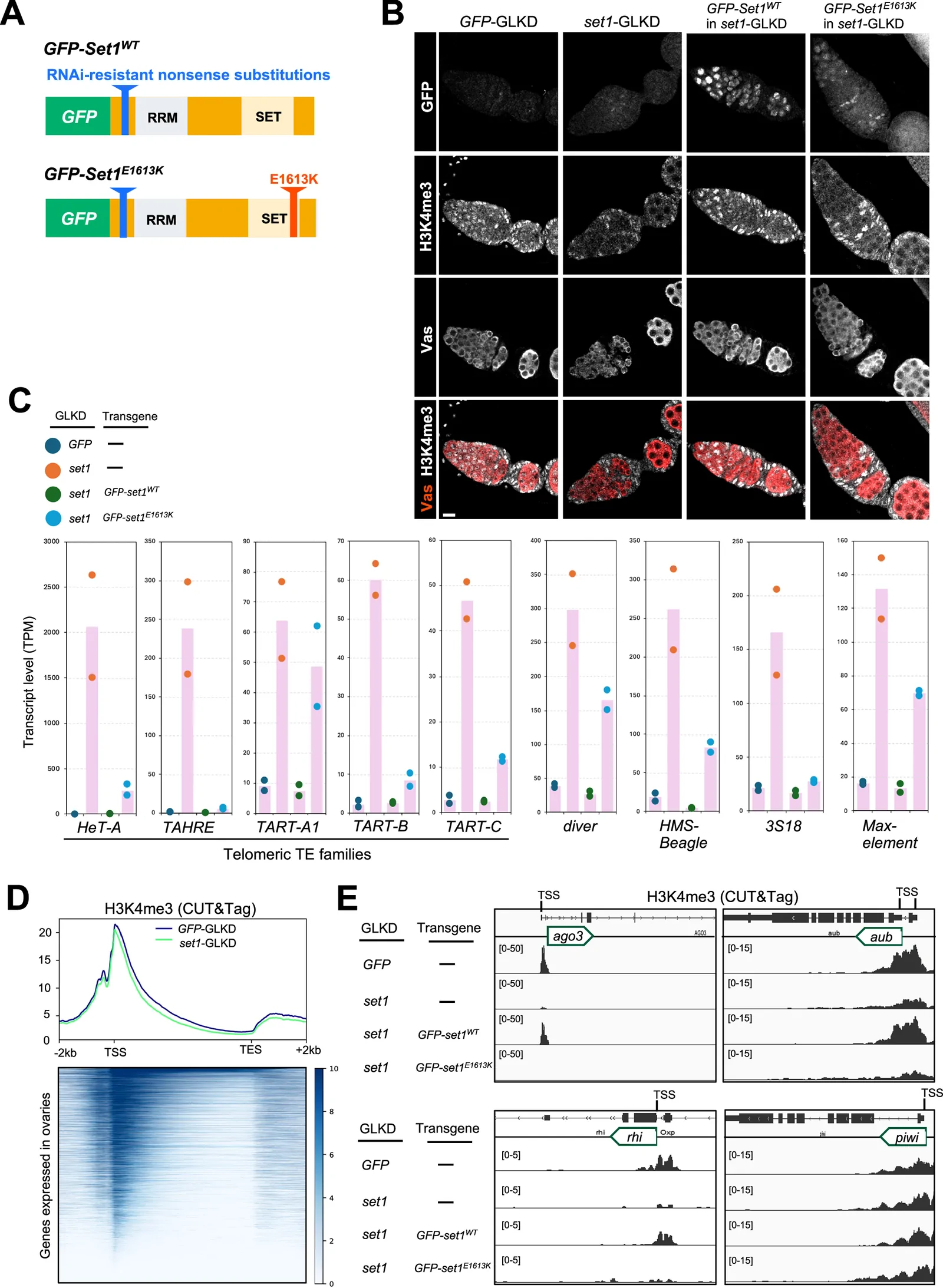
Effect of GFP-Set1 expression on H3K4me3 modification and TE expression in germline cells depleting endogenous Set1. (**A**) Schemetic of GFP-Set1 fusion proteins expressed by transgenes (Vidaurre et al, 2024). Substitution of 1613th glutamate (E) in Set1 polypeptide to lysine (K) abolishes the catalytic activity. Synonymous substitutions evade siRNA targeting. Both WT and E1613K are placed downstream of UASp and expressed in the germline using the NN driver. (**B**) GFP fluorescence (top) and immunofluorescence signals for H3K4me3 and Vas (bottom) in egg chambers. Vas marks germline cells. Scale bar =  10μm. (**C**) Transcript levels (TPM) of TE families in *gfp*-GLKD, *set1*-GLKD, and transgene expression conditions (*GFP-Set1*^*WT*^ or *GFP-Set1*^*E1613K*^ expressed in *set1*-GLKD). Bars: mean values. Dots: individual values of biological replicates (*n* = 2). (**D**) Plot profile and heatmap for anti-H3K4me3 CUT&Tag signals. Genes expressed in ovaries (*n* = 8196, genes analyzed in Fig. 4A) were analyzed. Gene bodies were scaled to 5 kb. TSS transcription start site, TES transcription end site. (**E**) Track view for H3K4me3 signals on indicated genes. Mean CPM from biological replicates (*n* = 2). Source data are available online for this figure.

The C-terminal SET domain of Set1 is responsible for H3K4me3 modification (Mohan et al, 2011; Ardehali et al, 2011; Wilson et al, 2002). To investigate whether this methyltransferase activity is required for TE silencing, we performed rescue experiments by expressing RNAi-resistant transgene encoding GFP-tagged wild-type Set1 (Set1^WT^) or a catalytically inactive variant (Set1^E1613K^) in germline cells depleted of endogenous Set1 (*set1*-GLKD) (Fig. 5A) (Vidaurre et al, 2024; Hallson et al, 2012). Both GFP-Set1^WT^ and GFP-Set1^E1613K^ proteins accumulated in the germline nuclei (Fig. 5B). Weaker GFP signals for E1613K variant suggest the reduced protein stability.

Transcriptome analysis revealed that *HTT* and other TE families were fully re-repressed by GFP-Set1^WT^ introduced in the *set1*-GLKD background, indicating the depletion of *set1* is causal for their derepression (Fig. 5C). Remarkably, although less effective than GFP-Set1^WT^, expression of GFP-Set1^E1613K^ led to substantial rerepression of TEs. This effect was most pronounced for *HTT*, with *TART-A1* as an exception (Fig. 5C). Together, these results suggest that Set1 plays a noncanonical, catalytic activity-independent role in TE silencing.

**Figure EV5:**
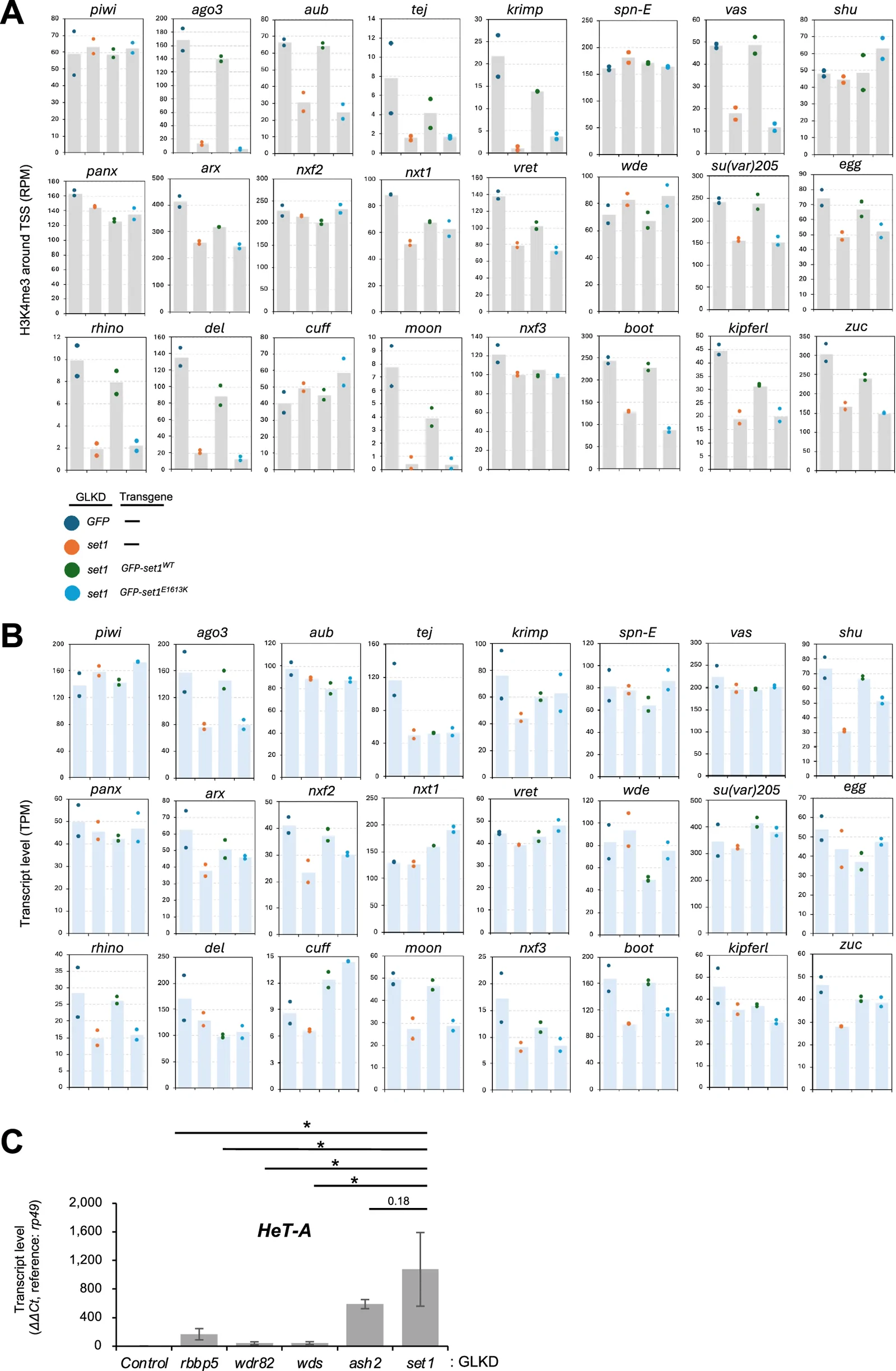
Effect of *set1*-GLKD on the expression of piRNA pathway factors, and effect of COMPASS subunit GLKD on *HeT-A* expression. (**A**) Bar graph showing mean H3K4me3 signal (RPM) around TSS ( ± 500 bp) of piRNA pathway genes. Dots: individual RPM from biological replicates (*n* = 2). (**B**) Bar graph showing mean transcript levels (TPM). Dots: individual TPM from biological replicates (*n* = 2). (**C**) RT-qPCR measurement of *HeT-A* transcript levels in ovaries depleting individual COMPASS subunits from germline cells. ΔΔ*Ct* method using *rp49* as a reference. Error bars: ±SD from biological replicates (*n* = 3). Asterisk: *P* < 0.05 (unpaired Student’s *t* test).

To support the above notion, we analyzed H3K4me3 signals in *set1*-GLKD and the transgene-rescue conditions. Ovary immunostaining showed that H3K4me3 signals were depleted from germline nuclei in *set1*-GLKD (Fig. 5B). Germline H3K4me3 signals were fully recovered by GFP-Set1^WT^, while no recovery was observed with GFP-Set1^E1613K^. We further employed CUT&Tag using whole ovaries to genome-wide characterize H3K4me3 signals. H3K4me3 formed peaks around the transcription start site (TSS) of genes expressed in ovaries (Fig. 5D, *n* = 8196, genes analyzed in Fig. 4A). The patterns and signals were comparable between *gfp*- and *set1*-GLKD conditions, when all ovary-expressed genes were considered, regardless of their germline specificity. Consistent with the immunostaining data, *set1*-GLKD depleted H3K4me3 signals around TSS of germline-restricted factors, including *ago3*, *aub*, and *rhi* (Figs. 5E and EV5A). H3K4me3 signals were restored by GFP-Set1^WT^, but not by GFP-Set1^E1613K^, confirming the catalytic inactivity of the E1613K variant. Of note, compared to the changes in H3K4me3 signals, those in steady-state transcript levels of piRNA pathway components were mild (Fig. EV5B). Further supporting the noncanonical function of Set1, germline depletion of individual COMPASS subunits led to variable degrees of *HeT*-A derepression, with *set1* and *ash2* depletions producing the most severe effect (Fig. EV5C). Taken together, these results argue against the notion that Set1 functions solely as an H3K4me3 writer in COMPASS and represses TEs simply by promoting the expression of piRNA pathway components.

### Loss of TE-targeting piRNAs in *set1-*depleted ovaries

**Figure 6:**
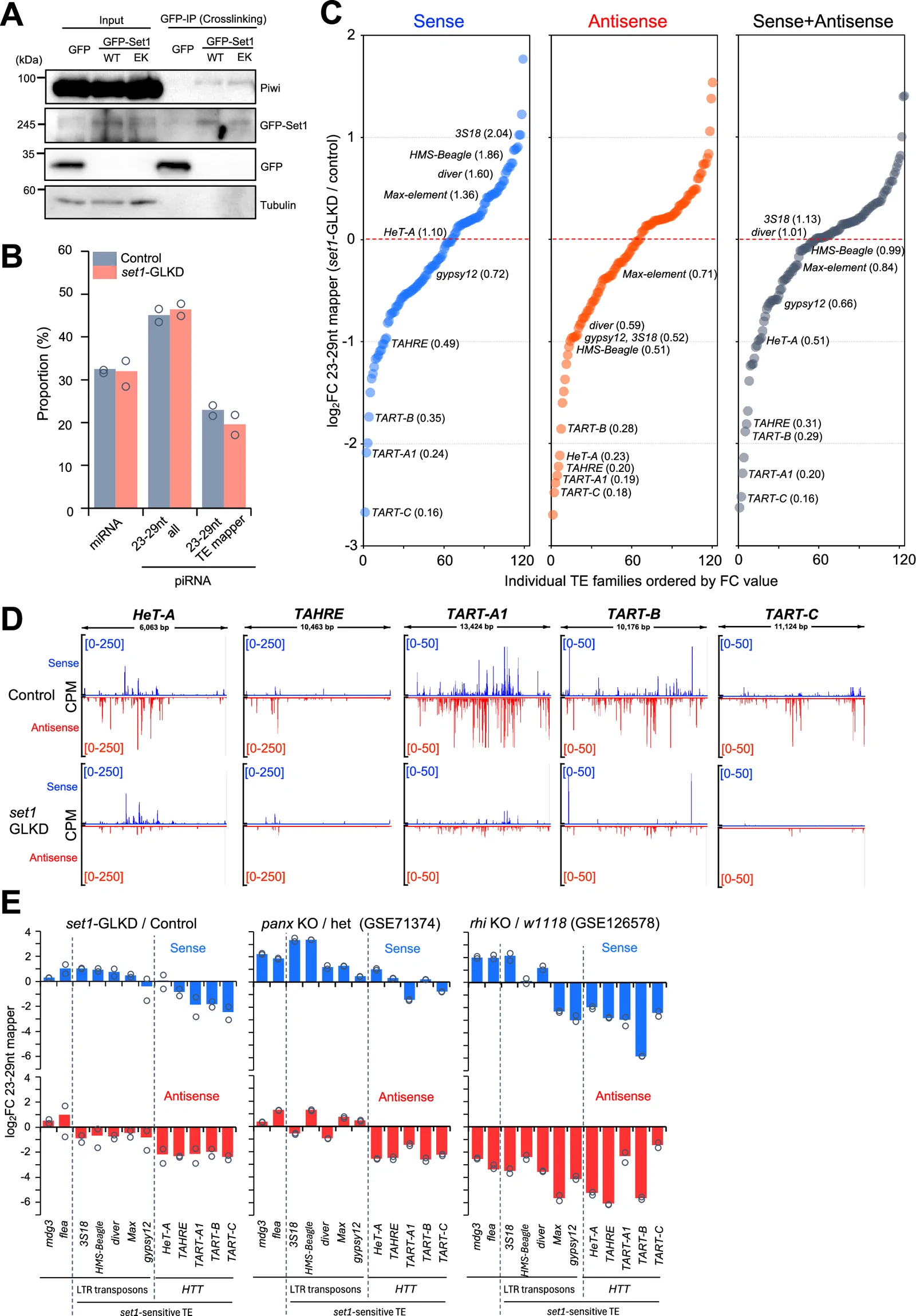
Co-precipitation of Piwi with Set1, and Set1 dependency of piRNA accumulation in ovaries. (**A**) Immunoprecipitation of GFP or GFP-Set1 proteins from ovaries using anti-GFP antibody (GFP-IP) after crosslinking treatment. Tubulin serves as a loading control. (**B**) Proportion (%) of miRNA and piRNA in ovarian small RNAs. miRNA: reads mapping to miRNA precursors. piRNA: 23–29-nt reads (excluding rRNA, tRNA, snoRNA, snRNA, and miRNA mappers), or 23–29-nt reads mapping to TE consensus sequences. Control: single replicate of *gfp*-GLKD and *GFP-Set1*^*WT*^-rescue conditions. *set1*-GLKD: biological duplicate of *set1*-GLKD condition. Bars: mean values, dots: individual values from biological replicates (*n* = 2). (**C**) Fold changes (FC) of piRNA levels in *set1*-GLKD compared to the control condition (RPM/RPM). Dots: individual TE families. TEs having few mappers (RPM < 5) were excluded. (**D**) Track view of piRNAs mapping to *HTT* family members. (**E**) Bar graph showing mean FC of TE-mapping piRNA levels in the mutants compared to the controls (RPM/RPM). Public data: *panx* KO from GSE71374, *rhi* KO from GSE126578. Dots: individual FC values from biological replicates (*n* = 2). Source data are available online for this figure.

**Figure EV6:**
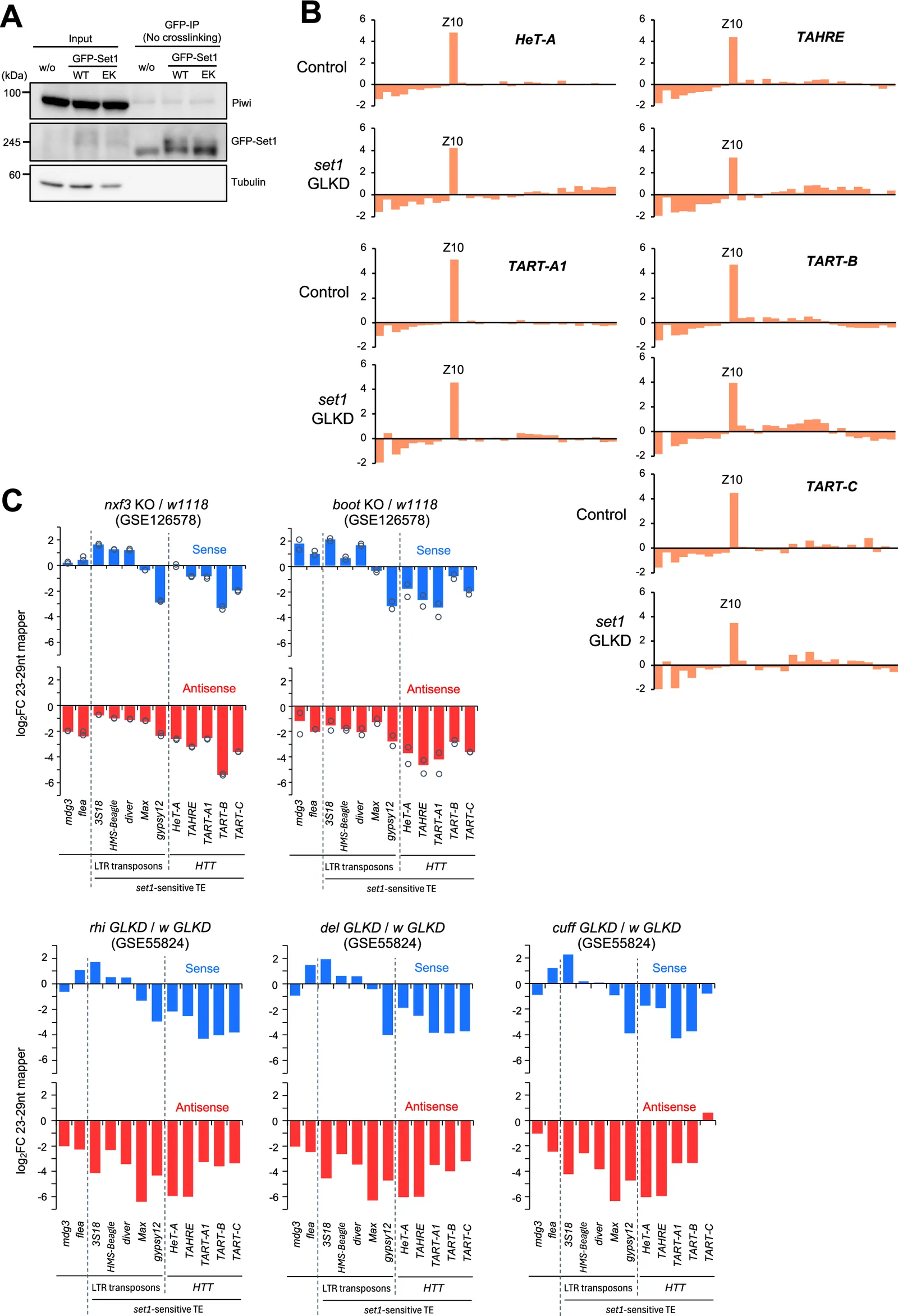
Immunoprecipitation of GFP-Set1 without crosslinking, effect of *set1*-GLKD on piRNA ping-pong signature, TE-mapping piRNA levels in ovaries lacking nxf3, boot, rhi, del, or cuff. (**A**) Immunoprecipitation of GFP-Set1 proteins from ovaries using anti-GFP antibody (GFP-IP) without crosslinking treatment. W/o: sibling control without GFP-Set1 expression. Tubulin serves as a loading control. (**B**) Ping-pong signature analysis on piRNAs mapping to telomeric TE families. Z10 scores (reflecting 10-nt overlap frequency within piRNA groups) were highlighted. Values are means of biological replicates (*n* = 2). (**C**) Bar graph showing mean fold change (FC) of piRNA abundance in mutants relative to their respective controls. Raw data from GSE126578 and GSE55824 were re-analyzed using the same pipeline. GSE126578 includes biological replicates (*n* = 2), and individual values are shown as dots.

TE derepression pattern suggests the functional link between Set1 and Piwi (Fig. 4). Moreover, consistent with their close proximity (Fig. 2), Set1 and Piwi interact physically, as Piwi co-precipitated with GFP-Set1^WT^ and GFP-Set1^E1613K^ expressed in germline cells (Fig. 6A). Notably, the interaction appears transient or weak, given the dependency on crosslinking (Fig. EV6A). These physical and functional links prompted us to examine whether piRNA expression is affected by Set1 depletion. Deep-sequencing of ovarian small RNAs showed that overall abundance of piRNAs (23–29-nt fragments or those derived from TE sequences) was largely unchanged in *set1*-GLKD (Fig. 6B). However, analysis at the level of individual TE families revealed a striking loss of piRNAs mapping to telomeric *HTT* members (Fig. 6C). Notably, antisense piRNAs, which directly target cognate TE transcripts, were reduced more severely than sense piRNAs. A prominent example is *HeT-A*, for which sense piRNAs were largely unaffected, whereas antisense species were nearly lost (Fig. 6CD). An antisense-biased reduction of piRNAs was also observed for other Set1-controlled TEs, including *3S18*, *gypsy12, Max-element*, *diver*, and *HMS-Beagle* (Fig. 6CE). Despite the loss, the ping-pong signature (10-nt overlap frequency) of piRNAs was preserved (Fig. EV6B). This is consistent with the maintenance of nuage in *set1*-GLKD (Fig. 3B). Supporting a link to the Piwi pathway, re-mining of public datasets revealed a similar antisense-biased reduction in *HTT*-mapping piRNAs in *panx*-deficient ovaries (Fig. 6E). Ovaries lacking Rhi, a key activator of piRNA precursor transcription, also exhibited an antisense-biased piRNA reduction; however, the effect was extended beyond Set1-dependent TEs. Similar broad effect was observed upon the depletion of Rhi cofactors, Del and Cuff, and the loss of Nxf3 or Boot, exporting piRNA precursor transcripts (GSE71374, GSE126578, and GSE55824) (Figs. 6E and EV6C). Together, these results suggest that Set1 is involved in the Piwi pathway for piRNA biogenesis. Set1 is particularly important for the production of piRNAs targeting telomeric TEs.

### Set1 binds TE sequences and localizes to subtelomeric piRNA cluster loci

**Figure EV7:**
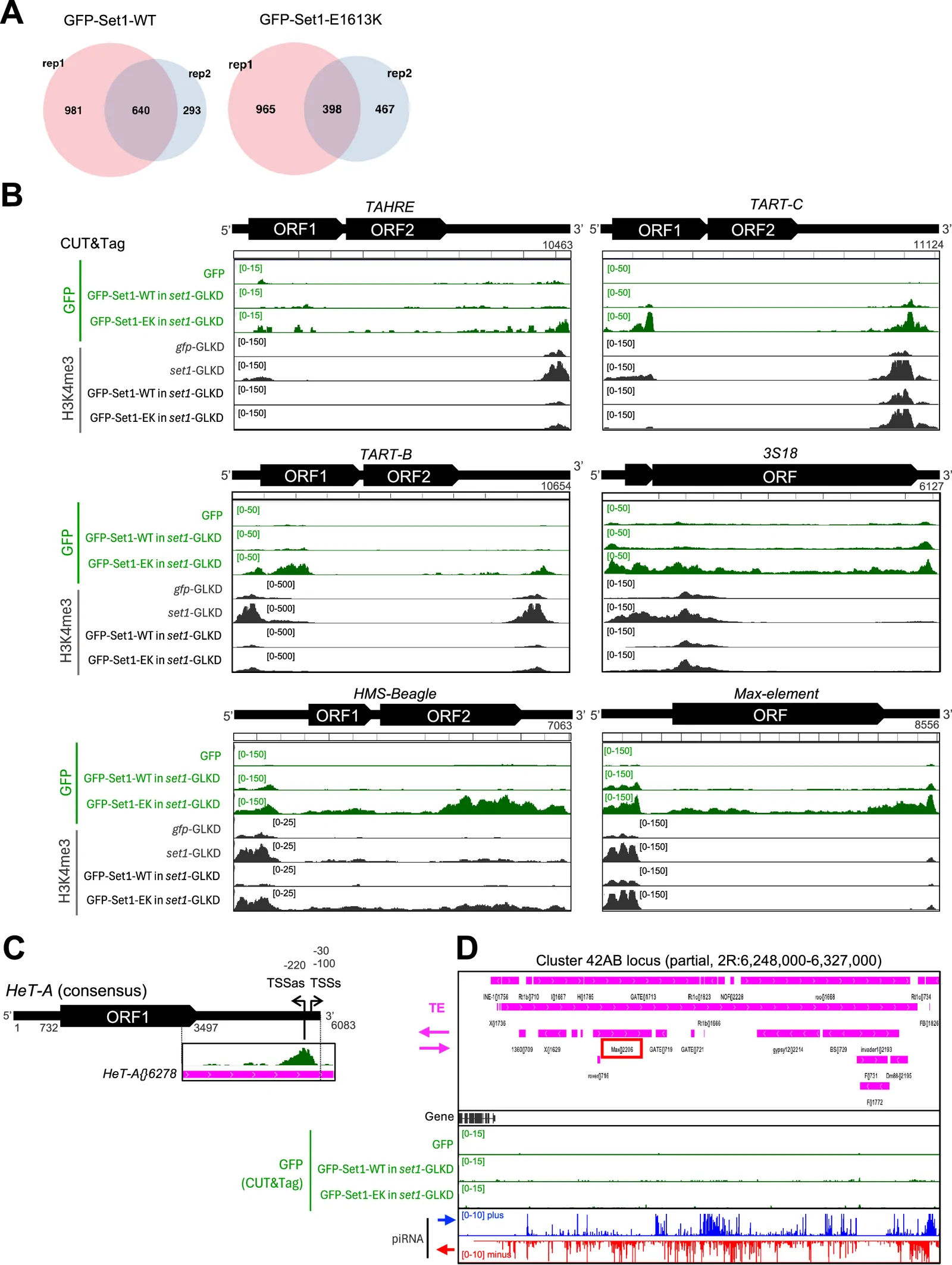
Genome-wide analysis of Set1 binding by CUT&Tag. (**A**) Venn diagram showing overlap of Set1-binding peak-associated genes and TEs between biological replicates (*n* = 2). Peak called by MACS2 comparing GFP-Set1^WT^ and GFP, or GFP-Set1^E1613K^ and GFP (*p* < 0.01, Poisson test). (**B**) Track view for CUT&Tag signals of GFP proteins (green) and H3K4me3 (black) on TE consensus sequences. ORFs were predicted by ORF Finder. (**C**) Alignment of *HeT-A{}6278* with GFP-Set1^E1613K^ binding signals to *HeT-A* consensus sequence. (**D**) Track view for anti-GFP CUT&Tag signals at *42AB* locus (partial). Blue and red: piRNAs mapped to the plus and minus strands, respectively.

**Figure 7:**
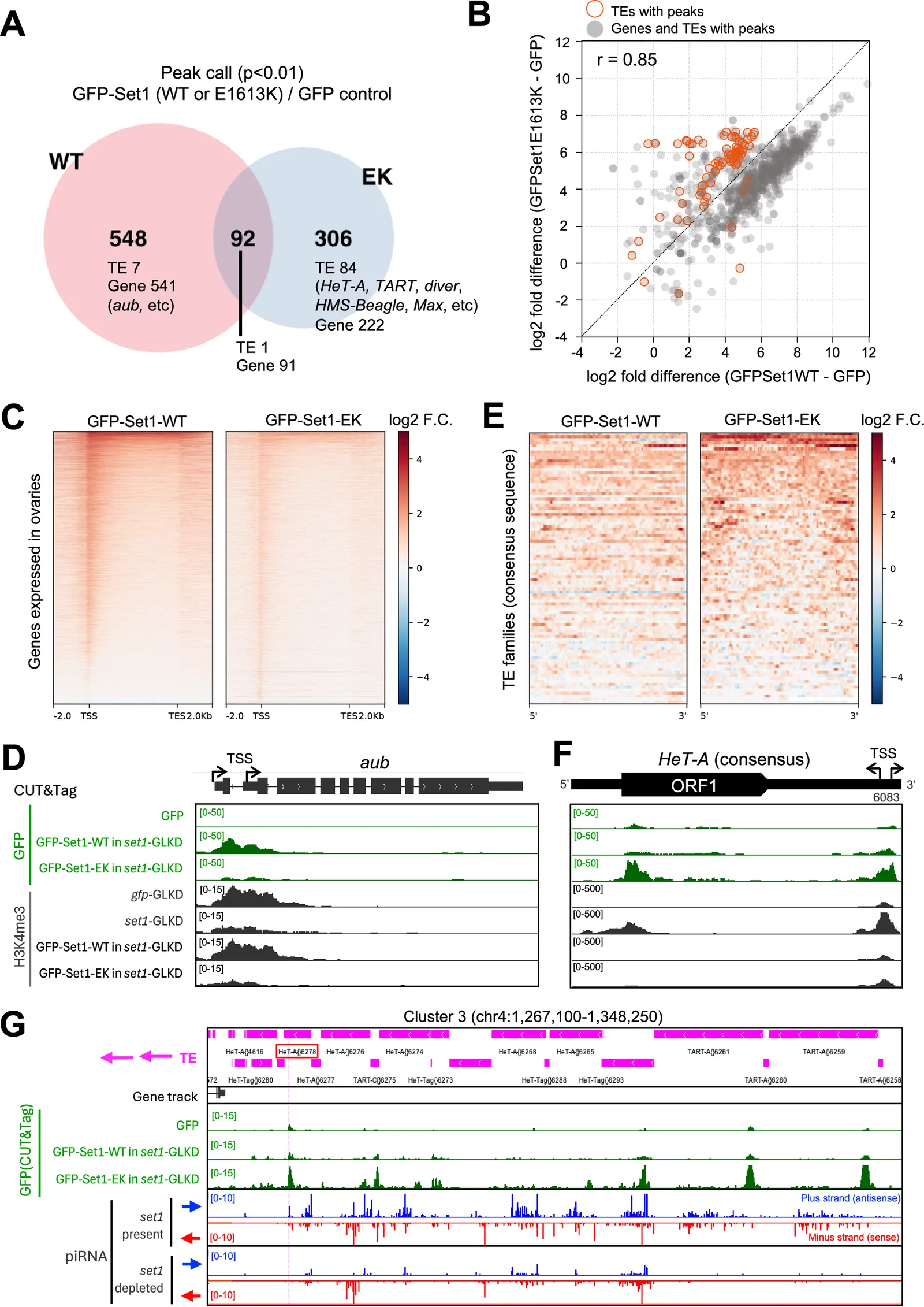
Genome-wide analysis of Set1 localization. (**A**) Venn diagram for genes and TEs having GFP-Set1^WT^- or GFP-Set1^E1613K^-binding peaks. Peaks called by MACS2 (*p* < 0.01, estimated by Poisson distribution model). (**B**) Scatter plot comparing fold differences of CUT&Tag signals (GFP-Set1 - GFP) on genes and TEs identified by peak calling. Mean values of biological replicates (*n* = 2). Signals on exons of genes and TEs were counted. *r*: Pearson correlation coefficient. (**C**) Heatmap shows GFP-Set1 enrichment within genes expressed in ovaries (*n* = 8196, genes analyzed in Fig. 4A). Log2 FC of mean CPM + 1 pseudocount (GFP-Set1/GFP). Gene bodies were scaled to 5 kb. (**D**) Track view on *aub* gene showing signals from anti-GFP (green) or anti-H3K4me3 (black) CUT&Tag. Mean CPM of biological replicates (*n* = 2). (**E**) Heatmap shows GFP-Set1 localization on TE consensus sequences. >3 kb TE families were selected. Log2 FC of mean CPM + 1 pseudocount (GFP-Set1/GFP). (**F**) Track view on *HeT-A* consensus sequence showing signals from anti-GFP (green) or anti-H3K4me3 (black) CUT&Tag. (**G**) Track view at the subtelomeric cluster 3 locus. Blue and red: piRNAs mapping to genomic plus and minus strands, respectively. Source data are available online for this figure.

How does Set1 contribute to the expression of antisense piRNAs? To address this question, we analyzed the chromatin binding by GFP-Set1 expressed in endogenous Set1-depleted germline cells (*set1*-GLKD), by CUT&Tag using anti-GFP antibodies. In parallel with GFP-Set1^WT^, GFP-Set1^E1613K^ was included in the analysis, given the functionality in TE silencing (Fig. 5). Comparison between GFP-Set1 and GFP control conditions identified genes and TEs associated with Set1-binding peaks (*p* < 0.01, Fig. EV7A). Notably, these genes and TEs showed limited overlap between Set1^WT^ or Set1^E1613K^ (Fig. 7A). However, this does not indicate their binding targets are distinct; rather, it reflects differential affinity between Set1^WT^ and Set1^E1613K^ to shared targets (*r* = 0.85, Fig. 7B). Consistent with its role as H3K4me3 writer and transcriptional coactivator, targets identified from Set1^WT^ binding peaks were predominantly endogenous genes (541 of 548, Fig. 7A). These peaks appeared around TSS and coincided with *set1*-dependent H3K4me3 signals, as exemplified with *aub* (Fig. 7CD). Targets identified from Set1^E1613K^ binding peaks were enriched with TEs (Fig. 7A, 84 of 306), including *HeT-A, TART*, *diver*, *HMS-Beagle*, and *Max-element*, regulated by Set1 (Figs. 4A and 5C). The signals for Set1^E1613K^ were weaker on genes but stronger on TE insertions, compared to those for Set1^WT^ (Fig. 7BCE). The signals did not form a single peak on TE consensus sequences (Fig. 7E). Rather, signals could be bipolarized: one spanning 5’UTR-ORF1 and another in 3’UTR, as exemplified with *HeT-A* and other Set1-controlled TEs (Figs. 7F and EV7B). Collectively, these results revealed the affinity of Set1 to TE sequences.

We mention that H3K4me3 signals on TEs behaved differently from those at gene TSS: they increased upon Set1 depletion and decreased upon expression of both Set1^WT^ and Set1^E1613K^, indicating a lack of correlation with Set1’s catalytic activity (Figs. 7D,F and EV7B). Instead, H3K4me3 signals on TEs were associated with the transcript abundance (Fig. 5C), suggesting that these marks are deposited through transcription of TEs either by residual Set1 in *set1*-GLKD or through Set1-independent mechanisms (Ardehali et al, 2011).

**Figure EV8:**
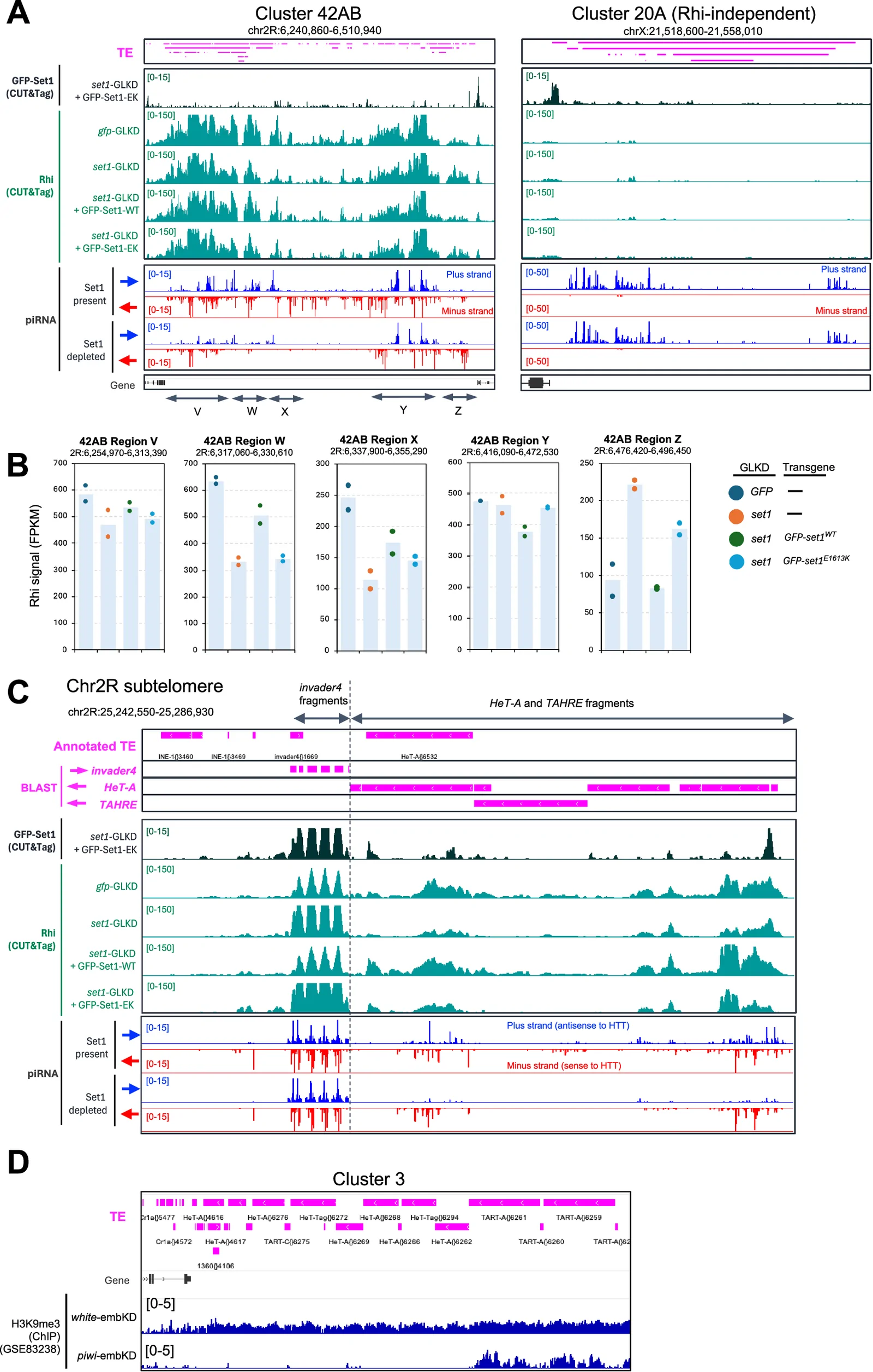
Effect of Set1 depletion on Rhi localization at piRNA cluster loci, and effect of Piwi depletion on H3K9me3 deposition in subtelomeric cluster 3. (**A**) Track view at 42AB or 20A locus. Light green: signals from anti-Rhi CUT&Tag. Dark green: GFP-Set1-EK signals (anti-GFP CUT&Tag). Blue and red: piRNAs mapped to genomic plus and minus strands, respectively. (**B**) Quantification of anti-Rhi CUT&Tag signals. Bars: mean FPKM, dots: individual FPKM, from biological replicates (*n* = 2). Analyzed regions within 42AB are indicated in (**A**). (**C**) Track view in the subtelomeric region on chromosome 2R. (**D**) Track view at subtelomeric cluster 3 showing H3K9me3 signal (mean CPM from biological replicates (*n* = 2)). embKD: knockdown at embryonic stages. Raw data: GSE83238.

3’UTRs of *HTT* members possess promoter and TSS for bidirectional transcription (Fig. 7F) (Danilevskaya and Arkhipova, 1997; Radion et al, 2017; Maxwell et al, 2006). Intriguingly, *HTT* 3’UTRs interacting with Set1 are maintained as truncated insertions in subtelomeric piRNA cluster loci. *HeT-A{}6278* at cluster 3 on chromosome 4 is one of those 3’UTR fragments, with Set1 signal peaked on the putative antisense TSS (Fig. EV7C). piRNA hot spot starting from this 3’UTR fragment implies the involvement in piRNA precursor transcription (Fig. 7G). Set1 showed affinity to other sites on cluster 3, which is in contrast to non-telomeric clusters lacking, if not any, Set1 binding (Figs. EV7D and 8). Insertions from *set1*-sensitive LTR retrotransposons are rare within major piRNA clusters. *Max{}2206* is an exception inserted in 42AB cluster, but it did not accumulate Set1 (Fig. EV7D). Hence, Set1 binding to cluster loci is biased toward subtelomeric regions, whose piRNA production is extremely *set1*-dependent (Fig. 7G). Set1 binding to LTR retrotransposons may instead reflect its affinity for potentially mobile copies located outside piRNA clusters (Wang et al, 2018). Collectively, these results suggest that Set1 binding to TE sequences underlies piRNA biogenesis and TE silencing in germline cells.

### Set1 controls Rhi localization on piRNA cluster loci

**Figure 8:**
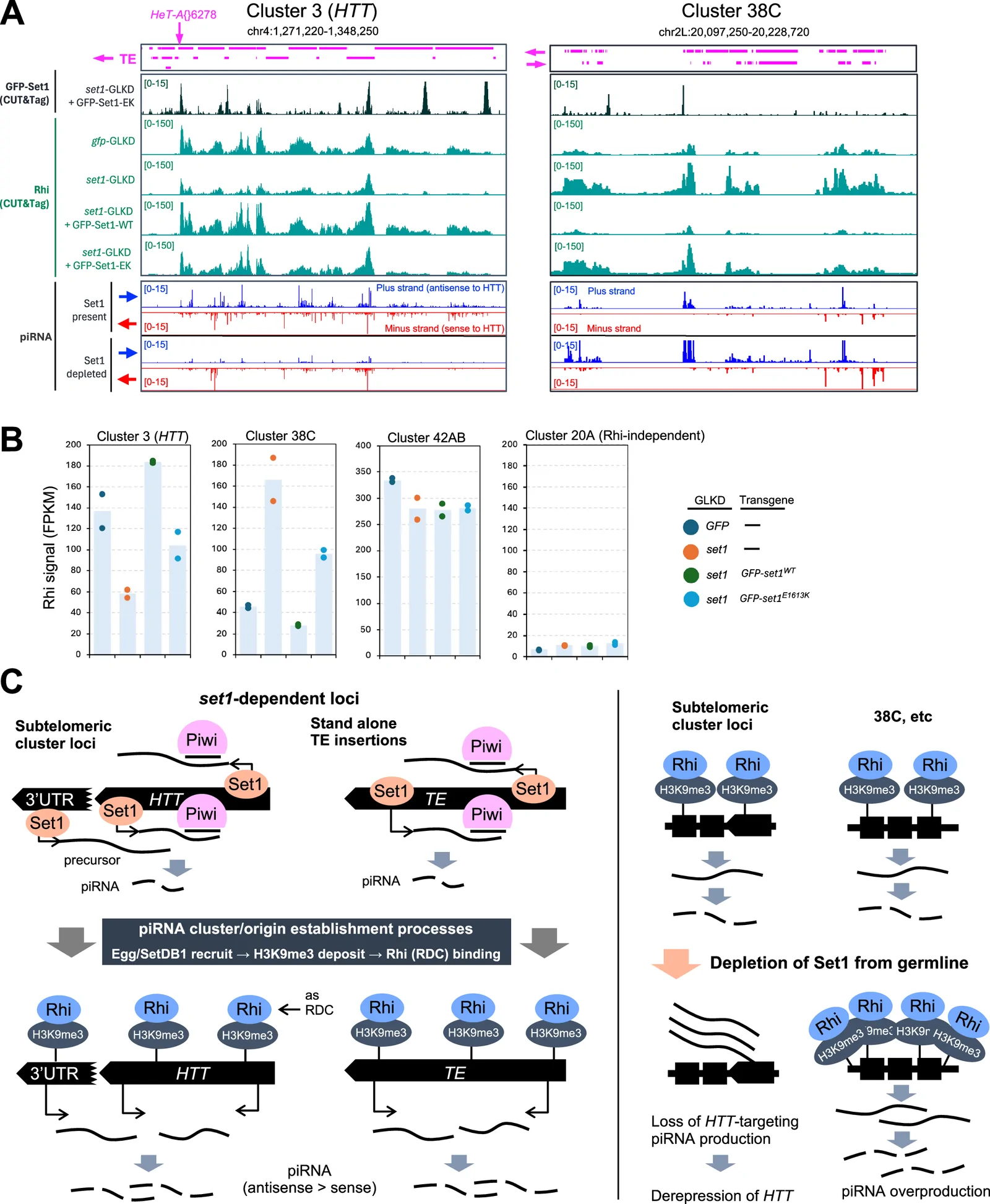
Effect of Set1 depletion on Rhi localization to piRNA cluster loci. (**A**) Track view at subtelomeric cluster 3 or 38C locus. Light green: signals from anti-Rhi CUT&Tag. Dark green: GFP-Set1-EK signals in *set1*-GLKD (anti-GFP CUT&Tag). Blue and red: piRNAs mapped to genomic plus and minus strands, respectively. Mean CPM from biological replicates (*n* = 2). (**B**) Quantification of anti-Rhi CUT&Tag signals. Bars: mean FPKM (fragments per kilobase per million mapped reads), dots: individual FPKM, from biological replicates (*n* = 2). (**C**) Working model. Set1 interacting with *HTT* sequences cooperates with Piwi for piRNA cluster formation in subtelomeric regions. Set1 provides target templates for Piwi-piRNA complexes and/or precursor transcripts for piRNA production. Fragmented *HeT-A* 3’UTR retaining antisense TSS plays a crucial role in the latter process. Cooperation between Set1 and Piwi promotes H3K9me3 deposition and Rhi accumulation. Once this piRNA cluster status is established, Set1 and Piwi become dispensable. Similar mechanisms operate in the silencing of stand-alone TEs. Germline cells lacking the noncanonical functions of Set1 not only fail to establish piRNA clusters at subtelomeric loci but also exhibit aberrant Rhi distribution and piRNA overproduction at certain loci. Source data are available online for this figure.

Germline piRNA biogenesis largely relies on Rhi, a germline-specific heterochromatin protein (HP)1 paralogue and H3K9me3 reader, crucial for piRNA precursor transcription from cluster loci. Given the requirement of both Set1 and Rhi in *HTT*-targeting piRNA production (Fig. 6E), we examined how Set1 interacts with Rhi. Consistent with previous ChIP analyses (Mohn et al, 2014), our CUT&Tag showed Rhi localization at major cluster loci including 38C and 42AB, as well as subtelomeric cluster 3 accumulating *HTT* fragments. Contrastively, Rhi signal was near absent at 20A whose unistrand piRNA biogenesis is Rhi-independent (Figs. 8A,B and EV8A, see *gfp*-GLKD control). At cluster 3 locus, several peaks of Rhi overlapped with those of Set1, including the one on the putative antisense TSS in *HeT-A{}6278*.

Depletion of Set1 from the germline (*set1*-GLKD) caused a striking reduction in Rhi signals at the cluster 3 locus (Fig. 8A,B). Rhi accumulation was fully restored with GFP-Set1^WT^, and less efficiently with GFP-Set1^EK^, consistent with *HTT* rerepression by the transgene products (Fig. 5C). Remarkably, in contrast to cluster 3, *set1*-GLKD caused Rhi overaccumulation at the 38C locus, which was accompanied by piRNA overproduction (Fig. 8A). The aberrant induction was fully and partially rescued by GFP-Set1^WT^ and GFP-Set1^EK^, respectively. At 42AB, total Rhi signals were largely unchanged, but local fluctuation was observed within the locus (Fig. EV8AB). Similar but more drastic Rhi signal fluctuation was observed in the subtelomeric region of chromosome 2R. There, Rhi was depleted from the repeat of *HeT-A* and *TAHRE* in *set1*-GLKD, while enriched in that of *invader4* fragments nearby (Fig. EV8C). The redistribution of Rhi in *set1*-GLKD is unlikely to be a mere consequence of attenuated Rhi expression (Fig. EV5AB).

Developmental stage-specific knockdown approach demonstrated that Piwi at the embryonic stage initiates piRNA cluster formation by accumulating H3K9me3 (Akkouche et al, 2017). We re-mined the public data (GSE83238) and found that H3K9me3 accumulation at subtelomeric cluster loci also relies on Piwi (Fig. EV8D). Together, these findings support a model in which Set1 binding to *HTT* cooperates with Piwi to mediate the formation of piRNA clusters in subtelomeric regions (Fig. 8C).

## Discussion

To deepen the understanding of piRNA-directed TE silencing, this study explored the proximity factors of individual PIWI proteins expressed in female germline cells. The proteome provided additional clues supporting the known interactions, and, more importantly, revealed otherwise unrecognized links between factors in piRNA biogenesis. First, we point out that Zucchini (Zuc), an endonuclease on the mitochondrial outer membrane, was identified in the proximity of both Aub and Piwi. These three proteins are together involved in phased piRNA production (Ge et al, 2019), but their physical links have not been reported in ovaries. It should be noted that a study conducting TurboID using Zuc as bait did not find Aub or Piwi in its proximity (Nguyen et al, 2023). This might be due to the difference in drivers (matalfa-Gal4 vs nos-Gal4), stages of germline cells (differentiating cells vs stem-cell-like cells), or bait proteins (Zuc vs PIWI proteins). In addition to Zuc, our data also showed that Nxf3 and Bootlegger (Boot) mediating the export of piRNA precursors are in the proximity of Ago3, but not of Aub (Kneuss et al, 2019; ElMaghraby et al, 2019). The selective proximity supports the model whereby precursor transcripts delivered by Nxf3 and Boot from nucleus to nuage in cytoplasm are targeted by Ago3-piRNA complexes (Wang et al, 2023b).

Hrb27C emerged as a functionally relevant partner of Aub. As a member of the hnRNP family, Hrb27C regulates the transport, localization, and translation of interacting mRNAs during oogenesis (Yano et al, 2004; Goodrich et al, 2004; Huynh et al, 2004). Both Hrb27C and Aub are intrinsic factors required for GSC maintenance (Ma et al, 2017; Rojas-Ríos et al, 2017; Rojas-Ríos et al, 2024; Finger et al, 2023). In this study, we provided evidence supporting the involvement of Hrb27C in Aub-mediated TE silencing in GSCLCs (GSC-like cells induced by depletion of differentiation factor, *bam*) (Fig. 4). The cooperation between Hrb27C and Aub in GSCs warrants further detailed investigation.

H3K4 methyltransferase Set1 was identified and characterized as a key factor in Piwi-mediated TE silencing in the female germline. We initially assumed that, despite the proximity to Piwi (Fig. 2), TE silencing by Set1 could be attributable to its general coactivator function (Ardehali et al, 2011; Mohan et al, 2011). Set1 acting as H3K4me3 writer inside COMPASS potentially underlies the expression of germline genes, including piRNA pathway components (Fig. EV5). However, our results argue against this notion but instead uncover a specialized role for Set1 cooperating with Piwi in piRNA cluster formation at subtelomeric *HTT* repeat loci. First, *set1* and *piwi* but not *aub* showed similar TE derepression pattern upon depletion from germline cells (Fig. 4). Second, *set1* depletion caused severe loss of piRNAs targeting *HTT*, which we found occurring in *panx*-deficient ovaries, disrupting the Piwi pathway (Fig. 6). Moreover, Set1 can repress TEs, *HTT* members in particular, without its catalytic activity (Fig. 5). CUT&Tag revealed the localization of Set1 to TE sequences, including *HeT-A* 3’UTR, possessing antisense TSS. Set1 is enriched at subtelomeric *HTT* repeats, and required for accumulating Rhi, a key activator of piRNA precursor transcription (Figs. 7 and 8).

How Set1 recognizes TE sequences has remained elusive. Set1 interacts with the phosphorylated C-terminal domain (CTD) of RNA polII in the elongation phase, which allows targeted recruitment and H3K4me3 deposition on actively transcribed genes (Ng et al, 2003). In our CUT&Tag analysis, H3K4me3 signals within TE sequences were largely overlapped with Set1-binding regions. Although our results did not support a role for Set1 in depositing these TE-associated H3K4me3 (Fig. 7F), RNA polII on transcriptionally active TEs could contribute to Set1 recruitment. However, *HeT-A* showed only mild derepression by downregulation of a COMPASS component, Wdr82, which is the orthologue of yeast Swd2 linking Set1 and RNA polII (Fig. EV5C) (Bae et al, 2020). Hence, RNA polII-dependent recruitment of Set1 as part of COMPASS may be insufficient, and additional mechanisms may contribute. Our proximity proteome did not identify SetDB1/Eggless, the H3K9me3 writer responsible for heterochromatin formation at Piwi-piRNA target loci (Rangan et al, 2011; Osumi et al, 2019; Akkouche et al, 2017). This absence could be due to the technical limitations of proximity labeling, or it may suggest that our proteome dataset is biased toward a specific stage preceding H3K9me3 deposition. Perhaps related, the ovarian soma-derived culture system demonstrated that Piwi-mediated silencing is initiated co-transcriptionally in an H3K9me3-independent manner. This initial phase requires regulators of RNA Pol II transcription (Wu et al, 2025; Liu et al, 2025; Murano et al, 2019). Further characterization of additional factors identified in Piwi proxiome could provide deeper insight into the mode of action of Set1 in Piwi-mediated piRNA cluster formation and TE silencing in germline cells.

Many piRNAs are derived from defined intergenic regions called piRNA cluster loci (Brennecke et al, 2007). The heterochromatic feature accumulating H3K9me3 requires noncanonical RNA polII recruitment for piRNA precursor transcription, which is mediated by Rhi, a germline-specific HP1 homolog and H3K9me3 reader, and its cofactors, Del and Cuff, forming RDC complexes (Klattenhoff et al, 2009; Mohn et al, 2014; Andersen et al, 2017). Recent studies uncovered cluster-specific molecular underpinnings cooperating with RDC. These include DNA-binding protein, Kipferl, and enhancer of zeste, E(z), the writer of H3K27me3, each required at distinct cluster loci (Akkouche et al, 2025; Baumgartner et al, 2022). In subtelomeres where Kipferl and E(z) appear to have limited involvement (Fig. 4D), Set1 and NSL exert crucial roles (this study and Iyer et al, 2023). Set1 could act suppressively to certain loci, including 38C (Fig. 8). In gonadal soma, a fly orthologue of large Maf transcription factors, Traffic jam (Tj), activates precursor transcription from *flamenco* (Rivera et al, 2025; Alizada et al, 2025). Hence, precursor transcription and piRNA production are governed by diverse factors regulating chromatin and transcription events. Such diversity would enable the spaciotemporal control of piRNA repertoire and PIWI-mediated silencing.

At embryonic stages, Piwi plays key roles in establishing germline piRNA cluster identity through the deposition of H3K9me3 and accumulation of Rhi (Akkouche et al, 2017). Our finding that Set1 is required for Rhi localization at subtelomeric cluster loci raises the possibility of cooperation with Piwi in this process in embryos (Fig. 8C). However, since Set1 was constitutively depleted from the germline in this study, the developmental stage at which cluster-establishing function of Set1 is exerted remains unclear. If Set1 cooperates with Piwi at embryonic stages, it remains uncertain why the physical link between Set1 and Piwi could be identified in adult ovaries (Figs. 2 and 6A). An intriguing possibility is that the germline at the adult stage, including stem cells, retains the ability to modulate the piRNA cluster status at certain loci. In addition to *HTT*, Set1 binds and controls TE families, including *HMS-Beagle*, *3S18*, and *Max-element*, whose fragments are poorly inserted into piRNA cluster loci. Consistent with this observation, full-length insertions remain mobile in the female germline through oocyte targeting (Yang et al, 2023; Wang et al, 2018). The functional significance of Set1 binding to stand-alone TE insertions, particularly within their 3’UTRs, remains unknown and warrants future investigation.

Fly Set1 can silence TEs forming telomeres regardless of its methyltransferase activity (Figs. 5C and 8A). This finding is reminiscent of molecular features reported for yeast Set1, which was originally identified as a transcriptional repressor in the telomere and silent mating loci (Nislow et al, 1997). Notably, its repressive functions on genes and TEs are often associated with H3K4 methylation-independent mechanisms (Jezek et al, 2023; Lee et al, 2018; Lorenz et al, 2012). However, the mechanisms underlying TE silencing are different between the two organisms, as yeast lacks PIWI clade proteins and does not produce piRNAs. Nonetheless, Set1-dependent antisense non-coding RNAs have been characterized as repressors of retrotransposons in yeast (Berretta et al, 2008). The role of Set1 in generating antisense, silencing-competent transcripts for genome defense may be conserved across broader contexts.

## Methods

**Table:** Reagents and tools table

Reagent/resource	Reference or source	Identifier or catalog number
**Experimental models**		
*Drosophila melanogaster*		
*y[1] w[1118]*	Bloomington Drosophila Stock Center (BDSC)	6598
*NGT40-Gal4; nos-Gal4 VP16*	Grieder et al, 2000	
*bam* shRNA y[1] v[1]; P{y[+t7.7] v[+t1.8]=TRiP.HMJ22155}attP40	BDSC	58178
*bam* shRNA y[1] v[1]; P{y[+t7.7] v[+t1.8]=TRiP.HMS00029}attP2	BDSC	33631
*attP-3B* y[1] M{RFP[3xP3.PB] GFP[E.3xP3]=vas-int.Dm}ZH-2A w[*] v[1] P{y[+t7.7]=nanos-phiC31\int.NLS}X; PBac{y[+]-attP-3B}VK00033	BDSC	32543
*attP-40* y[1] v[1] P{y[+t7.7]=nanos-phiC31\int.NLS}X; P{y[+t7.7]=CaryP}Msp300[attP40]	BDSC	25709
*attP-2* P{y[+t7.7]=nanos-phiC31\int.NLS}X, y[1] sc[1] v[1] sev[21]; P{y[+t7.7]=CaryP}attP2	BDSC	25710
*w; {UASp-miniTurbo-GFP}attP40/CyO*	This study	
*w; {UASp-miniTurbo-GFP-Piwi}attP2/TM3 Sb*	This study	
*w; {UASp-miniTurbo-GFP-Aub}attP3B/TM3 Sb*	This study	
*w; {UASp-miniTurbo-GFP-Ago3}attP40/CyO*	This study	
*gfp* shRNA 1 y[1] sc[*] v[1] sev[21]; P{y[+t7.7] v[+t1.8] = VALIUM20-EGFP.RNAi.1}attP40	BDSC	41555
*gfp* shRNA 2 y[1] sc[*] v[1] sev[21]; P{y[+t7.7] v[+t1.8] = VALIUM20-EGFP.RNAi.1}attP2	BDSC	41556
*aub* shRNA y[1] sc[*] v[1] sev[21]; P{y[+t7.7] v[+t1.8]=TRiP.HMS00611}attP2	BDSC	33728
*piwi* shRNA y[1] sc[*] v[1] sev[21]; P{y[+t7.7] v[+t1.8]=TRiP.GL00626}attP40	BDSC	37483
*hrb27C* shRNA y[1] sc[*] v[1] sev[21]; P{y[+t7.7] v[+t1.8]=TRiP.HMS00597}attP2	BDSC	33716
*set1* shRNA y[1] sc[*] v[1] sev[21]; P{y[+t7.7] v[+t1.8]=TRiP.HMS00581}attP2	BDSC	33704
Set1 WT RNAi-resistant transgene UASp-FRT-eGFP-set1 (WT)-PolyA-FRT	Vidaurre et al, 2024	
Set1 E1613K RNAi-resistant transgene UASp-FRT-eGFP-set1 (E1613K)-PolyA-FRT	Vidaurre et al, 2024	
*ash2* shRNA y[1] sc[*] v[1] sev[21]; P{y[+t7.7] v[+t1.8]=TRiP.GL00307}attP2/TM3, Sb[1]	BDSC	35388
*rbbp5* shRNA y[1] v[1]; P{y[+t7.7] v[+t1.8]=TRiP.HMJ02113}attP40	BDSC	42819
*wds* shRNA y[1] sc[*] v[1] sev[21]; P{y[+t7.7] v[+t1.8]=TRiP.HMS00746}attP2	BDSC	32952
*wdr82* shRNA y[1] sc[*] v[1] sev[21]; P{y[+t7.7] v[+t1.8]=TRiP.HMS00718}attP2	BDSC	32926
**Recombinant DNA**		
*3xHA-miniTurboNLS_pCDNA3*	Addgene	107172
*pUASp-K10-attB*	Koch et al, 2009	
*pUASp-mTurbo-eGFP*	This study	
*pUASp-mTurbo-eGFP-Piwi*	This study	
*pUASp-mTurbo-eGFP-Aub*	This study	
*pUASp-mTurbo-eGFP-Ago3*	This study	
**Antibodies**		
Rabbit anti-GFP Living Colors	Clontech	632592
Mouse anti-Piwi antibody	Saito et al, 2006	
Guinea pig anti-Aub antibody	Lim et al, 2022	
Rat anti-Ago3 antibody	Lim et al, 2022	
Mouse anti-Tubulin antibody DM1a	Santa Cruz	sc-32293
Peroxidase AffiniPure Goat anti-guinea pig IgG (H + L)-HRP Conjugate	Jackson Immuno Research	529-33271
Goat anti-Rabbit IgG (H + L)-HRP Conjugate	Bio-Rad	1706515
Goat anti-Mouse IgG (H + L)-HRP Conjugate	Bio-Rad	1721011
Rabbit anti-Rat IgG-HRP Conjugate	Agilent/Dako	P0450
Streptavidin-HRP Conjugate	Proteintech	PF00030
Rabbit anti-HeT-A Gag antibody	Lin et al, 2023	
Guinea pig anti-Krimp antibody	Lim and Kai, 2007	
Mouse anti-Piwi antibody	Santa Cruz	sc-390946
Mouse anti-GFP antibody	Invitrogen	A11120
Goat anti-rabbit Alexa Fluor 488	Thermo Fisher Scientific	A11008
Goat anti-guinea pig Alexa Fluor 555	Thermo Fisher Scientific	A21435
Goat anti-mouse CF633	Biotium	50-196-4311
Mouse anti-H3K4me3 antibody	Active Motif	61979
Rabbit anti-GFP antibody	MBL	598
Rabbit anti-Rhino antibody	Mohn et al, 2014	
Donkey Anti-Mouse IgG (H + L) antibody	Cell Signalling	52885 L
Goat Anti-Rabbit IgG (H + L) antibody	Cell Signalling	35401S
Anti-GFP mAb-Magnetic Beads	MBL	D153-11
**Oligonucleotides and other sequence-based reagents**		
**Primers for construction**		
miniTurbo_linker_Rv_general: GCCGCCGCCTCCGCCGTCGGCCCTGCTGAATTCCT	This study	
2-1vLinker > GFP5_F: GGCGGAGGCGGCGGCATGGTGAGCAAGGGC	This study	
Ti876-GFP-G5-R: GCCACCGCCTCCACCCTTGTACAGCTCGTCCATGCCG	This study	
Ti893-G5-Aub-CDS-F: GGTGGAGGCGGTGGCAATTTACCACCAAACCCTGTAAT	This study	
Ti894-Aub-UASp-R1: CGAGGTCGACTCTAGTTACAAAAAGTACAATTGATTC	This study	
Linker to Piwi: GGTGGAGGCGGTGGCGCTGATGATCAGGGACGTGGAC	This study	
Piwi to Vector: CGAGGTCGACTCTAGTTATAGATAATAAAACTTCTTTT	This study	
Linker to Ago3: GGTGGAGGCGGTGGCTCTGGAAGAGGAAATTTGTTGA	This study	
Ago3 to Vector: CGAGGTCGACTCTAGTTAAAGATAAAATAGTTTTTCAG	This study	
**Primers for RT-qPCR**		
rp49 Fw: CCGCTTCAAGGGACAGTATCTG	This study	
rp49 Rv: ATCTCGCCGCAGTAAACGC	This study	
HeT-A Fw: TCATTGACGATACCAGCGCATC	This study	
HeT-A Rv: TCCGGGTGCGTTTAGGTGAG	This study	
aub_PP35447_Fw: TTACGCCTGATGTGGAGGCTA	This study	
aub_PP35447_Rv: GGGGCTATCTTGAACAGCTTTG	This study	
hrb27C_PD70445_Fw: ACATGCCACCTAACTCTGCC	This study	
hrb27C_PD70445_Rv: TTGAGCACGCGAGTACATGT	This study	
set1 Fw: CAAAAGGCATTGATGCCGAAG	This study	
set1 Rv: GGTCAGTTGTGCAGTGATCCACC	This study	
**Primers for CUT&Tag library construction**		
CUT&Tag_i702: CAAGCAGAAGACGGCATACGAGATCTAGTACGGTCTCGTGGGCTCGGAGATGTG	Epicypher	
CUT&Tag_i703: CAAGCAGAAGACGGCATACGAGATTTCTGCCTGTCTCGTGGGCTCGGAGATGTG	Epicypher	
CUT&Tag_i704: CAAGCAGAAGACGGCATACGAGATGCTCAGGAGTCTCGTGGGCTCGGAGATGTG	Epicypher	
CUT&Tag_i705: CAAGCAGAAGACGGCATACGAGATAGGAGTCCGTCTCGTGGGCTCGGAGATGTG	Epicypher	
CUT&Tag_i706: CAAGCAGAAGACGGCATACGAGATCATGCCTAGTCTCGTGGGCTCGGAGATGTG	Epicypher	
CUT&Tag_i707: CAAGCAGAAGACGGCATACGAGATGTAGAGAGGTCTCGTGGGCTCGGAGATGTG	Epicypher	
CUT&Tag_i708: CAAGCAGAAGACGGCATACGAGATCCTCTCTGGTCTCGTGGGCTCGGAGATGTG	Epicypher	
CUT&Tag_i501: AATGATACGGCGACCACCGAGATCTACACTAGATCGCTCGTCGGCAGCGTCAGATGTGTAT	Epicypher	
CUT&Tag_i502: AATGATACGGCGACCACCGAGATCTACACCTCTCTATTCGTCGGCAGCGTCAGATGTGTAT	Epicypher	
CUT&Tag_i503: AATGATACGGCGACCACCGAGATCTACACTATCCTCTTCGTCGGCAGCGTCAGATGTGTAT	Epicypher	
CUT&Tag_i504: AATGATACGGCGACCACCGAGATCTACACAGAGTAGATCGTCGGCAGCGTCAGATGTGTAT	Epicypher	
CUT&Tag_i505: AATGATACGGCGACCACCGAGATCTACACGTAAGGAGTCGTCGGCAGCGTCAGATGTGTAT	Epicypher	
CUT&Tag_i506: AATGATACGGCGACCACCGAGATCTACACACTGCATATCGTCGGCAGCGTCAGATGTGTAT	Epicypher	
CUT&Tag_i507: AATGATACGGCGACCACCGAGATCTACACAAGGAGTATCGTCGGCAGCGTCAGATGTGTAT	Epicypher	
**Chemicals, enzymes, and other reagents**		
D-Biotin (Vitamin H)	Nacalai	04822-91
cOmplete Protease Inhibitor Cocktail Tablet	Roche	11873580001
Dynabeads MyOne Streptavidin C1	Thermo Fisher Scientific	65001
Triton X-100	Wako	168-11805
Nonidet P-40 (NP-40) substitute	Nacalai	18551-24
Dithiothreitol (DTT)	Nacalai	14112-94
Trypsin Gold, Mass Spectrometry Grade	Promega	V5280
ZipTip C18	Millipore	ZTC18M008
Chemi-Lumi One L	Nacalai	07880
PVDF membrane 0.2 μm	Wako	033-22453
Skim milk	Nacalai	31149-75
TRIzol LS	Invitrogen	10296010
DNase I	NEB	M0303S
SuperScript III Reverse Transcriptase	Thermo Fisher Scientific	18080-085
SYBR Green qPCR Master Mix	Thermo Fisher Scientific	A46012
RNA Clean & Concentrator 5	Zymo Research	R1015
RNase H	NEB	M0297S
NEBNext Multiplex Small RNA Library Prep Set for Illumina	NEB	E7300S
MagMAX Pure Bind (library purification)	Applied Biosystems	A58521
Low-melting agarose	HydraGene	BC-R9012LM
SYBR Gold nucleic acid gel stain	Thermo Fisher Scientific	S11494
QIAquick Gel Extraction Kit	Qiagen	28704
Glycogen	Nacalai	07592-21
Dynamarker DM253	BioDynamics Laboratory Inc.	DM253
DNA LoBind tubes 0.5 ml	Eppendorf	022431005
DNA LoBind tubes 1.5 ml	Eppendorf	022431021
DNA LoBind tubes 2.0 ml	Eppendorf	022431048
Cellulose 0.22 μm membrane filter Spin-X	Costar	8160
In-Fusion HD Cloning Kit	Takara	ST2312
Paraformaldehyde (crosslinking IP)	ElectronMicroscopy Sciences	15710
Bovine Serum Albumin	Wako	015-28393
Image-iT FX Signal Enhancer	Invitrogen	I36933
DAPI	Nacalai	19178-91
Spermidine	Nacalai	32110-41
pAG-Tn5	Cell Signalling	79561S
Collagenase	Sigma	C9407
Proteinase K	Nacalai	29442-14
Quick-DNA Microprep Kit	Zymo Research	D3020
Ultra II Q5 Master Mix	NEB	M0544S
AmpureXP	Beckman	A63881
Qubit dsDNA HS Assay Kit	Thermo Fisher Scientific	Q32851
**Software**		
Fiji (ImageJ)	https://imagej.net/software/fiji/	2.14.0/1.54f
ZEISS ZEN	Zeiss	v3.0
Proteome Discoverer	Thermo Fisher Scientific	v2.5
CytoScape	Ono et al, 2025	https://cytoscape.org/index.html
MetaScape	Zhou et al, 2019	https://metascape.org/
Cutadapt	Martin, 2011	v1.18
STAR	Dobin et al, 2013	v2.7.10b
Bowtie	Langmead et al, 2009	v1.3.1
Bowtie2	Langmead and Salzberg, 2012	v2.5.1
featureCounts	Liao et al, 2014	v2.1.1
pileup.sh / splitsam.sh / BBMap suite	Bushnell et al, 2017	v39.01
deeptools	Ramírez et al, 2016	v3.5.6
wiggletools	Zerbino et al, 2014	v1.2.12
wigToBigWig	UCLC facility	v2.9
IGV	https://igv.org/	v2.16.1
seqkit	Shen et al, 2016	v2.4.0
samtools	Li et al, 2009	v1.3.1
bedtools	Quinlan and Hall, 2010	v2.26.0
signature.py	Antoniewski, 2014	
Trim Galore	Krueger, Trim Galore	v0.6.2
BLAST+	Camacho et al, 2009	v2.16.0+
**Other**		
Bioruptor	Diagenode	
Trans-Blot Turbo	Bio-Rad	
ChemiDoc MP Imaging System	Bio-Rad	
Orbitrap Eclipse Tribrid Mass Spectrometer with FAIMS Pro interface	Thermo Fisher Scientific	
Vanquish Neo UHPLC system	Thermo Fisher Scientific	
ZEISS LSM 900 confocal microscope	Zeiss	
C-Apochromat 40x/1.20 W Korr objective lens	Zeiss	
QuantStudio 5 Real-Time PCR System	Applied Biosystems	
NovaSeq 6000 (RNA-seq sequencing)	Illumina	
NovaSeq X Plus (small RNA-seq; RIMD NGS core facility, Univ. of Osaka)	Illumina	
NovaSeq X (CUT&Tag sequencing; Rhelixa and RIMD)	Illumina	
23 G x 1 needle	TERUMO	

### Fly stocks and culture

All stocks and crosses were raised at 25 °C on a molasses/yeast medium [5% (w/v) dry yeast, 5% (w/v) corn flour, 2% (w/v) rice bran, 10% (w/v) glucose, 0.7% (w/v) agar, 0.2% (v/v) propionic acid, and 0.05% (v/v) p-hydroxy butyl benzoic acid]. Fly stocks used in this study are listed in Table EV3.

### Plasmid construction and generation of transgenic flies

All the primers used for plasmid construction are listed in Table EV4. To generate UASp-mini(m)Turbo-GFP, UASp-mTurbo-GFP-Piwi, UASp-mTurbo-GFP-Aub, and UASp-mTurbo-GFP-Ago3, mTurbo fragment was amplified by PCR using attB_miniTurbo_Fw_general and miniTurbo_linker_Rv_general as primers and 3xHA-miniTurboNLS_pCDNA3 (addgene #107172) as template. GFP fragment was amplified by PCR using 2-1vLinker > GFP5_F and Ti876-GFP-G5-R as primers. Piwi, Aub, or Ago3 fragment was amplified by PCR using primer pairs of Linker_to_Piwi and Piwi_to_Vector, Ti893-G5-Aub-CDS-F and Ti894-Aub-UASp-R1, or Linker_to_Ago3 and Ago3_to_Vector, respectively, with cDNA from *yw* ovaries as template. PCR fragments were introduced into the XbaI site in pUASp-K10-attB vector (Koch et al, 2009), using In-Fusion HD Cloning (Takara). UASp-mTurbo-GFP and UASp-mTurbo-GFP-Ago3 were integrated to attP40, while UASp-mTurbo-GFP-Piwi and UASp-mTurbo-GFP-Aub were integrated to attP2, and attP-3B sites, respectively.

### Proximity-dependent biotin pulldown in ovarian germline cells

After eclosion, females expressing mTurbo-GFP-PIWI proteins were reared at 25 °C for 2–3 (non-GSCLC ovaries) or 5–7 days (GSCLC ovaries) in the modified molasses/yeast medium supplemented with 100 μM biotin (Nacalai). For individual conditions, ~300 ovaries from 150 progenies were collected. Ovaries were homogenized in 150 μL of PI-lysis buffer [50 mM Tris-HCl (pH 7.5), 500 mM NaCl, 2 mM EDTA, 2 mM dithiothreitol (DTT), 0.4% (w/v) SDS, and cOmplete Protease Inhibitor Cocktail Tablet (Roche)], using Bioruptor (Diagenode) for 30 s (power H) for six times with 30 s intervals. Triton X-100 was then added to samples at a final concentration of 2% (v/v), and homogenization was further performed for 30 s three times with 30 s intervals. After centrifugation (20,000× *g*, 10 min, 4 °C), the supernatant was diluted with an equal amount of 50 mM Tris-HCl (pH 7.5) buffer and incubated with pre-equilibrated 15-μL slurry volume of Dynabeads MyOne Streptavidin C1 (Thermo Fisher Scientific), overnight at 4 °C with gentle rotation. Next day, beads were washed twice with W1 buffer [50 mM Tris-HCl (pH 7.5), 250 mM NaCl, 0.2% (w/v) SDS, 1 mM EDTA, and 1 mM DTT], twice with W2 buffer [50 mM HEPES-KOH (pH 7.4), 500 mM NaCl, 0.1% (w/v) deoxycholate (Nacalai), 1% (v/v) Triton X-100, and 1 mM EDTA], twice with W3 buffer [10 mM Tris-HCl (pH 8.0), 250 mM LiCl, 0.5% (w/v) deoxycholate, 0.5% (v/v) NP-40 (Nacalai), and 1 mM EDTA], and four times with W4 buffer [50 mM Tris-HCl (pH 7.5) and 50 mM NaCl]. The purified proteins bound to beads were stored at −80 °C until analysis.

### Immunoblotting and biotinylated protein detection

Protein samples were denatured at 95 °C for 5 min in 2× protein loading buffer [4% (w/v) SDS, 200 mM DTT, 0.1% (v/v) bromophenol blue (BPB), and 20% (v/v) glycerol] saturated with biotin, resolved by SDS–polyacrylamide gel electrophoresis (SDS-PAGE) and transferred to 0.2-μm polyvinylidene difluoride membrane (Wako) using the semi-dry system (Trans-blot Turbo, Bio-Rad). The membrane was blocked in 4% (w/v) skim milk (Nacalai) in 1× phosphate-buffered saline (PBS) supplemented with 0.1% (v/v) Tween 20 and further incubated with primary antibodies: rabbit anti-GFP (1:1000, Clonetech), mouse anti-Piwi (1:100) (Saito et al, 2006), guinea pig anti-Aub (1:1000), and rat anti-Ago3 (1:200) (Lim et al, 2022), mouse anti-Tubulin (1:3000, Santa Cruz). Secondary antibodies were anti-guinea pig immunoglobulins-HRP (1:1000; Dako), anti-rabbit immunoglobulin G (IgG)–HRP (1:3000; Bio-Rad), anti-mouse IgG-HRP (1:3000; Bio-Rad), and anti-rat IgG (1:1000; DAKO). HRP-conjugated Streptavidin (Proteintech) was used for detecting biotinylated Proteins. The chemiluminescent signals generated with Chemi-Lumi One (Nacalai) were detected by the Chemidoc MP Imaging system (Bio-Rad). The images were processed with Fiji.

### Proteomic sample preparation and label-free quantification using Orbitrap Eclipse Tribrid Mass Spectrometer

Biotinylated proteins bound to magnetic beads were reduced with 1 mM DTT and alkylated with 5.5 mM iodoacetamide. Proteins were digested overnight at 37 °C with Trypsin Gold, Mass Spectrometry Grade (Promega, Madison, WI). The resulting peptides were captured and desalted using ZipTip C18 (Millipore, Billerica, MA). Shotgun proteomic analysis was performed using an Orbitrap Eclipse Tribrid mass spectrometer equipped with a FAIMS Pro interface (Thermo Fisher Scientific, Waltham, MA), which was coupled to a Vanquish Neo UHPLC system (Thermo Fisher Scientific, Waltham, MA). Peptides were separated on a reversed-phase column using a linear gradient of 2–24% acetonitrile in 0.1% formic acid at a flow rate of 300 nL/min. Full MS scans were acquired in the Orbitrap at a resolution of 120,000, followed by MS/MS scans in the ion trap using higher-energy collisional dissociation (HCD) with a normalized collision energy of 35% and a maximum injection time of 10 ms. Label-free quantification (LFQ) was performed using Proteome Discoverer version 2.5 (Thermo Fisher Scientific, Waltham, MA) with the Sequest HT search engine. MS/MS spectra were searched against the UniProt Drosophila melanogaster reference proteome (UP000000803). Protein identifications were filtered at a false discovery rate (FDR) of <1%. Peptide intensities were extracted using the Minora Feature Detector node and used for protein quantification. GO analysis was conducted using MetaScape (Zhou et al, 2019).

### Croslinking and immunoprecipitation

Ovaries were dissected from 50 females expressing GFP, GFP-Set1^WT^, or GFP-Set1^E1613K^ in phosphate-buffered saline (PBS) and immediately fixed with 0.1% (w/v) paraformaldehyde (Electron Microscopy Sciences) for 10 min at room temperature (RT). Fixation was quenched with 125 mM glycine. Samples were then snap-frozen in liquid nitrogen and stored at −80 °C until use. Ovary samples were homogenized with a pestle in lysis buffer [20 mM Tris-HCl (pH 7.5), 135 mM NaCl, 1.5 mM MgCl_2_, 10% (v/v) glycerol, 0.2% (v/v) Triton X-100] supplemented with cOmplete Protease Inhibitor Cocktail (Roche). Pre-equilibrated anti-GFP antibody-conjugated magnetic beads (MBL, D153-11) were added to ovarian lysate and incubated for 1 h at 4 °C. After three times of washing with lysis buffer, bead-bound proteins were extracted by boiling in SDS-PAGE loading buffer [50 mM Tris–HCl (pH 6.8), 2% (w/v) SDS, 1 mM DTT, 10% (v/v) glycerol, 0.05%(w/v) bromophenol blue].

### Histochemistry and image acquisition

Ovaries were dissected from adult females in 1× PBS buffer supplemented with 0.4% (w/v) bovine serum albumin (BSA; Wako) and fixed in 5.3% (v/v) paraformaldehyde (Nacalai) in 0.67× PBS buffer for 10 min. To observe DNA, ovaries were incubated with 1 μM 40,6-diamidino-2-phenylindole (DAPI) in PBX buffer [1x PBS containing 0.2% (v/v) Triton X-100]. For immunostaining, fixed ovaries were washed with PBX and incubated with Image-iT™ FX Signal Enhancer (Invitrogen) for 30 min and PBX containing 2% (w/v) BSA for 30 min for blocking. The primary antibody incubation was performed overnight at 4 °C, and ovaries were washed with PBX at RT for 1 h. The secondary antibody incubation was then performed at RT for 2 h, and then the ovaries were washed with PBX at RT for 1 h. The antibodies used for immunostaining were rabbit anti-HeT-A Gag (1:2000) (Lin et al, 2023), guinea pig anti-Krimp (1:2000) (Lim and Kai, 2007), mouse anti-Piwi (1:50, Santa Cruz, sc-390946), guinea pig anti-Aub (1:500) (Lim et al, 2022), and mouse anti-GFP (1:50, Invitrogen, A11120). Secondary antibodies were as follows: Alexa Fluor 488- and 555-conjugated goat antibodies at 1:500 (Molecular Probes) and CF®633 goat antibodies at 1:500 (Biotium). Antibodies were diluted in 0.4% (w/v) BSA containing PBX as the working solution. Images were taken by ZEISS LSM 900 using C-Apochromat 40×/1.20 W Korr objective lens and processed with ZEISS ZEN 3.0 and Fiji.

### Reverse transcription and qPCR analysis

Total RNA was extracted from ovaries using TRIzol™ LS (Invitrogen) following the manufacturer’s instructions. Using DNase I (NEB)–treated RNA, cDNA was synthesized with 2.5 μM oligo(dT) adaptor using SuperScript III reverse transcriptase (Thermo Fisher Scientific). Quantitative reverse transcription PCR (qPCR) reaction was performed using SYBR™ Green qPCR Master Mix (Thermo Fisher Scientific) and gene-specific primers (Table EV4) in QuantStudio 5 Real-Time PCR system (ABI).

### Transcriptome analysis

Total RNA was extracted from 100 ovaries (non-GSCLC ovaries or GSCLC ovaries) using TRIzol™ LS (Invitrogen). To obtain GSCLC ovaries expressing shRNA for *aub*, *hrb27C*, *set1*, or *gfp*, shRNA lines were combined (*bam* shRNA; *aub* shRNA, *bam* shRNA; *hrb27C* shRNA, *bam* shRNA; *set1* shRNA, *bam* shRNA; *gfp* shRNA) and crossed with NN. DNase I-treated RNA was sent to Rhelixa (Japan) for library construction and deep-sequencing. Polyadenylated RNA was selected with NEBNext Poly(A) mRNA Magnetic Isolation Module (NEB). Libraries were constructed with NEBNext Ultra II Directional RNA Library Prep Kit (NEB), sequenced with NovaSeq 6000 (Illumina). Trimming was performed by Cutadapt (v1.18, -j 12 -a AGATCGGAAGAGCACACGTCTGAACTCCAGTCA -A AGATCGGAAGAGCGTCGTGTAGGGAAAGAGTGT, -m 20). Trimmed paired-end reads were mapped to the genome (BDGP6.46, dm6) using STAR (v2.7.10b, --outFilterMultimapNmax 100 --outSAMmultNmax 1 --outMultimapperOrder Random). Read counting was performed with featureCounts (-M -p --countReadPairs -t exon -g gene_id) using Drosophila_melanogaster.BDGP6.46.112.gtf (ensembl.org). Using the sum of RPK (reads per kilobase) for individual genes and TEs, TPM (transcripts per million) was calculated. Counts on exons were used as input for differential expression analysis by EdgeR (v.4.8.2). Lowly expressed genes were filtered out using filterByExpr function before normalization and statistics. Trimmed paired-end reads were also mapped to consensus sequences of individual TE families (transposon_sequence_set.embl.v.9.41, flybase) with STAR using the same options. Mappers on individual TE families were counted using BBMap (pileup.sh). TPM was calculated using the sum of RPK for genes and TEs obtained in genome mapping. Public data (GSE103582, GSE71374, GSE130042, GSE126578, GSE202468, GSE247156) were processed in the same way.

### Small RNA sequencing and analysis

Biological duplicate dataset for Set1-present condition was provided by *gfp-GLKD* ovaries (control RNAi condition), and *set1*-GLKD ovaries expressing GFP-Set1^WT^ (rescue condition). The dataset for the Set1-depleted condition was provided by *set1*-GLKD ovaries and the siblings of the rescue conditions (CyO). Total RNA was extracted using TRIzol™ LS (Invitrogen) from 60 to 100 ovaries of ~3-day-old adult females. After the addition of chloroform and centrifugation (12,000 ×  *g* 15 min, 4 °C), short (< ~200 nt) RNA in the aqueous phase was purified using RNA Clean & Concentrator 5 (R1015 Zymo). To deplete 2S ribosomal RNA (rRNA), 2 pmol of complementary oligo DNA (5’-AGTCTTACAACCCTCAACCATATGTAGTCCAAGCAGCACT-3’) was added per 1 μg of RNA. The mixture was heated (95 °C, 2 min), gradually cooled down to form a DNA/RNA hybrid, and then treated with RNase H (NEB) at 37 °C for 30 min. RNase H was inactivated by heating at 65 °C for 20 min. The 2S rRNA-depleted RNA mixture was loaded onto 8 M urea-polyacrylamide gel (12%) and size-separated in parallel with a visible RNA ladder (Dynamarker DM253, BioDynamics Laboratory Inc.) in 0.5× Tris-borate EDTA buffer. Gel within the range from 20 to 30 nt was excised and passed through a small hole opened by a needle (23Gx1, TERUMO) at the bottom of 0.5 mL DNA LoBind tubes (Eppendorf) by centrifugation (15,000 × *g*). The fine particles were recovered in 2.0 mL DNA LoBind tubes, and RNAs were eluted overnight at 4 °C with gentle rotation in the presence of 300 mM NaOAc (pH 5.2). After removing the particles using a cellulose 0.22-µm membrane filter Spin-X (Costar, 8160), RNA was precipitated in the presence of 80% (v/v) ethanol and glycogen (40 μg/mL) (Nacalai) overnight at −20 °C. After centrifugation (20,000 × *g*, 20 min, 4 °C), the RNA pellet, rinsed twice with 80% (v/v) ethanol, was resuspended in RNase-free water. Small RNA libraries were prepared using NEBNext® Multiplex Small RNA Library Prep Set for Illumina (NEB, E7300S) following the manufacturer’s procedure. After 15 cycles of PCR amplification, the libraries were purified using MagMAX™ Pure Bind Beads (Applied Biosystems), size-separated in 3% (w/v) low-melting agarose (HydraGene) in the presence of SYBR gold (Thermo Fisher). ~150-bp library fragments containing 20–30-bp inserts were purified using the QIAquick gel extraction kit (Qiagen). Libraries were sequenced by the RIMD NGS core facility (The University of Osaka) using the NovaSeqX Plus platform (Illumina). Adaptor sequences (AGATCGGAAGAGC) were removed by Trim Galore (v0.6.2, --length 15) with quality filter cut off (Phred ≥20). Adaptor-trimmed reads were mapped to rRNA, tRNA, snoRNA, and snRNA sequences using bowtie (v1.3.1), and the unmappers were collected (samtools view -f 4). From the unmappers, 18–29-nt reads were selected as small RNAs using seqkit (v2.4.0) and mapped to miRNA precursors using bowtie (-v 0) to collect “miRNA” populations. The size-selected (23–29-nt) unmappers were considered as “piRNA”. These piRNA fragments were mapped to TE consensus sequences (transposon_sequence_set.embl.v.9.41, flybase) using bowtie, allowing up to 3 mismatches and taking one from multi mappers (-v 3 -M 1 --best --strata). Fw and Rv mappers were counted for individual TEs using pileup.sh. Read counts were normalized using the sum of 18–29-nt reads, and reads per million (RPM) were obtained. For visualization, Fw and Rv mappers were separated using splitsam.sh. Bedgraph was generated using bedtools (v2.26.0, genomecov -bga -split), values normalized using scale factors given by the sum of 18 ~ 29-nt reads. Mean values of biological replicates were obtained using bedtools (unionbedg). Track view was generated in IGV (v2.16.0). piRNA overlap scores (z) were measured using signature.py (Antoniewski, 2014). piRNA fragments were also mapped to Drosophila genome (BDGP6.46, dm6) using bowtie without allowing mismatch (-v 0 -M 1 --best --strata). Bedgraph generation, normalization, averaging, and visualization follow the processes in the analysis using TE consensus sequences. Public data (GSE71374, GSE126578, GSE55824) were processed in the same way, except for the removal of 4 nt from both 5’ and 3’ ends in the trimming process for GSE126578 and GSE55824.

### CUT&Tag sequencing and analysis

We referred (Anderson et al, 2023) for performing whole ovary CUT&Tag. Twenty ovaries from 10 adult females at ~3 days old were dissected in PBS buffer supplemented with cOmplete Protease Inhibitor (Roche). Ovaries were permeabilized in PBX buffer (PBX containing 0.2%(v/v) Triton-X100) for 30 min at RT. After removing PBX, ovaries were washed once with Wash+ buffer (20 mM HEPES [pH 7.4], 150 mM NaCl, 0.5 mM spermidine [Nacalai], 2 mM EDTA, 1%[w/v] BSA, cOmplete Protease Inhibitor) and incubated overnight at 4 °C with primary antibodies diluted by 1:50 with Wash+ buffer. Anti-H3K4me3 (#61979, mouse, Active Motif), anti-GFP (#598, rabbit, MBL), or anti-Rhino (rabbit) (Mohn et al, 2014) antibodies were used. The next day, the ovaries were washed three times with Wash+ buffer and incubated with secondary antibodies diluted 1:50 with Wash+ buffer. Anti-mouse IgG (#52885 L, Cell Signalling), or anti-rabbit IgG (#35401S, Cell Signalling) were used. After removing the secondary antibody solution, ovaries were washed three times with 300Wash+ buffer (20 mM HEPES [pH 7.4], 300 mM NaCl, 0.5 mM spermidine, cOmplete Protease Inhibitor) and incubated for 1 h at RT with loaded pAG-Tn5 (#79561S, Cell Signalling) diluted by 1:25 with 300Wash+ buffer. After removing the Tn5 solution, ovaries were washed three times with 300Wash+ buffer. Tagmentation was performed by incubating ovaries in 300Wash+ buffer supplemented with 10 mM MgCl_2_ for 1 h at 37 °C. After removing the supernatant, ovaries were treated with collagenase (2 mg/mL, Sigma C9407) in HEPESCA buffer (50 mM HEPES [pH 7.4], 360 µM CaCl_2_) for 1 h at 37 °C. Then, the reaction mixture was added with SDS, EDTA, and proteinase K (Nacalai) (final concentration 0.2%[w/v], 16 mM, and 0.3 mg/mL, respectively), and further incubated for 1 h at 58 °C. After the reaction, DNA was purified by using Quick-DNA Microprep kit (D3020, ZYMO). Using the purified DNA as template, libraries were synthesized by PCR (72 °C 5 min, 98 °C 30 s, 14 cycles of [98 °C 10 s, 63 °C 15 s], 65 °C 5 min) using Ultra II Q5 Master Mix (NEB) and dual-indexed primers (Table EV4). Generated libraries were purified using ×1.3 volume of AmpureXP (Beckman). The concentration of libraries was measured using Qubit dsDNA HS assay kit (Thermo). Libraries were sequenced with NovaSeqX (Illumina) and 150 + 150 bp paired-end reads were obtained (H3K4me3 libraries sequenced by RIMD in the University of Osaka, GFP libraries sequenced by Rhelixa). Adaptor trimming was performed by Cutadapt (-a CTGTCTCTTATACACATCTCCGAGCCCACGAGAC -A CTGTCTCTTATACACATCTGACGCTGCCGACGA, -m 20). Trimmed reads were mapped to *D. melanogaster* genome (BDGP6.46, dm6) using Bowtie2 (-p 12 --no-unal --end-to-end --very-sensitive --no-mixed --no-discordant -q --phred33 -I 10 -X 700). Normalization of read counts was performed using deeptools (bamCoverage --normalizeUsing CPM -of bigwig --binSize=1). CPM-normalized bigWig files from biological replicates were averaged using wiggletools mean, and converted to bigWig format using wigToBigWig. Track view was generated in IGV (v2.16.0). Trimmed reads were also mapped to TE consensus sequences (transposon_sequence_set.embl.v.9.41, flybase). Normalization was performed using deeptools (bamCoverage --scalefactor -of bigwig --binSize=1). Scale factors were given by the total number of genome mappers. Plot profile and heatmap were generated using deeptools (computeMatrix scale-regions --regionBodyLength 5000 --skipZeros, then, plotHeatmap or plotProfile). For anti-GFP CUT&Tag, log2FC of mean CPM adding pseudocount 1 was calculated by bigwigCompare, and used as input for computeMatrix. Peak calling for GFP-Set1 was performed by MACS2 (-p 1e-2) using GFP as background signals. Peak-associated genes and TEs were identified by bedtools closest. Overlap between biological duplicates was analyzed by Venn Diagram in R. CUT&Tag library reads were counted with featureCounts (v2.1.1, -M -p --countReadPairs -O). For anti-H3K4me3 libraries, mapping reads around gene TSS ( ± 500 bp) were counted with dmel-all-r6.32.gtf (Flybase). For anti-GFP libraries, mappers on exons of genes and TEs were counted using Drosophila_melanogaster.BDGP6.46.112.gtf (Ensembl). For normalization, RPM (reads per million) was calculated by the total number of genome mappers.

### ChIP-seq data re-mining

Raw fastq files (GSE83238) were mapped to the dm6 genome using Bowtie (-v 0 -M 1 --best --strata). IP and input signals were compared using deeptools (bamCompare --operation log2). bigWig files from biological replicates were averaged using wiggletools mean, and converted to bigWig format using wigToBigWig. Track view was generated in IGV (v2.16.0).

### Identification of TE insertions

TE insertions were identified by BLAST+ (v2.16.0 + ) (-outfmt 6 -evalue 1e-10 -perc_identity 70 for *invader4* and *HeT-A*, 90 for *TAHRE*) using *D. melanogaster* genome (dm6) as a reference and consensus TE sequences as queries. Output blast6 files were converted to gff3 format using an awk script.

## Supplementary information

Table EV1

Table EV2

Table EV3

Table EV4

Peer Review File

Dataset EV1

Dataset EV2

Source data Fig. 1

Source data Fig. 2

Source data Fig. 3

Source data Fig. 4

Source data Fig. 5

Source data Fig. 6

Source data Fig. 7

Source data Fig. 8

Expanded View Figures

## Data Availability

Newly generated data are available with the BioProject accession ID. PRJNA1345876: *Drosophila* ovary polyA transcriptome sequencing, PRJNA1345540: *Drosophila* ovary small RNA sequencing, PRJNA1480953: *Drosophila* ovary CUT&Tag sequencing. The source data of this paper are collected in the following database record: biostudies:S-SCDT-10_1038-S44319-026-00901-6.
