# Supplementary material for: PIWI proximity proteome reveals Set1-mediated piRNA biogenesis for transposon silencing at telomeres
Source: EMBO Rep. 2026 Aug 26;27(19):5490–529. doi: 10.1038/s44319-026-00901-6 (PMC13650161; doi:10.1038/s44319-026-00901-6)
Supplement: Supplementary file 1 [file 44319_2026_901_MOESM16_ESM.pdf]

Expanded View Figures

Figure EV1. Germline PIWI proximity biotin labeling and purification.

(**A**) Comparison of efficiency for biotinylation and biotinylated protein purification between non-GSCLC (germline stem cell-like cell) and GSCLC ovaries. Coomassie brilliant blue (CBB) staining serves as a protein loading control. Ovaries expressing mTurbo-GFP or mTurbo-GFP-Aub were analyzed in parallel with a negative control condition (without mTurbo protein expression). Asterisks: non-specific signals. (**B**) Schematic illustration for germline differentiation and the function of Bam. Left: germarium in wild-type, non-GSCLC ovaries. A germline stem cell (GSC, blue) divides to produce a daughter cell (red), which expresses Bam and starts the differentiation. Two to three GSCs are normally maintained in a germarium. Right: by *bam*-GLKD (germline knockdown), daughter cells cannot differentiate and thus accumulate as GSCLCs. (**C**) A pair of wild-type ovaries (*y w*, left) and that of *bam*-GLKD ovaries (right). Scale bar = 1 mm. (**D**) Immunoblotting images comparing the expression level of mTurbo-GFP-tagged Piwi or Aub with that of endogenous Piwi or Aub in GSCLC ovaries.

© The Author(s)

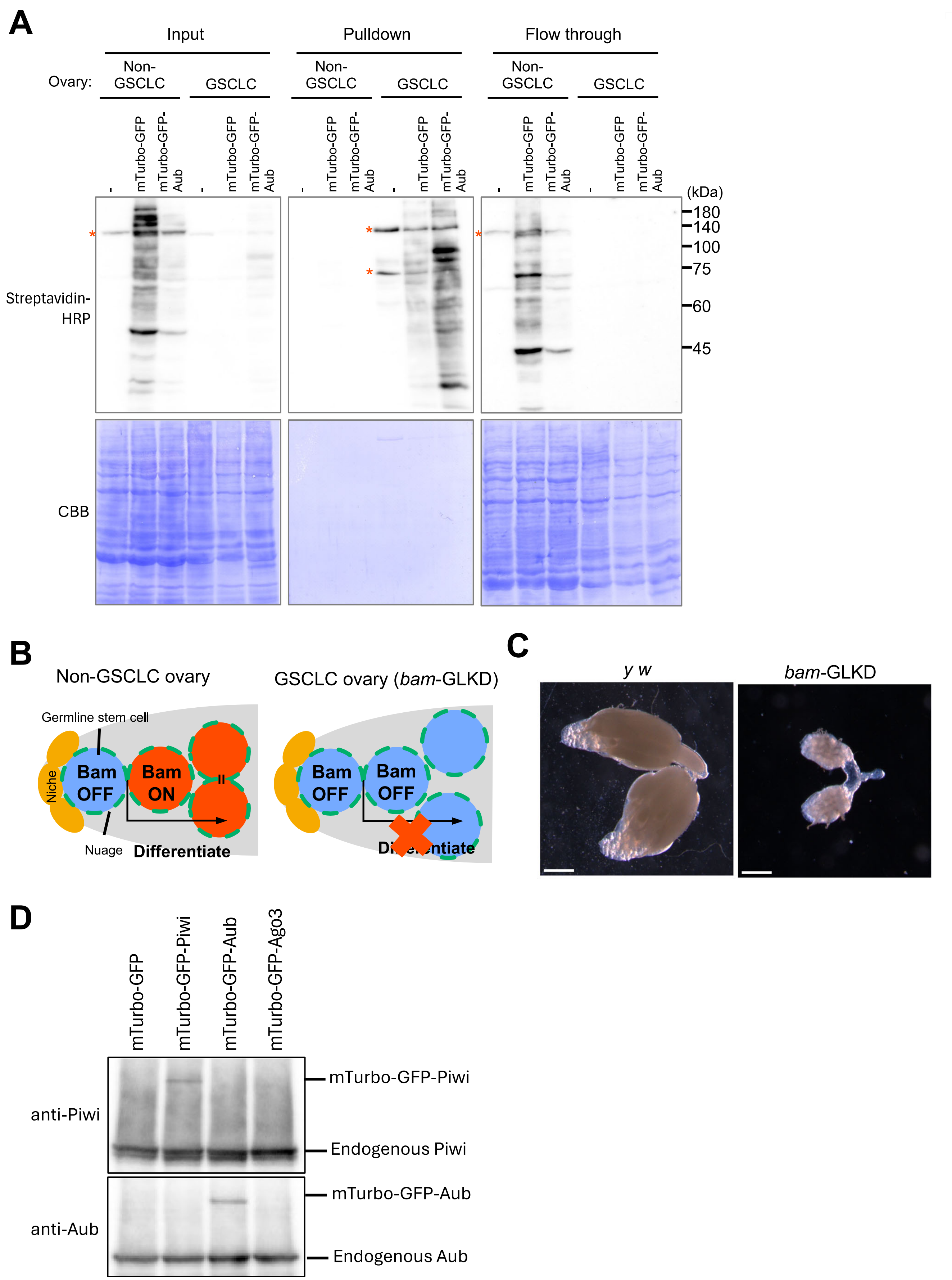
A
Input
Pulldown
Flow through
Ovary:
Non-GSCLC
GSCLC
-
mTurbo-GFP
mTurbo-GFP-Aub
(kDa)
180
140
100
75
60
45
Streptavidin-HRP
CBB
B
Non-GSCLC ovary
GSCLC ovary (bam-GLKD)
Germline stem cell
Niche
Bam OFF
Bam ON
Nuage
Differentiate
C
y w
bam-GLKD
D
mTurbo-GFP
mTurbo-GFP-Piwi
mTurbo-GFP-Aub
mTurbo-GFP-Ago3
anti-Piwi
anti-Aub
mTurbo-GFP-Piwi
Endogenous Piwi
mTurbo-GFP-Aub
Endogenous Aub

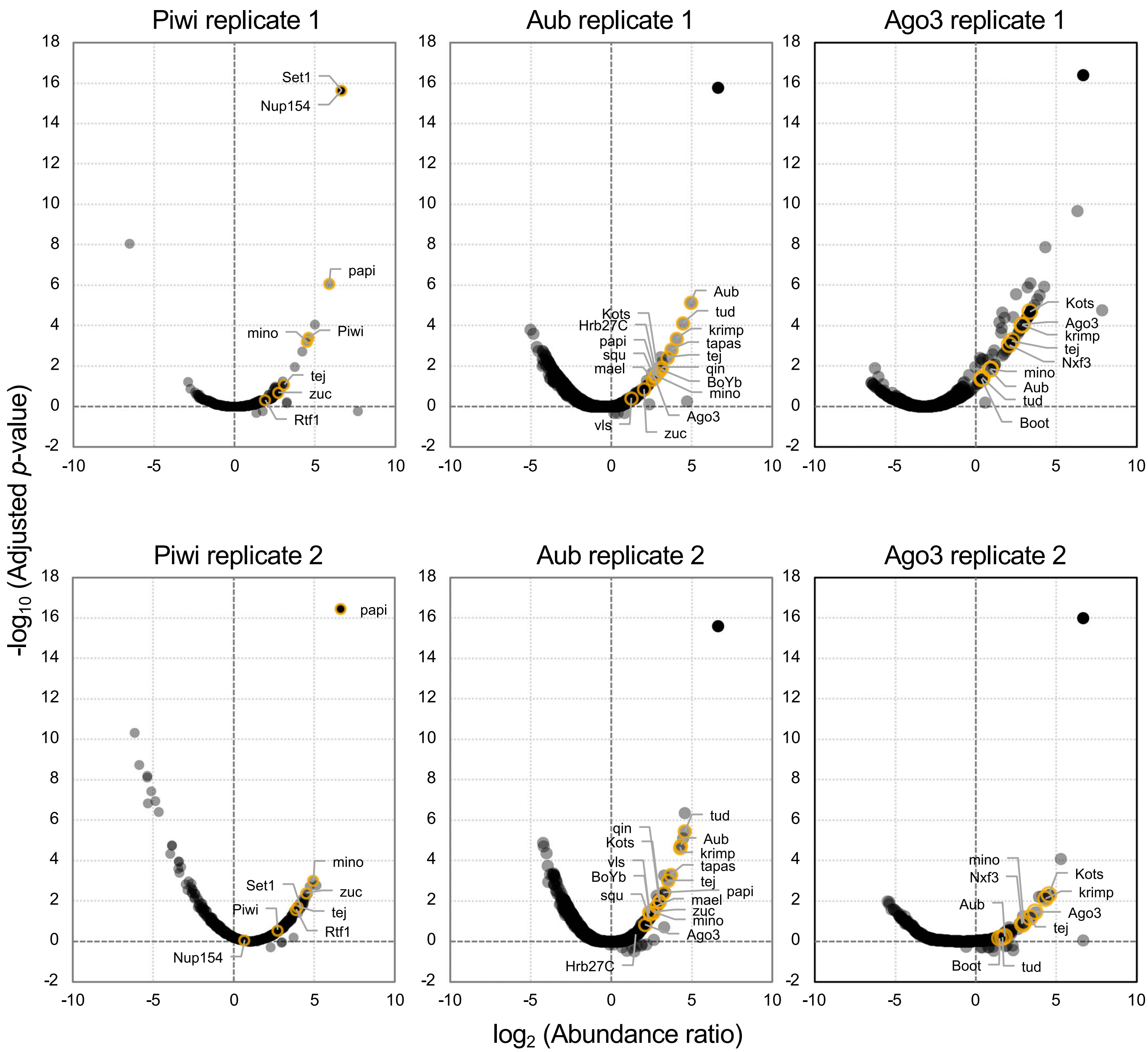

Figure EV2. Germline PIWI proximity proteome in biological duplicates.

Volcano plots for the proximity factors of Piwi, Aub, or Ago3 in individual biological replicates ($n = 2$). Abundance ratio: GFP-PIWI/GFP. Adjusted p is based on a background-based t test. Factors known to be involved in the piRNA pathway are indicated and highlighted with yellow circles.

© The Author(s)

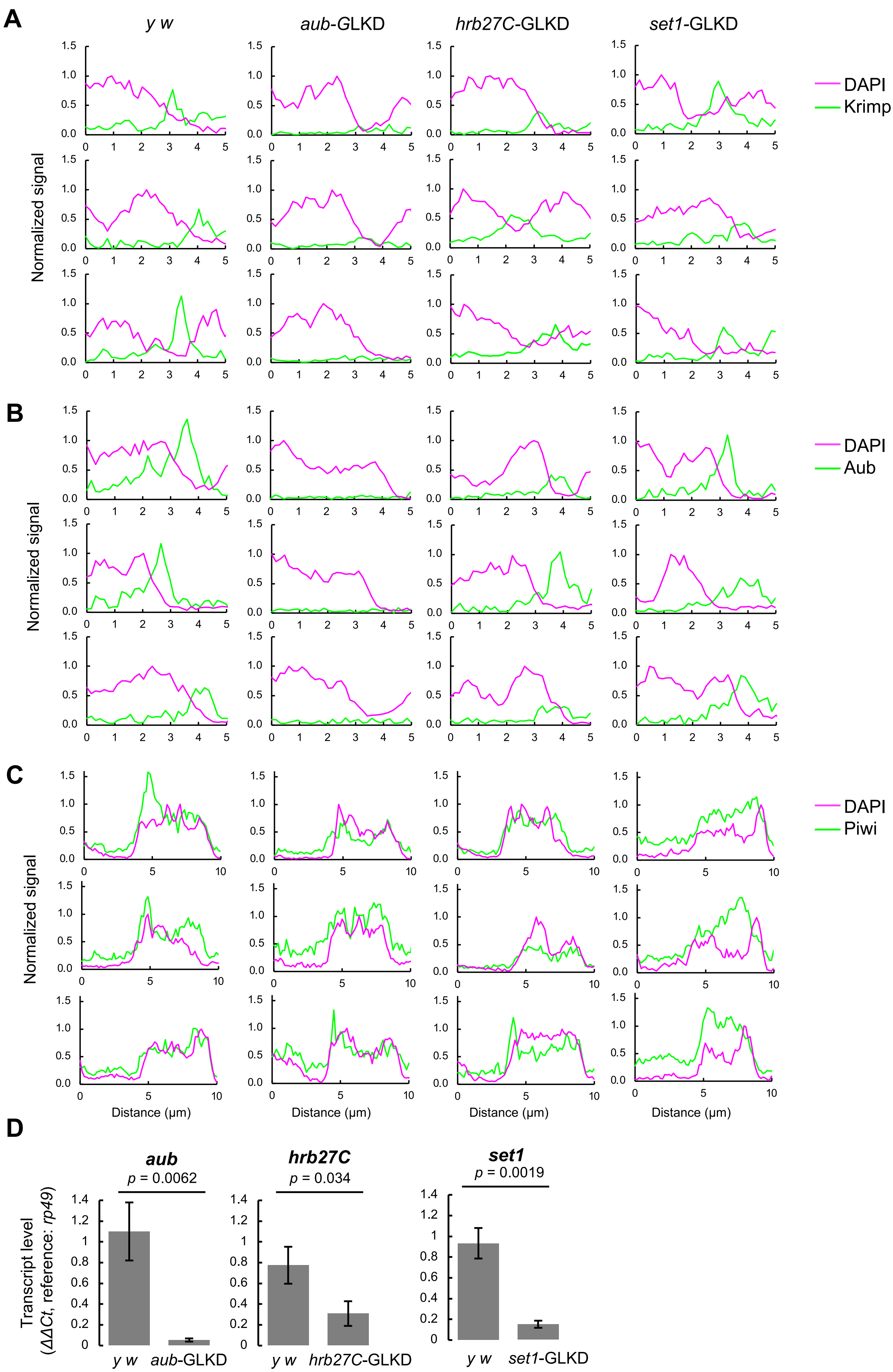
A
y w
aub-GLKD
hrb27C-GLKD
set1-GLKD
DAPI
Krimp
Normalized signal
B
DAPI
Aub
Normalized signal
C
DAPI
Piwi
Normalized signal
Distance (µm)
D
aub
p = 0.0062
hrb27C
p = 0.034
set1
p = 0.0019
Transcript level
(ΔΔCt, reference: rp49)
y w
aub-GLKD
hrb27C-GLKD
set1-GLKD

© The Author(s)

Figure EV3. Germline knockdown (GLKD) efficiency and the effect on protein subcellular localization.

(**A–C**) Plotting the fluorescence signals from egg chambers of the indicated genotypes, related to Fig. 3B. (**A**) Krimp (green) and DAPI (magenta), (**B**) Aub (green) and DAPI (magenta), (**C**) Piwi (green) and DAPI (magenta). (**D**) Transcript levels of *aub*, *hrb27C*, and *set1* in individual GLKD conditions (*ΔΔ*Ct method using *rp49* as reference). Error bars indicate standard deviation (SD) from biological replicates ($n = 3$). The center of error bars: average values. *P* values: unpaired Student's *t* test.

 © The Author(s)

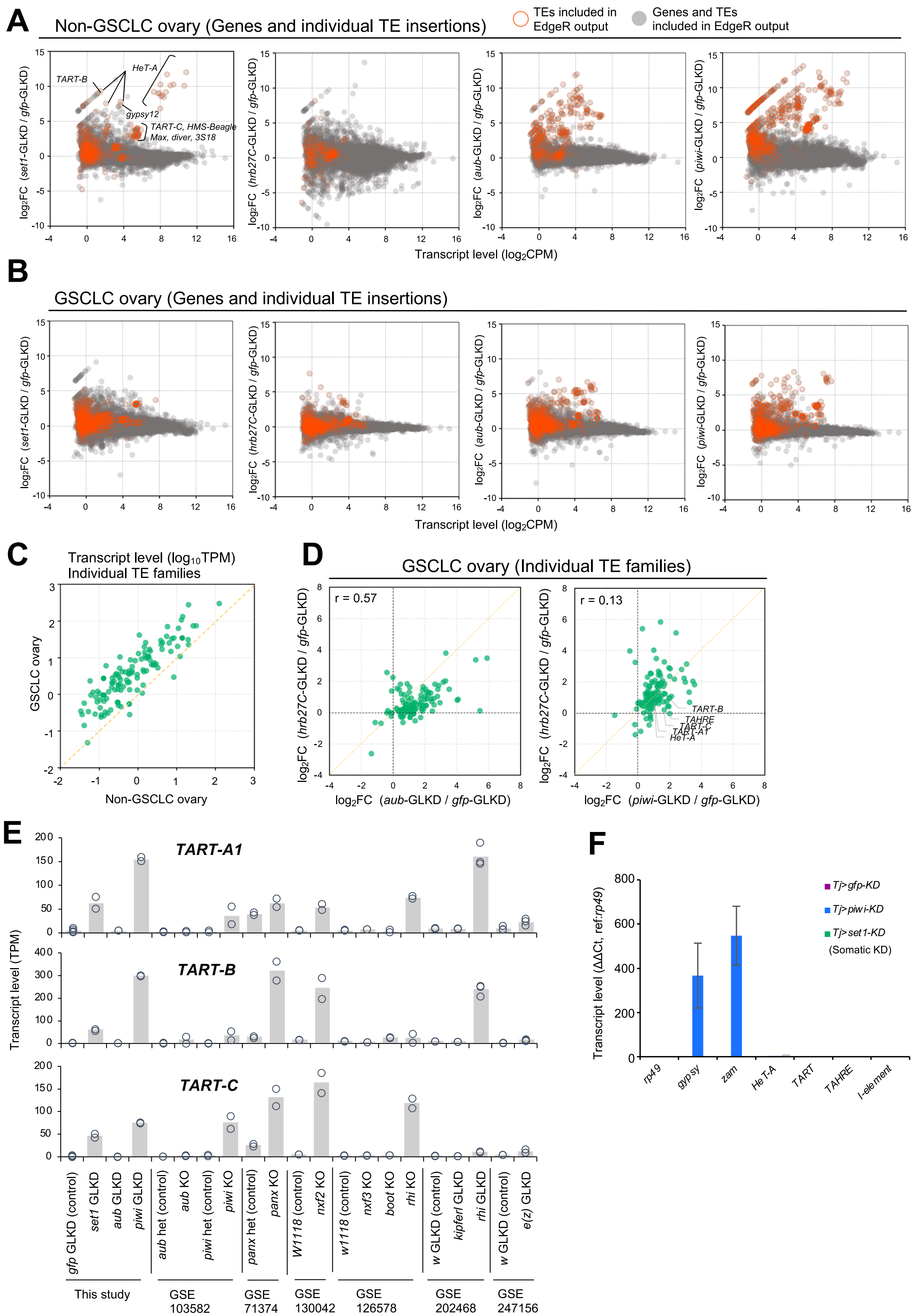
A
Non-GSCLC ovary (Genes and individual TE insertions)
TEs included in EdgeR output
Genes and TEs included in EdgeR output
TART-B
HeT-A
gypsy12
TART-C, HMS-Beagle
Max, diver, 3S18
log2FC (set1-GLKD / gfp-GLKD)
log2FC (hrb27C-GLKD / gfp-GLKD)
log2FC (aub-GLKD / gfp-GLKD)
log2FC (piwi-GLKD / gfp-GLKD)
Transcript level (log2CPM)
B
GSCLC ovary (Genes and individual TE insertions)
log2FC (set1-GLKD / gfp-GLKD)
log2FC (hrb27C-GLKD / gfp-GLKD)
log2FC (aub-GLKD / gfp-GLKD)
log2FC (piwi-GLKD / gfp-GLKD)
Transcript level (log2CPM)
C
Transcript level (log10TPM)
Individual TE families
GSCLC ovary
Non-GSCLC ovary
D
GSCLC ovary (Individual TE families)
r = 0.57
r = 0.13
log2FC (hrb27C-GLKD / gfp-GLKD)
log2FC (aub-GLKD / gfp-GLKD)
log2FC (piwi-GLKD / gfp-GLKD)
TART-B
TAHRE
TART-C
TART-A1
HeT-A
E
Transcript level (TPM)
TART-A1
TART-B
TART-C
gfp GLKD (control)
set1 GLKD
aub GLKD
piwi GLKD
aub het (control)
aub KO
piwi het (control)
piwi KO
panx het (control)
panx KO
W1118 (control)
nxf2 KO
w1118 (control)
nxf3 KO
boot KO
rhi KO
w GLKD (control)
kipferl GLKD
rhi GLKD
w GLKD (control)
e(z) GLKD
This study
GSE 103582
GSE 71374
GSE 130042
GSE 126578
GSE 202468
GSE 247156
F
Transcript level (ΔΔCt, ref.:rp49)
Tj>gfp-KD
Tj>piwi-KD
Tj>set1-KD
(Somatic KD)
rp49
gypsy
zam
HeT-A
TART
TAHRE
I-element

© The Author(s)

Figure EV4. GLKD effect on the ovary transcriptome, and somatic KD effect on TEs.

(**A**, **B**) MA plots comparing transcript levels (TPM) of genes and TE insertions in *set1*-, *hrb27C*-, *aub*-, or *piwi*-GLKD with those in control *gfp*-GLKD. Dots: genes (gray) and TE insertions (gray with red outline), included by EdgeR in differential expression analysis. Non-GSCLC (A) or GSCLC ovary conditions (**B**). (**C**) Scatter plot comparing transcript levels (TPM) of individual TE families (consensus sequences) in *gfp*-GLKD between non-GSCLC and GSCLC conditions. (**D**) Scatter plot comparing GLKD effect on transcript levels of individual TE families. Left: *hrb27C*-GLKD compared with *aub*-GLKD. Right: *hrb27C*-GLKD compared with *piwi*-GLKD. Fold changes (FC) from control *gfp*-GLKD are shown (TPM/TPM). r = Pearson correlation coefficient. (**E**) Transcript levels (TPM) for *TART-A1*, *TART-B*, and *TART-C*, in different transcriptome datasets. GSE103582 for *aub* and *piwi* knockout conditions. GSE71374 for *panx* knockout condition. Bars: mean values. Dots: individual values of biological replicates. (**F**) RT-qPCR measurement of TE transcript levels in ovaries upon somatic KD of *gfp*, *piwi*, or *set1* using *tj*-Gal4 driver. ΔΔCt method using *rp49* as reference. Error bars indicate ±SD from biological replicates ($n = 3$).

 © The Author(s)

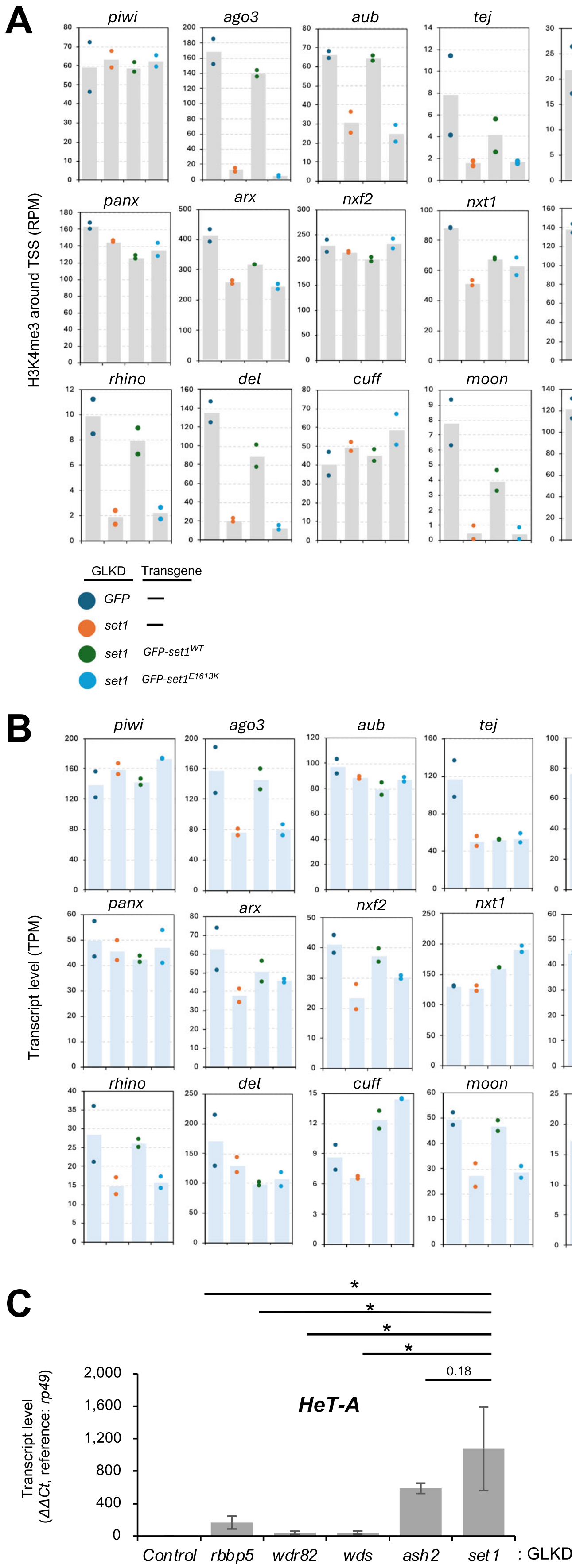
A
H3K4me3 around TSS (RPM)
piwi
ago3
aub
tej
krimp
spn-E
vas
shu
panx
arx
nxf2
nxt1
vret
wde
su(var)205
egg
rhino
del
cuff
moon
nxf3
boot
kipferl
zuc
GLKD
Transgene
GFP
set1
set1 GFP-set1WT
set1 GFP-set1E1613K
B
Transcript level (TPM)
C
HeT-A
Transcript level (ΔΔCt, reference: rp49)
2,000
1,600
1,200
800
400
0
0.18
Control
rbbp5
wdr82
wds
ash2
set1
: GLKD

Figure EV5. Effect of *set1*-GLKD on the expression of piRNA pathway factors, and effect of COMPASS subunit GLKD on *HeT-A* expression.

(**A**) Bar graph showing mean H3K4me3 signal (RPM) around TSS (± 500 bp) of piRNA pathway genes. Dots: individual RPM from biological replicates ($n = 2$). (**B**) Bar graph showing mean transcript levels (TPM). Dots: individual TPM from biological replicates ($n = 2$). (**C**) RT-qPCR measurement of *HeT-A* transcript levels in ovaries depleting individual COMPASS subunits from germline cells. ΔΔ*Ct* method using *rp49* as a reference. Error bars: ±SD from biological replicates ($n = 3$). Asterisk: $P < 0.05$ (unpaired Student's *t* test).

 © The Author(s)

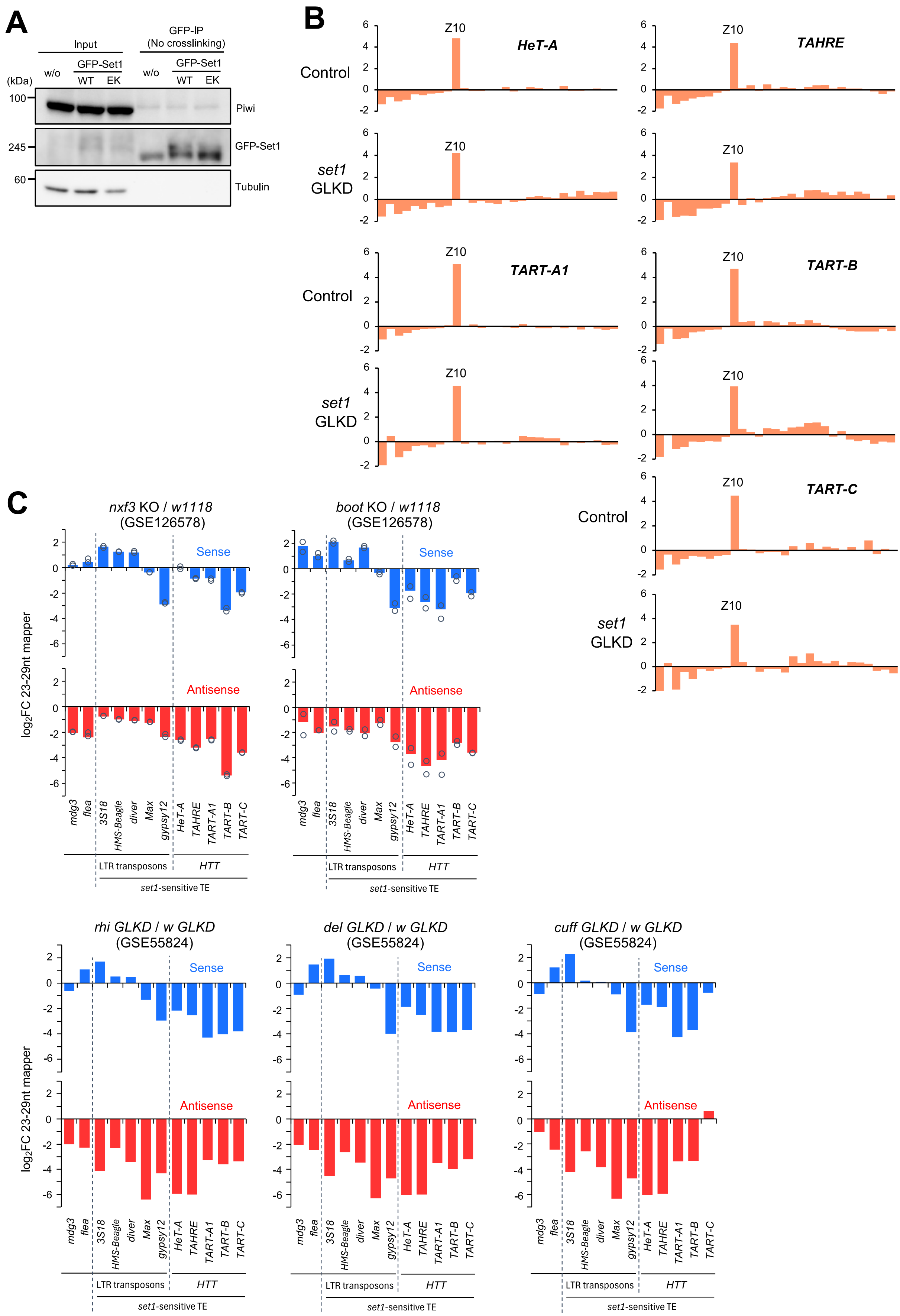
A
Input
GFP-IP
(No crosslinking)
w/o
GFP-Set1
WT EK
(kDa)
100
245
60
Piwi
GFP-Set1
Tubulin
B
Control
set1
GLKD
HeT-A
TAHRE
TART-A1
TART-B
TART-C
Z10
C
nxf3 KO / w1118
(GSE126578)
boot KO / w1118
(GSE126578)
rhi GLKD / w GLKD
(GSE55824)
del GLKD / w GLKD
(GSE55824)
cuff GLKD / w GLKD
(GSE55824)
Sense
Antisense
log2FC 23-29nt mapper
mdg3
flea
3S18
HMS-Beagle
diver
Max
gypsy12
HeT-A
TAHRE
TART-A1
TART-B
TART-C
LTR transposons
HTT
set1-sensitive TE

◀ **Figure EV6. Immunoprecipitation of GFP-Set1 without crosslinking, effect of *set1*-GLKD on piRNA ping-pong signature, TE-mapping piRNA levels in ovaries lacking nxf3, boot, rhi, del, or cuff.**

(**A**) Immunoprecipitation of GFP-Set1 proteins from ovaries using anti-GFP antibody (GFP-IP) without crosslinking treatment. W/o: sibling control without GFP-Set1 expression. Tubulin serves as a loading control. (**B**) Ping-pong signature analysis on piRNAs mapping to telomeric TE families. Z10 scores (reflecting 10-nt overlap frequency within piRNA groups) were highlighted. Values are means of biological replicates ($n = 2$). (**C**) Bar graph showing mean fold change (FC) of piRNA abundance in mutants relative to their respective controls. Raw data from GSE126578 and GSE55824 were re-analyzed using the same pipeline. GSE126578 includes biological replicates ($n = 2$), and individual values are shown as dots.

 © The Author(s)

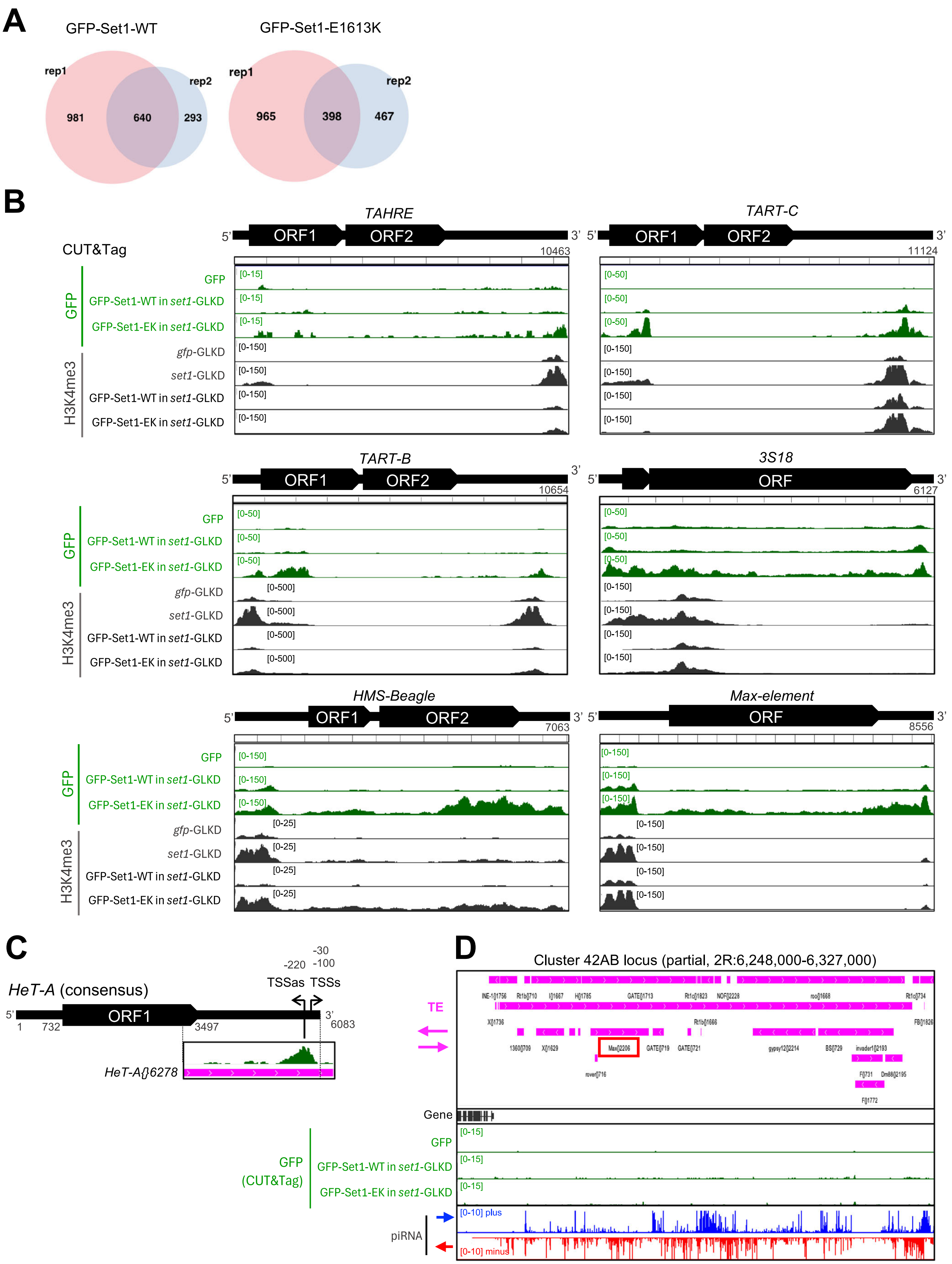
A
GFP-Set1-WT
rep1
rep2
981
640
293
GFP-Set1-E1613K
rep1
rep2
965
398
467
B
CUT&Tag
TAHRE
ORF1
ORF2
10463
TART-C
11124
TART-B
10654
3S18
ORF
6127
HMS-Beagle
7063
Max-element
8556
GFP
GFP-Set1-WT in set1-GLKD
GFP-Set1-EK in set1-GLKD
H3K4me3
gfp-GLKD
set1-GLKD
C
HeT-A (consensus)
ORF1
TSSas TSSs
-220 -100
-30
1 732 3497 6083
HeT-A{}6278
D
Cluster 42AB locus (partial, 2R:6,248,000-6,327,000)
TE
Gene
GFP (CUT&Tag)
piRNA
[0-10] plus
[0-10] minus

Figure EV7. Genome-wide analysis of Set1 binding by CUT&Tag.

(**A**) Venn diagram showing overlap of Set1-binding peak-associated genes and TEs between biological replicates ($n = 2$). Peak called by MACS2 comparing GFP-Set1^{WT} and GFP, or GFP-Set1^{E1613K} and GFP ($p < 0.01$, Poisson test). (**B**) Track view for CUT&Tag signals of GFP proteins (green) and H3K4me3 (black) on TE consensus sequences. ORFs were predicted by ORF Finder. (**C**) Alignment of *HeT-A{}6278* with GFP-Set1^{E1613K} binding signals to *HeT-A* consensus sequence. (**D**) Track view for anti-GFP CUT&Tag signals at *42AB* locus (partial). Blue and red: piRNAs mapped to the plus and minus strands, respectively.

 © The Author(s)

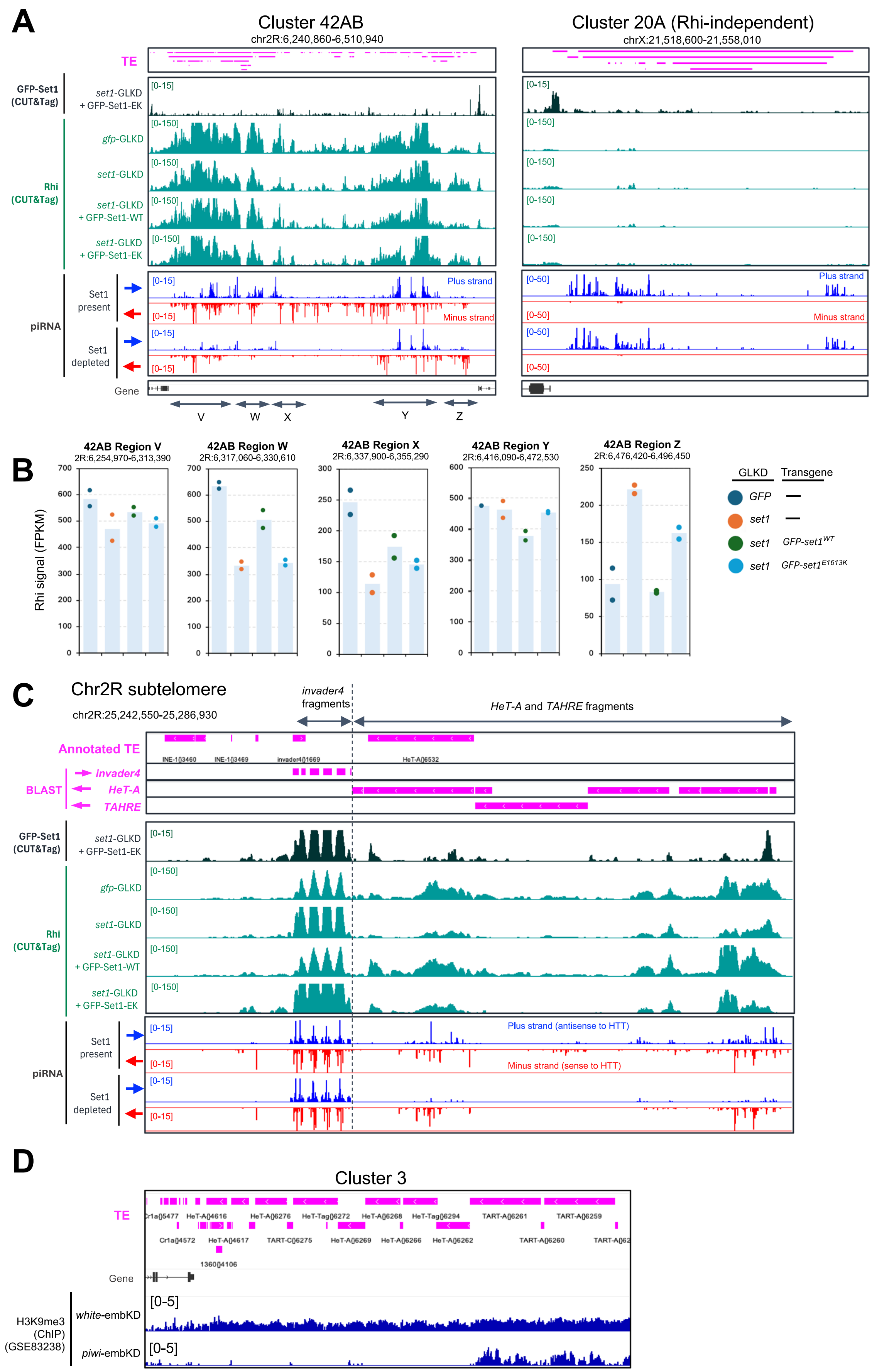
A
Cluster 42AB
chr2R:6,240,860-6,510,940
Cluster 20A (Rhi-independent)
chrX:21,518,600-21,558,010
TE
GFP-Set1 (CUT&Tag)
set1-GLKD + GFP-Set1-EK
Rhi (CUT&Tag)
gfp-GLKD
set1-GLKD
set1-GLKD + GFP-Set1-WT
set1-GLKD + GFP-Set1-EK
piRNA
Set1 present
Set1 depleted
Plus strand
Minus strand
Gene
V W X Y Z
B
42AB Region V
2R:6,254,970-6,313,390
42AB Region W
2R:6,317,060-6,330,610
42AB Region X
2R:6,337,900-6,355,290
42AB Region Y
2R:6,416,090-6,472,530
42AB Region Z
2R:6,476,420-6,496,450
Rhi signal (FPKM)
GLKD Transgene
GFP —
set1 —
set1 GFP-set1WT
set1 GFP-set1E1613K
C
Chr2R subtelomere
chr2R:25,242,550-25,286,930
invader4 fragments
HeT-A and TAHRE fragments
Annotated TE
BLAST
invader4
HeT-A
TAHRE
GFP-Set1 (CUT&Tag)
Rhi (CUT&Tag)
piRNA
Plus strand (antisense to HTT)
Minus strand (sense to HTT)
D
Cluster 3
TE
Gene
H3K9me3 (ChIP) (GSE83238)
white-embKD
piwi-embKD

Figure EV8. Effect of Set1 depletion on Rhi localization at piRNA cluster loci, and effect of Piwi depletion on H3K9me3 deposition in subtelomeric cluster 3.

(**A**) Track view at 42AB or 20A locus. Light green: signals from anti-Rhi CUT&Tag. Dark green: GFP-Set1-EK signals (anti-GFP CUT&Tag). Blue and red: piRNAs mapped to genomic plus and minus strands, respectively. (**B**) Quantification of anti-Rhi CUT&Tag signals. Bars: mean FPKM, dots: individual FPKM, from biological replicates ($n = 2$). Analyzed regions within 42AB are indicated in (**A**). (**C**) Track view in the subtelomeric region on chromosome 2R. (**D**) Track view at subtelomeric cluster 3 showing H3K9me3 signal (mean CPM from biological replicates ($n = 2$)). embKD: knockdown at embryonic stages. Raw data: GSE83238.

 © The Author(s)
