# Supplementary material for: PIWI proximity proteome reveals Set1-mediated piRNA biogenesis for transposon silencing at telomeres
Source: EMBO Rep. 2026 Aug 26;27(19):5490–529. doi: 10.1038/s44319-026-00901-6 (PMC13650161; doi:10.1038/s44319-026-00901-6)
Supplement: Supplementary file 2 [file 44319_2026_901_MOESM5_ESM.pdf]

PIWI proximity proteome reveals Set1-mediated piRNA biogenesis for transposon silencing at telomeres

Wakana Isshiki, Hiroko Kozuka-Hata, Masaaki Oyama, Toshie Kai, and Taichiro Iki

Corresponding author(s): Taichiro Iki (iki.taichiro.8d@kyoto-u.ac.jp) , Toshie Kai (kai.toshie.fbs@osaka-u.ac.jp)

Review Timeline:

Submission Date:	31st Mar 26
Editorial Decision:	7th May 26
Revision Received:	29th Jun 26
Editorial Decision:	20th Jul 26
Revision Received:	22nd Jul 26
Accepted:	4th Aug 26

Editor: Esther Schnapp

Transaction Report:

(Note: With the exception of the correction of typographical or spelling errors that could be a source of ambiguity, letters and reports are not edited. Depending on transfer agreements, referee reports obtained elsewhere may or may not be included in this compilation. Referee reports are anonymous unless the Referee chooses to sign their reports.)

Dear Dr. Iki,

Thank you for the submission of your manuscript to EMBO reports. We have now received the full set of referee reports that is pasted below.

As you will see, all referees acknowledge that the findings are potentially interesting and novel. However, both referees 1 and 2 also point out that some more insight is required before the study can be considered for publication here. Referee 2 provides constructive suggestions and I think all of these should be addressed. Please let me know if you have any questions or comments regarding the revisions and we can discuss them further, also in a video chat, if you like.

I would thus like to invite you to revise your manuscript with the understanding that the referee concerns must be fully addressed and their suggestions taken on board. Please address all referee concerns in a complete point-by-point response.

Acceptance of the manuscript will depend on a positive outcome of a second round of review. It is EMBO reports policy to allow a single round of major revision only and acceptance or rejection of the manuscript will therefore depend on the completeness of your responses included in the next, final version of the manuscript.

We realize that it is difficult to revise to a specific deadline. In the interest of protecting the conceptual advance provided by the work, we recommend a revision within 3 months (7th Aug 2026). Please discuss the revision progress ahead of this time with the editor if you require more time to complete the revisions.

You can either publish the study as a short report or as a full article. For short reports, the revised manuscript should not exceed 27,000 characters (including spaces but excluding materials & methods and references) and 5 main plus 5 expanded view figures. The results and discussion sections must further be combined, which will help to shorten the manuscript text by eliminating some redundancy that is inevitable when discussing the same experiments twice. For a normal article there are no length limitations, but it should have more than 5 main figures and the results and discussion sections must be separate. In both cases, the entire materials and methods must be included in the main manuscript file.

IMPORTANT NOTE: we perform an initial quality control of all revised manuscripts before re-review. Your manuscript will FAIL this control and the handling will be DELAYED if the following APPLIES:
1) A data availability section providing access to data deposited in public databases is missing. If you have not deposited any data, please add a sentence to the data availability section that explains that.
2) Your manuscript contains statistics and error bars based on n=2. Please use scatter blots in these cases. No statistics should be calculated if n=2.

When submitting your revised manuscript, please review the submission guidelines (https://link.springer.com/journal/44319/submission-guidelines#cms-Revised-submissions) and carefully review the instructions that follow below. Failure to include requested items will delay the evaluation of your revision.

1) a .docx formatted version of the manuscript text (including legends for main figures, EV figures and tables). Please make sure that the changes are highlighted to be clearly visible.

2) individual production quality figure files as .eps, .tif, .jpg (one file per figure). See hhttps://media.springernature.com/original/springer-cms/rest/v1/content/27825798/data/v1 for more info on how to prepare your figures.

3) We replaced Supplementary Information with Expanded View (EV) Figures and Tables that are collapsible/expandable online. A maximum of 5 EV Figures can be typeset. EV Figures should be cited as 'Figure EV1, Figure EV2" etc... in the text and their respective legends should be included in the main text after the legends of regular figures.

- For the figures that you do NOT wish to display as Expanded View figures, they should be bundled together with their legends in a single PDF file called *Appendix*, which should start with a short Table of Content. Appendix figures should be referred to in the main text as: "Appendix Figure S1, Appendix Figure S2" etc. See 'Expanded View' section of the submission guidelines.

- Additional Tables/Datasets should be labeled and referred to as Table EV1, Dataset EV1, etc. Legends have to be provided in a separate tab in case of .xls files. Alternatively, the legend can be supplied as a separate text file (README) and zipped together with the Table/Dataset file.

4) a .docx formatted letter INCLUDING the reviewers' reports and your detailed point-by-point responses to their comments. As part of the EMBO Press transparent editorial process, the point-by-point response is part of the Review Process File (RPF), which will be published alongside your paper.

5) a complete author checklist, which you can download from our author guidelines. Please insert information in the checklist that is also reflected in the manuscript. The completed author checklist will also be part of the RPF.

6) Please note that all corresponding authors are required to supply an ORCID ID for their name upon submission of a revised manuscript (<https://orcid.org/>), which can be linked to your profile in the manuscript submission system.

7) Before submitting your revision, primary datasets produced in this study need to be deposited in an appropriate public database. Please remember to provide a reviewer password if the datasets are not yet public. The accession numbers and database should be listed in a formal "Data Availability" section placed after Methods. Please note that the Data Availability Section is restricted to new primary data that are part of this study. * Note - All links should resolve to a page where the data can be accessed. *

If your study has not produced novel datasets, please mention this fact in the Data Availability Section.

8) At EMBO Press we ask authors to provide source data for the main manuscript figures. You will receive a separate email with instructions for providing source data with your revised manuscript, including how to upload and organize the files.

9) Our journal also encourages inclusion of *data citations in the reference list* to directly cite datasets that were re-used and obtained from public databases. Data citations in the article text are distinct from normal bibliographical citations and should directly link to the database records from which the data can be accessed. In the main text, data citations are formatted as follows: "Data ref: Smith et al, 2001" or "Data ref: NCBI Sequence Read Archive PRJNA342805, 2017". In the Reference list, data citations must be labeled with "[DATASET]". A data reference must provide the database name, accession number/identifiers and a resolvable link to the landing page from which the data can be accessed at the end of the reference.

10) Regarding data quantification, the following points must be specified in each figure legend:

- the name of the statistical test used to generate error bars and P values,

- the number (n) of independent experiments (please specify technical or biological replicates) underlying each data point,

- the nature of the bars and error bars (s.d., s.e.m.),

- If the data are obtained from n Program fragment delivered error ``Can't locate object method "less" via package "than" (perhaps you forgot to load "than"?) at //ejpvfs23/sites23b/embor_www/letters/embor_decision_revise_and_review.txt line 54.' 2, use scatter blots showing the individual data points.

Discussion of statistical methodology can be reported in the materials and methods section, but figure legends should contain a basic description of n, P and the test applied.

- Please also include scale bars in all microscopy images.

11) The journal requires a statement specifying whether or not authors have competing interests (defined as all potential or actual interests that could be perceived to influence the presentation or interpretation of an article). In case of competing interests, this must be specified in your disclosure statement.

12) All Materials and Methods need to be described in the main text using our 'Structured Methods' format, which is required for all research articles. According to this format, the Methods section includes a separate Reagents and Tools Table file (listing key reagents, experimental models, software and relevant equipment and including their sources and relevant identifiers) and a Methods and Protocols section describing the methods using a step-by-step protocol format. The aim is to facilitate adoption of the methodologies across labs. More information on how to adhere to this format as well as a downloadable template (.docx) for the Reagents and Tools Table can be found in our author guidelines.

We would also welcome the submission of cover suggestions, or motifs to be used by our Graphics Illustrator in designing a cover.

As part of the EMBO publication's Transparent Editorial Process, EMBO reports publishes online a Review Process File (RPF) to accompany accepted manuscripts. This File will be published in conjunction with your paper and will include the referee reports, your point-by-point response and all pertinent correspondence relating to the manuscript.

You are able to opt out of this by letting the editorial office know (emboreports@embo.org). If you do opt out, the Review Process File link will point to the following statement: "No Review Process File is available with this article, as the authors have chosen not to make the review process public in this case."

I look forward to seeing a revised form of your manuscript when it is ready. Please use this link to submit your revision:

https://embor.msubmit.net/cgi-bin/main.plex

Best regards,
Esther

Esther Schnapp, PhD
Senior Editor
EMBO reports

Referee #1:

Isshiki and colleagues present a comprehensive analysis of PIWI proximity proteomes and identify Set1 as a factor required for the silencing of HTT transposons and for antisense piRNA production in the Drosophila germline. The study combines proteomics, genetics, transcriptomics, and chromatin profiling, and the dataset is generally of high quality. In particular, the observation that catalytically inactive Set1 plays a role in the regulation of telomeric transposable elements and in antisense piRNA production is intriguing and potentially important.
However, despite the richness of the data, the underlying mechanism remains unclear. The current model appears to conflate several possible roles of Set1, making it difficult to distinguish whether the observed phenotypes reflect a direct function in the Piwi-piRNA pathway or a broader role of Set1 in chromatin regulation and transcription. As a result, the study feels descriptive and somewhat inconclusive in its present form. Given that the findings remain largely superficial, it is difficult to judge whether the work, in its current state, is suitable for publication in this journal. This is particularly unfortunate because the dataset clearly has the potential to develop into a much stronger study with additional mechanistic data.
Major concerns
1.: A central issue is the absence of a clear mechanistic framework explaining how non-catalytic Set1 specifically contributes to antisense piRNA abundance of telomeric HeT-A.
2: It remains unclear whether the Piwi-Set1 interaction is required for Set1-mediated telomere regulation.
Minor concerns
Line 230: "immunotaining" should be corrected to "immunostaining."

Referee #2:

Summary
To better understand how transposon silencing by the piRNA pathway is established, Isshiki and colleagues set out to characterize the proximal interactome of the PIWI-clade Argonaute proteins Piwi, Aubergine (Aub), and Ago3 in the Drosophila female germline. The authors employed nanos-driven TurboID fusion constructs of the respective PIWI proteins to biotinylate proximal factors. Due to technical challenges, they enriched for germline stem cell-like cells (GSCLCs) to achieve a robust pulldown of biotinylated targets. They then investigated the functional role of uncharacterized piRNA pathway candidates by testing the effect of their knockdown on Krimper localization as a proxy for nuage integrity and on HeT-A Gag protein accumulation as a readout of transposon derepression. Results of this validation screen prompted the authors to follow up on Hrb27C and Set1 as putative effectors of Aub- and Piwi-mediated silencing, respectively. Further experiments revealed that Hrb27C exerts TE repression in GSCLCs only upon bam depletion, with no detectable effect under standard knockdown conditions. The remainder of the manuscript focuses on Set1, an H3K4 histone methyltransferase required for TE silencing in germline cells. Rescue experiments with RNAi-resistant constructs demonstrate that a catalytic mutant of Set1 only partially rescues the silencing phenotype, indicating an H3K4 methylation-independent contribution of Set1. Profiling of piRNAs from Set1-depleted ovaries reveals a selective reduction in piRNAs mapping to telomeric transposable elements. Finally, CUT&TAG profiling of H3K4me3 and Set1 occupancy shows that wild-type Set1 binds most strongly at gene loci, whereas the catalytic-dead version displays a higher proportion of peaks overlapping with TE loci. Finally the authors show binding of Set1 to subtelomeric piRNA cluster 3, which contains truncated HeT-A 3' UTR insertions and suggest that Set1 promotes its piRNA precursor transcription.

Summary assessment
The manuscript reports an interesting observation linking Set1 to TE silencing and piRNA biology. However, some points remain incomplete (see below). The central concern is that the manuscript does not adequately address the potential effect of Set1 loss on Rhino, the well-established master regulator of piRNA cluster transcription in Drosophila ovaries. Even a partial reduction in Rhino activity, which might not lead to detectable nuage disassembly phenotype, could account for the observed silencing defects. While the manuscript provides an interesting observation of Set1's role in transposon silencing the points should be addressed before publication.

Major points
1. Relationship between Set1 and Rhino-dependent piRNA cluster regulation
The potential impact of Set1 on Rhino biology is insufficiently adressed in the manuscript. Given that Rhino is a well established

master regulator of dual-strand piRNA clusters, it is critical to assess whether Set1 depletion affects Rhino localization or occupancy at piRNA clusters. The authors should include ChIP-seq profiling of Rhino in Set1-depleted ovaries. In addition, comparison of the observed piRNA and TE derepression phenotypes with published datasets from mutants of Rhino-associated factors (Rhino, Deadlock, Cutoff, Moonshiner, Nxf3, Kipferl, and others) would substantially strengthen the interpretation and help to place a Set1-specific effect from a general Rhino pathway perturbation.
2. Transcriptomic characterization of GFP-Set1WT versus GFP-Set1E1613K
An RNA-seq comparison between GFP-Set1WT and GFP-Set1E1613K expressing cells is currently lacking. MA plots or volcano plots distinguishing effects on protein-coding genes from effects on TEs would allow a more rigorous assessment of the catalytic versus non-catalytic contributions of Set1 to the observed phenotypes and would directly support the rescue experiment interpretation. The comparison to a full depletion of Set1 and the different rescue constructs might help the interpretation of the catalytic mutant phenotype.
3. Incompleteness of the piRNA pathway factor list in Figure S5A
The piRNA pathway factor list used for the validation screen (Figure S5A) is incomplete. Notably, multiple Rhino-associated factors are absent. This is a significant gap given the central relevance of Rhino biology in piRNA cluster transcription and therefore to the manuscript's conclusions.
4. Hrb27C data are too preliminary to be conclusive
The Hrb27C data currently show TE derepression only in the context of bam depletion in GSCLCs, with no detectable effect under standard knockdown conditions. Without demonstrating that TEs are affected in bona fide GSCs under non-bam-depletion conditions, for example by RNA FISH, the significance of this finding is unclear. The authors should either provide this additional evidence or consider removing the Hrb27C section, as it does not substantially advance the manuscript's main conclusions in its current form.

Figures and presentation:
1. Immunofluorescence image presentation:
All IF images should include single-channel views alongside the merged panels to allow proper assessment of signal specificity and colocalization (affected figures: Fig. 1B, Fig. 5B).
2. Mass spectrometry data (Figure 2A):
The presentation of the mass spectrometry data should be expanded by having volcano plots of the combined replicates. This will allow the readers to judge the statistical power and log2FC of the individual hits.
3. Comparison of GLKD screen results to published germline TE regulation data: The results of the germline knockdown (GLKD) screen should be compared to published genome-wide RNAi screen data for germline TE regulation, which would provide an orthogonal functional readout complementary to the IF-based scoring used here (Czech et al., 2013, Mol Cell 50:749-61; PMID: 23665227).
4. Figure S3A
The quantification of the localization phenotypes is hard to distinguish as all measured proteins have the same color (green). It would help the readers to associated them if different colors are used.
5. Figure 1 & 2
These figures could be combined as the data presented are strongly linked to provide a coherent and accessible flow.
6. Figure 7E
Genome browser screenshots for piRNA levels still show scale factors in the background (black letters) this should be removed to avoid confusion

Referee #3:

I loved the paper. Through careful and comprehensive experiments, they have revealed a hitherto unknown function of SET1, which is a big advance.

The technical details are impeccable, but I feel the title undersells the paper, an alternative title such as "Set1-Mediated Antisense Transcription: A Methyltransferase-Independent Guard of the Drosophila Telomere" or ""Set1 Acts as a Catalytic-Independent Scaffold to Recruit the Piwi Pathway for Telomere Protection"" would help readers discern what is special in the paper and help drive more people to read it.

I believe any editing should be in the abstract and title to make it more impactful and help "sell" the paper to the audience. The way the paper is written is a little underwhelming. So I would shorten the paper a little bit and highlight the surprising parts, and let the piwi-proximity etc. (which led to SET1) be supporting actors. As you can see, I don't want to quibble about minor things in this paper, but want it to be more bold with its message.

Through painstaking experiments silencing 54 potential targets, they identified Set1 and Hrb27C as the most promising candidates for further study. While Hrb27C was linked to Aubergine, Set1 stood out because its depletion caused a significant accumulation of the HeT-A Gag protein, directly linking it to the silencing of telomeric transposons .

The paper is packed with several technical achievements too, such as the methodological optimization of mTurbo for the female

germline. They identified that standard purification was inefficient in differentiating ovaries and overcame this by using GSCLCs (Germline Stem Cell-Like Cells) via bam knockdown to dramatically improve pulldown efficiency.
The use of the RNAi-resistant Set1 E1613K mutant and normal Set1 to demonstrate the catalytic-activity-independent function of Set1 is novel.

Great job, and a very convincing and thorough work, with all the lines of evidence (proteomic, genetic, transcriptomic, small RNA etc.) that has been amassed. I don't want to mention anything negative for the sake of it. I genuinely did not see anything amiss, but maybe some of the material could be moved to supplementary, for readers who might need the gory details. My advice could be counter to the journal policy, which I am not familiar with.

We appreciate all the reviewers for their constructive and encouraging comments. Here, we prepared a revised manuscript containing changes following their suggestions. The revised manuscript has structural changes. We added Figure 8 containing new results with an updated model. Since EMBO Reports allow maximum 5 EV figures (collapsible/expandable online), we renamed Figure S4-S7 as Figure EV1-4. Figure EV5 was newly generated as the supplement for Figure 8. Figure S1-S3 were renamed as Appendix Figure S1-S3 following the journal rule.

Referee #1:

Isshiki and colleagues present a comprehensive analysis of PIWI proximity proteomes and identify Set1 as a factor required for the silencing of HTT transposons and for antisense piRNA production in the Drosophila germline. The study combines proteomics, genetics, transcriptomics, and chromatin profiling, and the dataset is generally of high quality. In particular, the observation that catalytically inactive Set1 plays a role in the regulation of telomeric transposable elements and in antisense piRNA production is intriguing and potentially important.
However, despite the richness of the data, the underlying mechanism remains unclear. The current model appears to **conflate** several possible roles of Set1, making it difficult to distinguish whether the observed phenotypes reflect a direct function in the Piwi-piRNA pathway or a broader role of Set1 in chromatin regulation and transcription. As a result, the study feels descriptive and somewhat **inconclusive** in its present form. Given that the findings remain largely **superficial**, it is difficult to judge whether the work, in its current state, is suitable for publication in this journal. This is particularly unfortunate because the dataset clearly has the potential to develop into a much stronger study with **additional mechanistic data**.
Thank you very much for your constructive comments. In the revised manuscript, we demonstrate that Set1 functions as a key player recruiting Rhi to subtelomeric *HTT* repeats. The results illustrating why Set1 is important in piRNA production and telomere TE silencing were included as new Figure 8 and Figure EV5.

Major concerns
1.: A central issue is the absence of a clear **mechanistic framework** explaining how non-catalytic Set1 specifically contributes to antisense piRNA abundance of telomeric HeT-A.
Thank you for the critical comment. To provide the mechanistic framework, we followed the advise from reviewer#2, and re-analyzed public piRNA and mRNA data for mutants of piRNA pathway components. This revealed the importance of Rhino (Rhi) in producing *HTT*-targeting piRNAs (new Figure 6E, attached below, top) and silencing *HTT* (new Figure 4D, attached below, bottom).

E

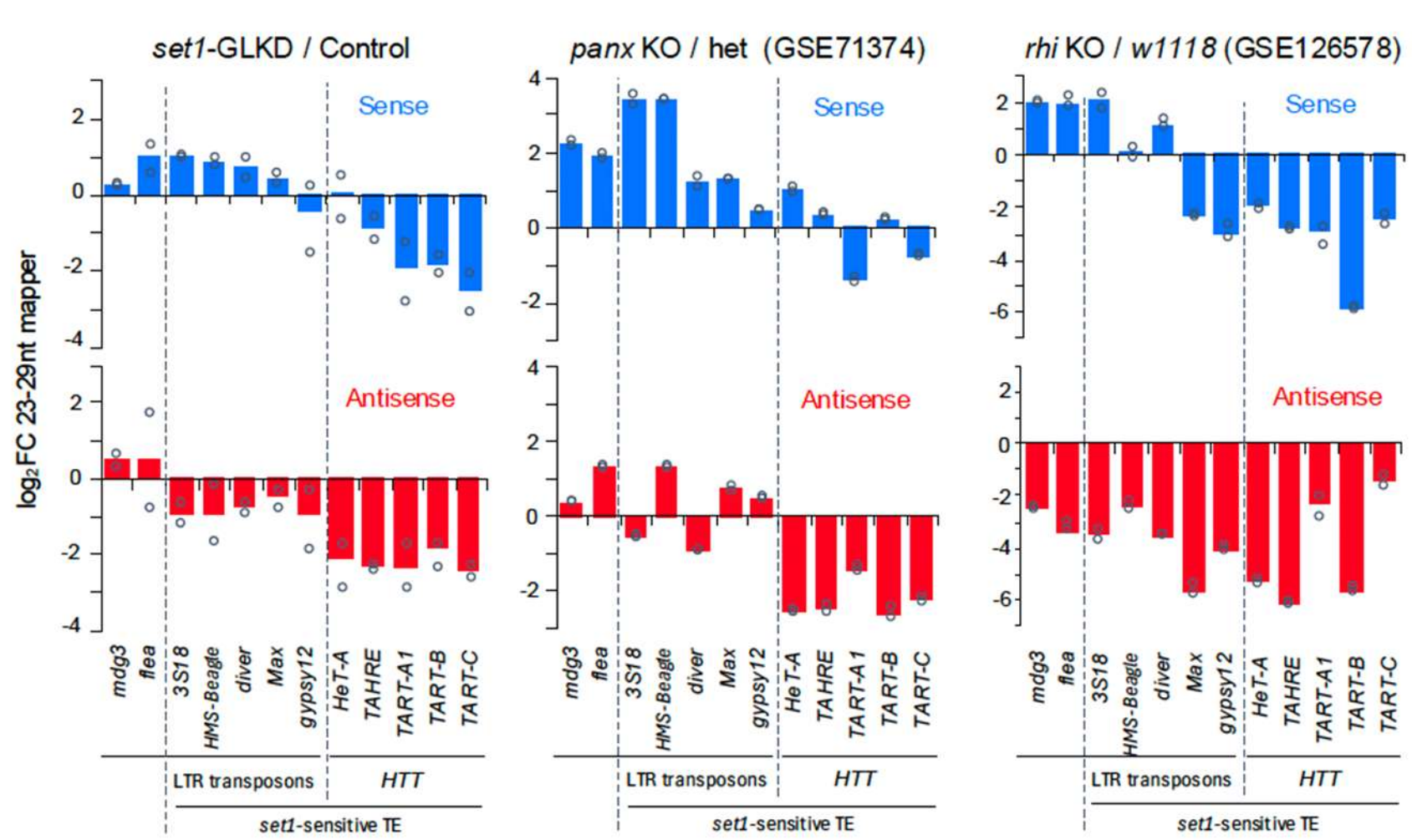

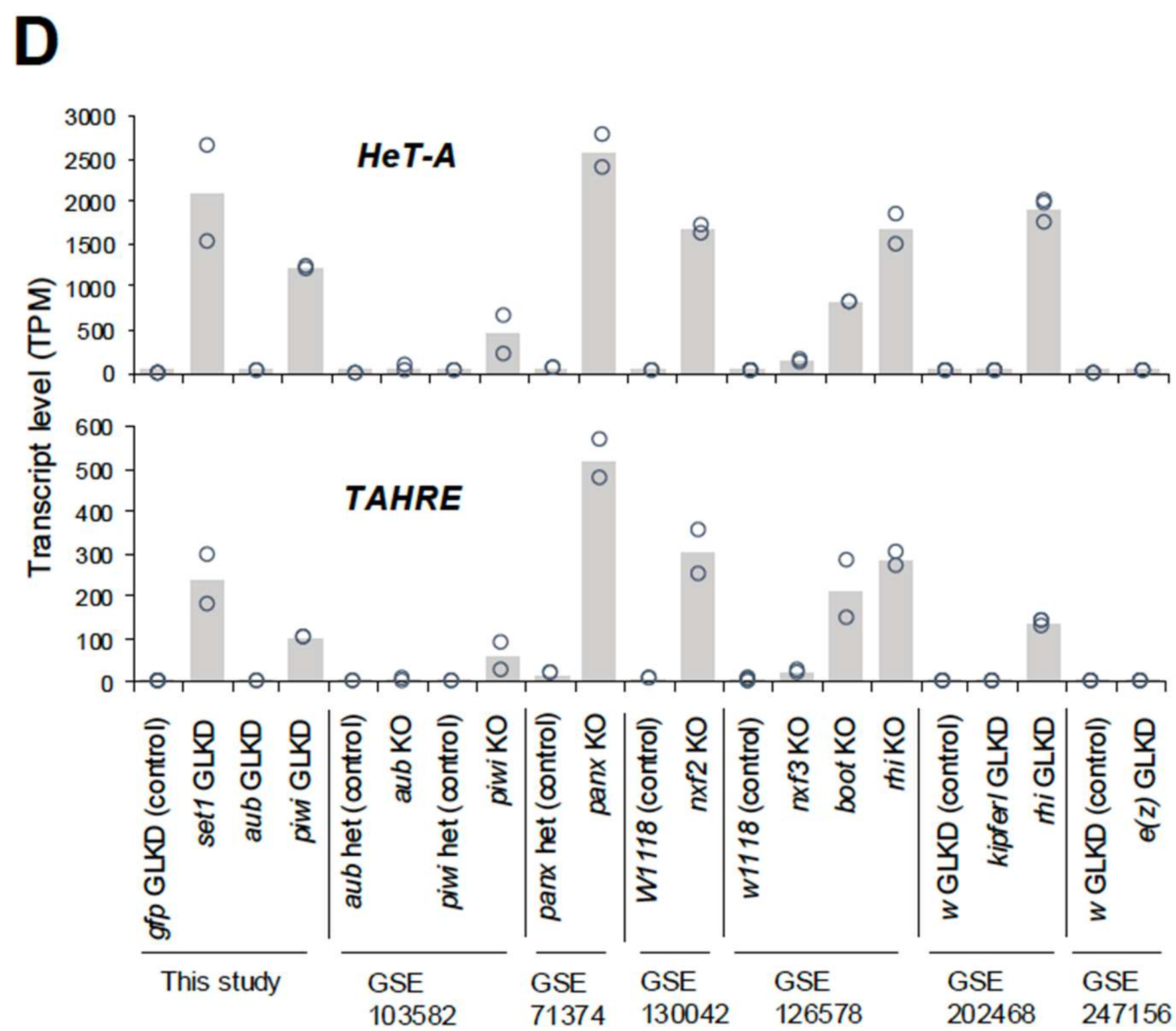

Rhi is an H3K9me3 reader and a key activator of piRNA precursor transcription from cluster loci in germline cells (Mohn et al., Cell, 2014). We examined if Set1 is required for Rhi activities.

First, we conducted anti-Rhi CUT&Tag and observed Rhi localization in major piRNA clusters (42AB, 38C, known to accumulate Rhi) as well as in subtelomeric cluster 3. Rhi was absent in 20A locus whose piRNA production is Rhi-independent. Next, Rhi signals were compared between the four conditions; (1) control *gfp*-GLKD (germline knockdown), (2) *set1*-GLKD, (3) *set1*-GLKD expressing GFP-Set1-WT, and (4) *set1*-GLKD expressing GFP-Set1-EK (catalytic mutant).

We found striking reduction in Rhi signals upon *set1*-GLKD in subtelomeric cluster 3 (see attachment below, new Figure 8AB). Rhi level was fully restored with GFP-Set1-WT, and less efficiently with GFP-Set1-EK. The pattern is similar to what we observed in TE rerepression by the transgene products (Figure 5C). Hence, Set1 recruits Rhi to subtelomeric cluster loci for piRNA production, which is supported by the catalytic activity-independent mechanism.

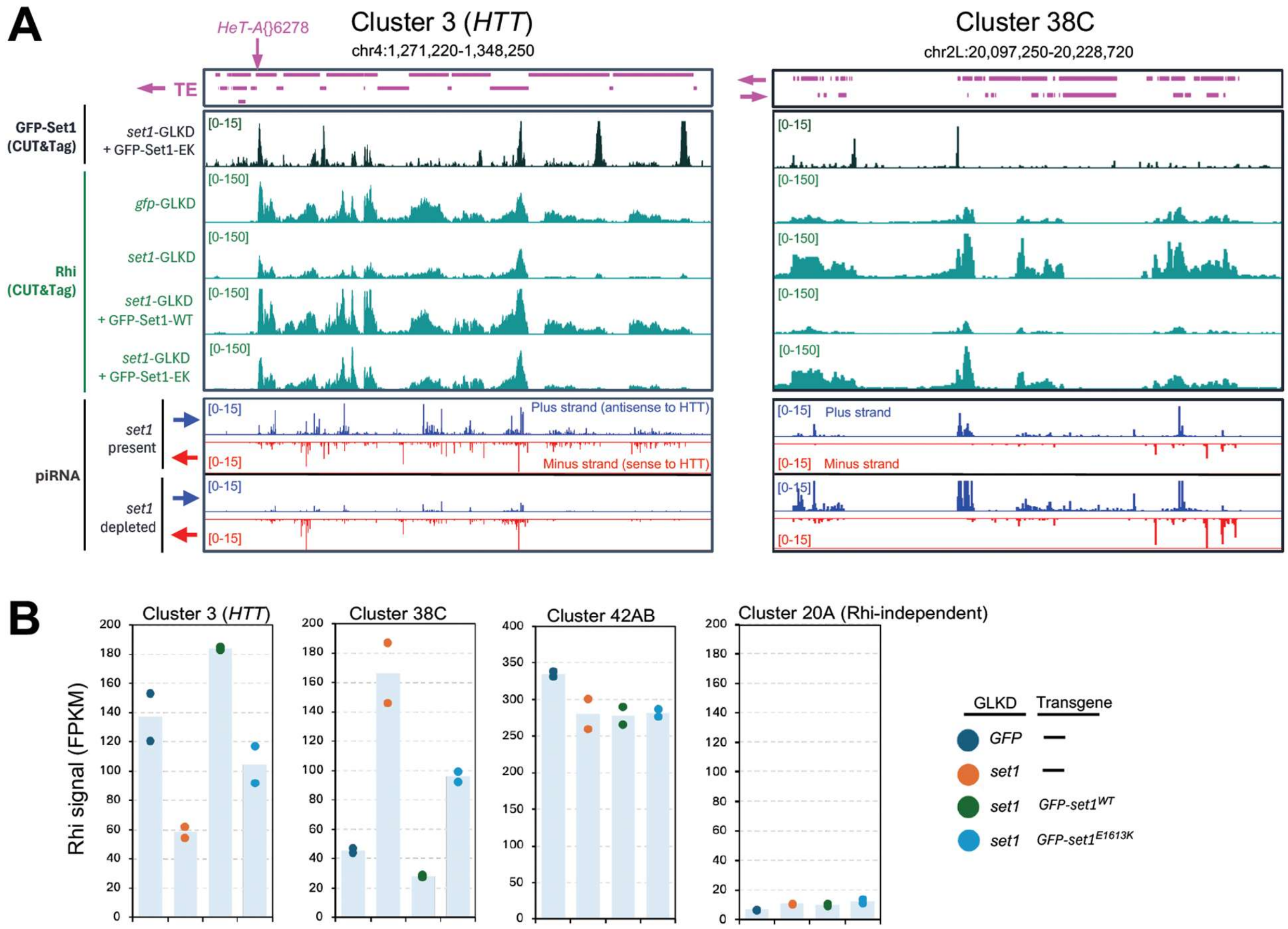

Figure 8. Effect of Set1 depletion on Rhi localization to piRNA cluster loci (A) Track view at subtelomeric cluster 3 or 38C locus. Light green: signals from anti-Rhi CUT&Tag. Dark green: GFP-Set1-EK signals in *set1*-GLKD (anti-GFP CUT&Tag). Blue and red: piRNAs mapped to genomic plus and minus strands, respectively. Mean CPM from biological replicates (n=2). (B) Quantification of anti-Rhi CUT&Tag signals. Bars: mean FPKM (fragments per kilobase per million mapped reads), dots: individual FPKM, from biological replicates (n=2).

To our surprise, we found that Rhi are overaccumulated at a major cluster locus, 38C, upon *set1*-GLKD. This is associated with piRNA overproduction from this locus. On the other hand, Rhi at 42AB was not strongly affected, but showed fluctuation within the locus (see attachment below, new Figure EV5AB). We further observed a more drastic local Rhi flucuation in subtelomeric region on chromosome 2R. There, Rhi is depleted on *HTT* repeat region while overaccumulated in the neighboring *invader4* repeats (new Figure EV5C, attached below).

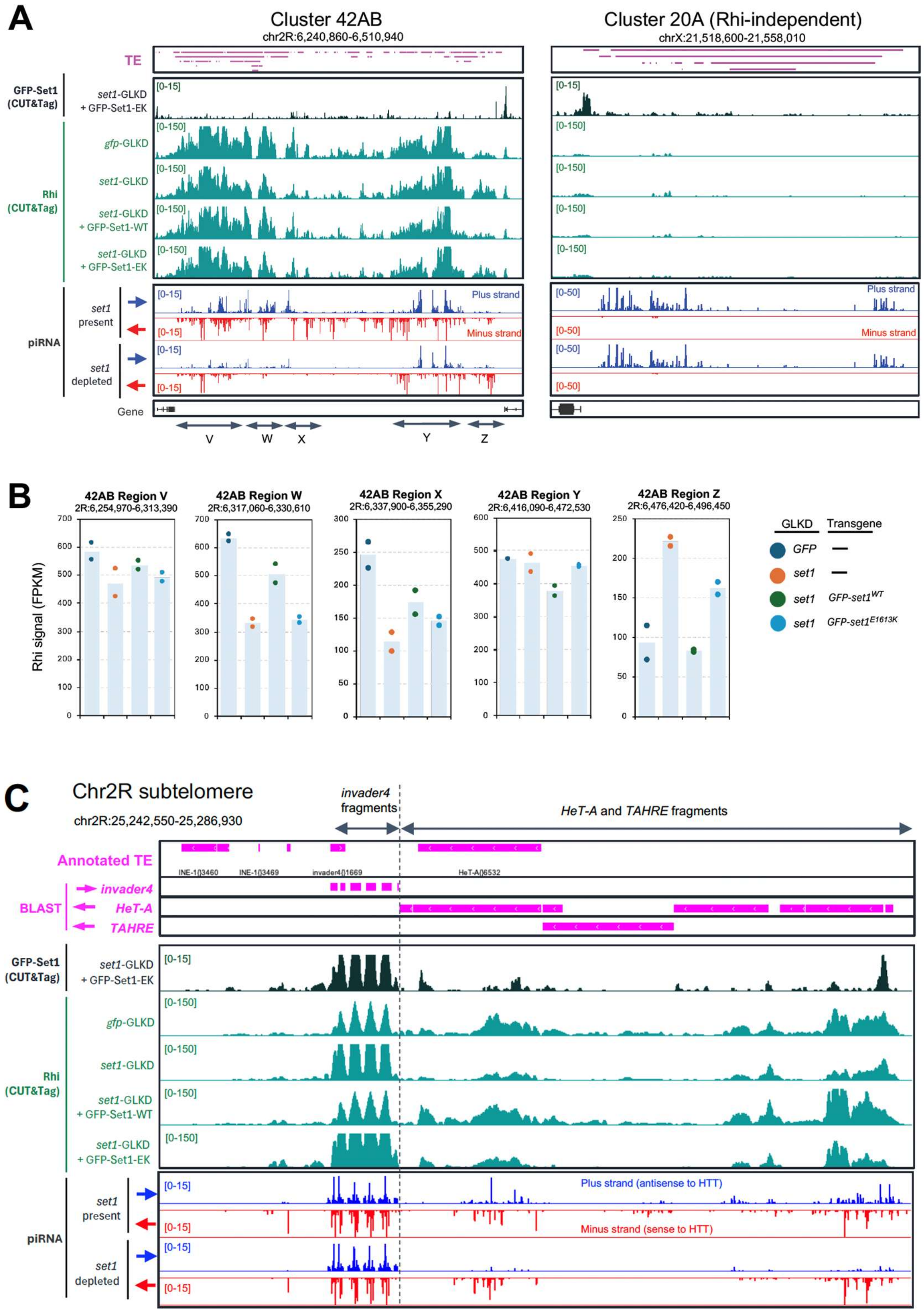

Figure EV5. Effect of Set1 depletion on Rhi localization at piRNA cluster loci, and effect of Piwi depletion on H3K9me3 deposition in subtelomeric cluster (A) Track view at 42AB or 20A locus. Light green: signals from anti-Rhi CUT&Tag. Dark green: GFP-Set1-EK signals (anti-GFP CUT&Tag). Blue and red: piRNAs mapped to genomic plus and minus strands, respectively. (B) Quantification of anti-Rhi CUT&Tag signals. Bars: mean FPKM, dots: individual FPKM, from biological replicates (n=2). Analyzed regions within 42AB are indicated in panel A. (C) Track view in the subtelomeric region on chromosome 2R.

Overall, our results revealed that Set1 is crucial for localizing Rhi to subtelomeric cluster loci. In addition, our results indicate that Set1 acts as a regulator of global Rhi distribution. This phenotype cannot be simply explained by reduced expression of Rhi and associated factors as a consequence of COMPASS dysfunction in *set1*-GLKD (Figure EV2). These results suggest that catalytic activity-independent, noncanonical Set1 functions underlies Piwi-mediated piRNA cluster formation and antisense-biased piRNA production.

New Figure 8 and Figure EV5 showing these anti-Rhi CUT&Tag data are included in our revised manuscript.

2: It remains unclear whether the **Piwi-Set1 interaction** is required for Set1-mediated telomere regulation.
Thank you for your critical comment. We still do not have a clear answer for this point. Given the physical interaction is weak (Figure 6A compared to EV3A), the direct interaction between the two may not be critical in telomere TE silencing. To investigate the interplay between Piwi and Set1 in subtelomere localization, we performed reciprocal CUT&Tag.

To examine whether Set1 localizes to subtelomere in a Piwi-dependent manner, we analyzed GFP-Set1-EK in *panx*-GLKD condition (We could not use *piwi*-GLKD, because both *set1* transgene and *piwi* short hairpin are inserted to attP2 on 3rd chromosome). As a result, *panx*-GLKD did not affect Set1 localization on subtelomeric cluster 3 (see below). We observed even stronger GFP-Set1-EK signals in *panx*-GLKD.

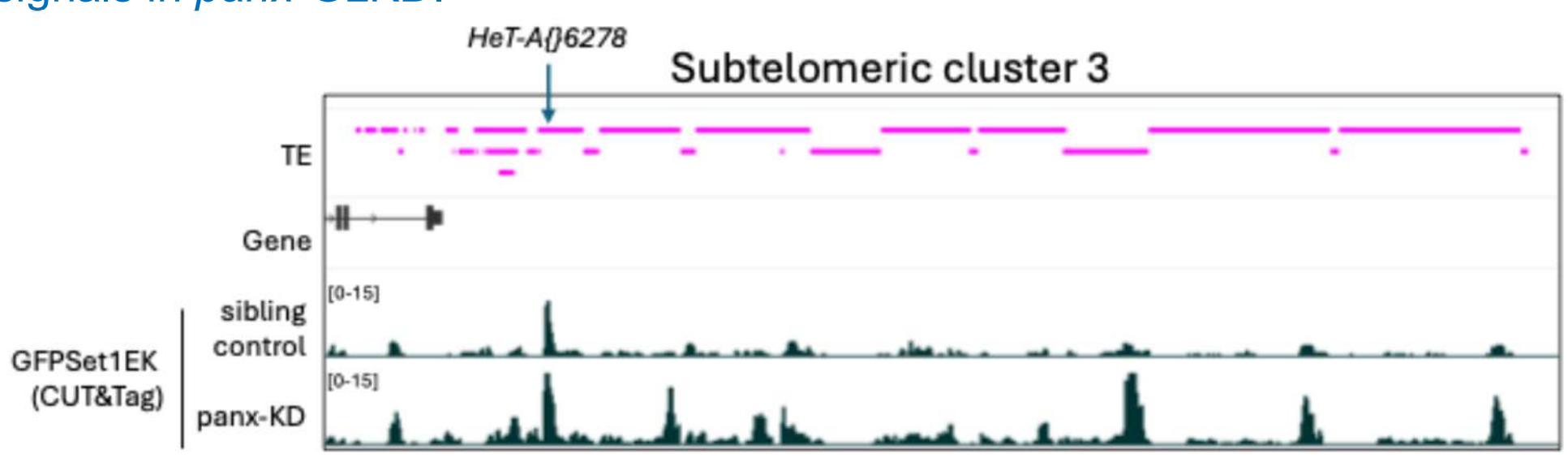

We consider that derepression of *HTT* might provide more accesibility by Set1 in *panx*-GLKD. To suppor this, we observed increased H3K4me3 at cluster 3 upon *HTT* depression in *set1*-GLKD (Figure 5). Our conclusion is that Set1 does not need Piwi to localize to subtelomere.

We next examined whether Piwi localizes to subtelomere in a Set1-dependent manner, by performing CUT&Tag for Piwi (rep1: anti-Piwi (SantaCruz), rep2: anti-GFP for GFP-Piwi) in *set1*-GLKD condition. As a result, we did not see the loss of Piwi signals upon *set1*-GLKD (see below).

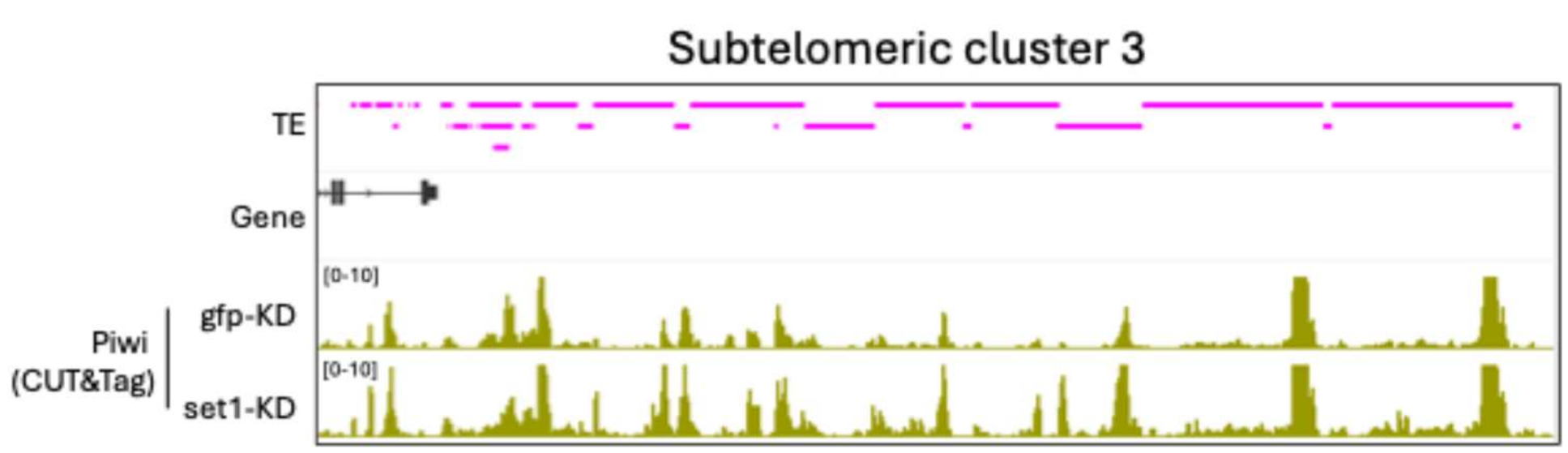

The results of reciprocal CUT&Tag were not included in the revised manuscript.

Related to your major point 1, we found that Set1 plays crucial roles in recruiting Rhi to subtelomere for piRNA cluster formation (new Figure 8). It has been reported that Piwi-mediated piRNA cluster

formation is initiated at embryonic stages (Akkouche et al., 2017). We re-analyzed their raw data for *piwi*-embKD (embryonic KD), and found that the subtelomere regions including cluster 3 requires Piwi to deposit H3K9me3 and form piRNA cluster (new Figure S8D, attached below).

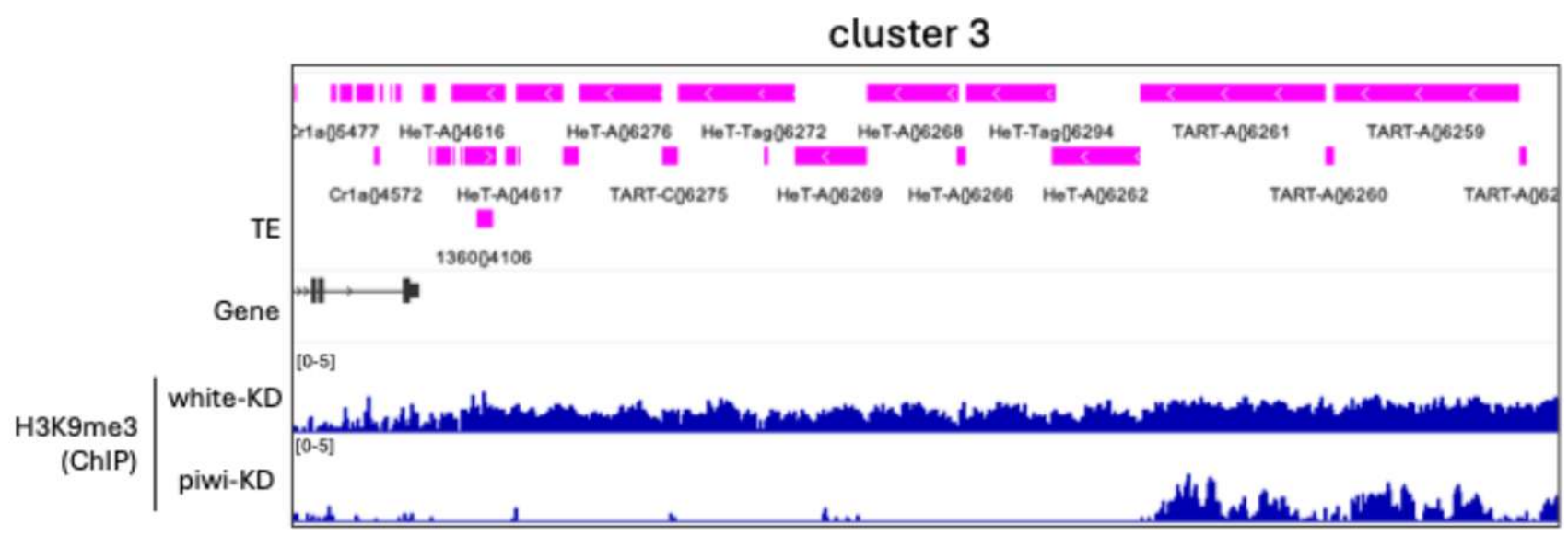

(D) Track view at subtelomeric cluster 3 showing H3K9me3 signal (mean CPM from biological replicates (n=2)). embKD: knockdown at embryonic stages. Raw data: GSE83238.

The above reciprocal CUT&Tag was conducted in ovaries at adult stage. It is possible that adult condition may not be able to address the possible interplay between Piwi and Set1 functioning at earlier developmental stages. It is technically challenging to conduct CUT&Tag in germline cells at embryonic stages.

Based on the analyses for TE transcripts, piRNA, Set1 and Rhi localization at adult stages (Figure 3-8), we propose a model in which Set1 binding to *HTT* sequences cooperates with Piwi to form piRNA clusters at subtelomeric loci (new Figure 8C, attached below). We continue our work to deepen the molecular insight into subtelomeric piRNA cluster formation.

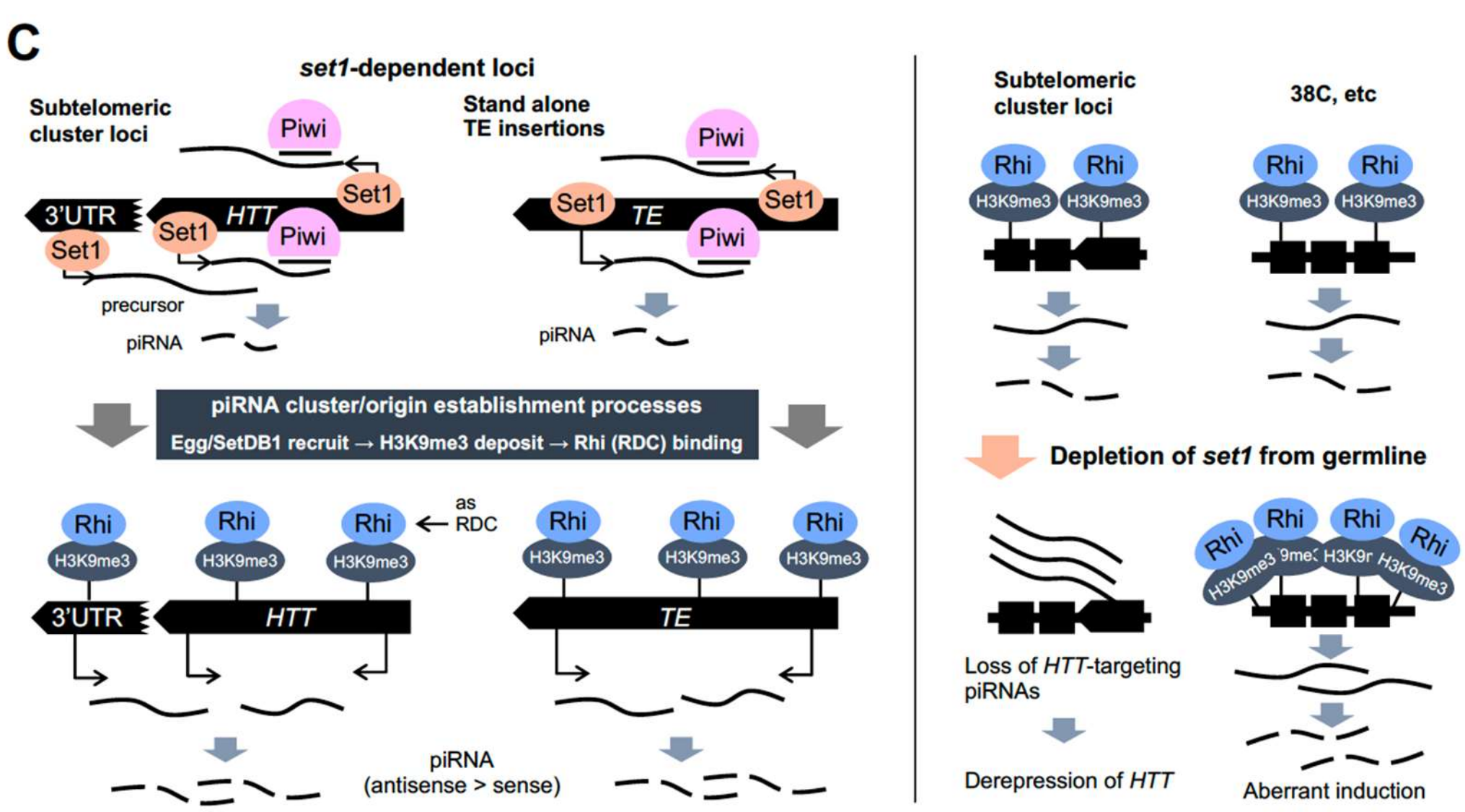

(C) Working model. Set1 interacting with *HTT* sequences cooperates with Piwi for piRNA cluster formation in subtelomeric regions. Set1 provides target templates for Piwi-piRNA complexes and/or precursor transcripts for piRNA production. Fragmented *HeT-A* 3'UTR retaining antisense TSS plays a crucial role in the latter process. Cooperation between Set1 and Piwi promotes H3K9me3 deposition and Rhi recruitment. Once this piRNA cluster status is established, Set1 and Piwi become dispensable. Similar mechanisms operate in the silencing of stand-alone TEs. Germline cells lacking the noncanonical functions of Set1 not only fail to establish piRNA clusters at subtelomeric loci, but also exhibit aberrant Rhi distribution and piRNA overproduction at certain loci.

We would like to note that Set1 was identified in Piwi proximity at adult stage, using germline stem cell (GSC) tumor condition. This might imply that GSC retains the ability to modulate piRNA cluster activities at certain loci. There are other factors in Piwi proximity remained uncharacterized due to the lack of short hairpin lines (some RNAi might not be effective enough). In addition, RNAi might not be effective enough for all. Hence, knock-out (KO) screening we are conducting now could identify novel factors interacting with Set1 and Piwi, which helps further deepen the mechanistic insight.

Minor concerns
Line 230: "immunotaining" should be corrected to "immunostaining."
Corrected. Thank you.

Last, we mention we added meta-profile for H3K4me3 and GFP-Set1 signals obtained by CUT&Tag in the revised manuscript. New Figure 5D for H3K4me3 CUT&Tag, and new Figure 7CE for GFP-Set1, as attached below. These results show the TSS-associated peaks formed by H3K4me3 and GFP-Set1. Set1 can show its enrichment on TE 3'UTRs with this heatmap, which is followed by track views on individual TE families.

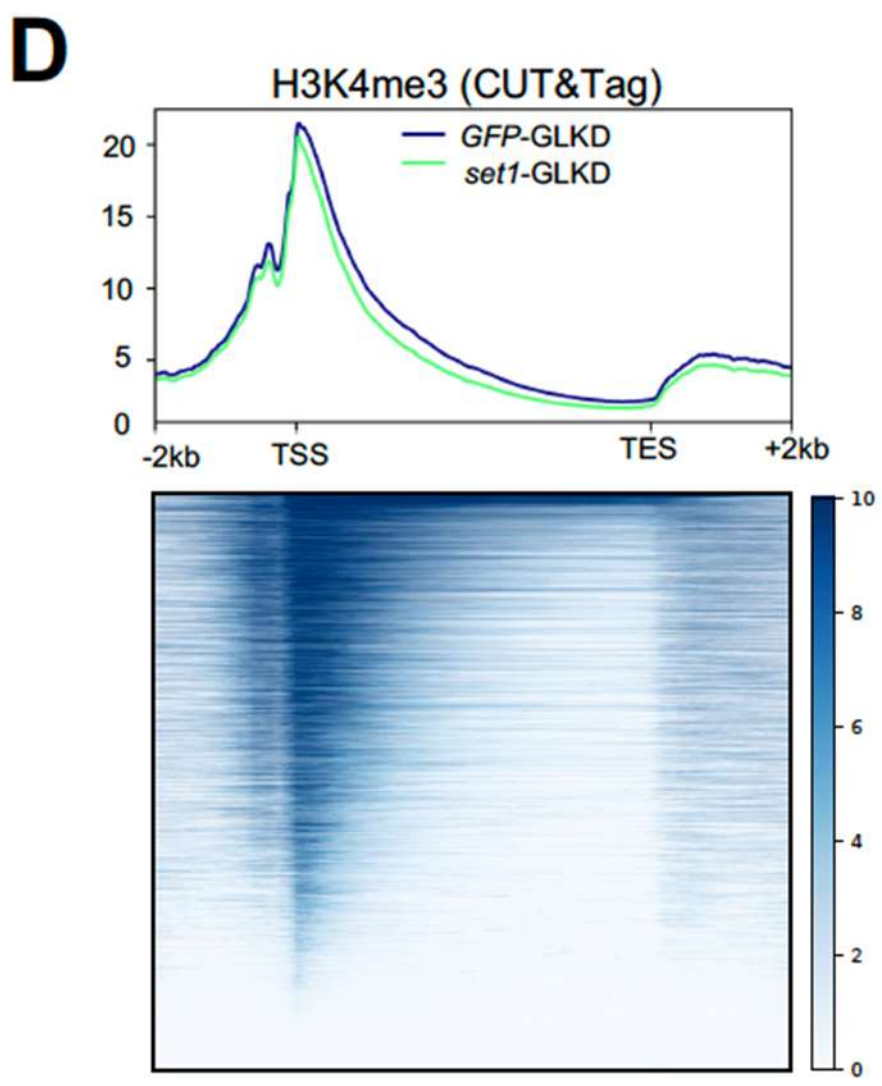

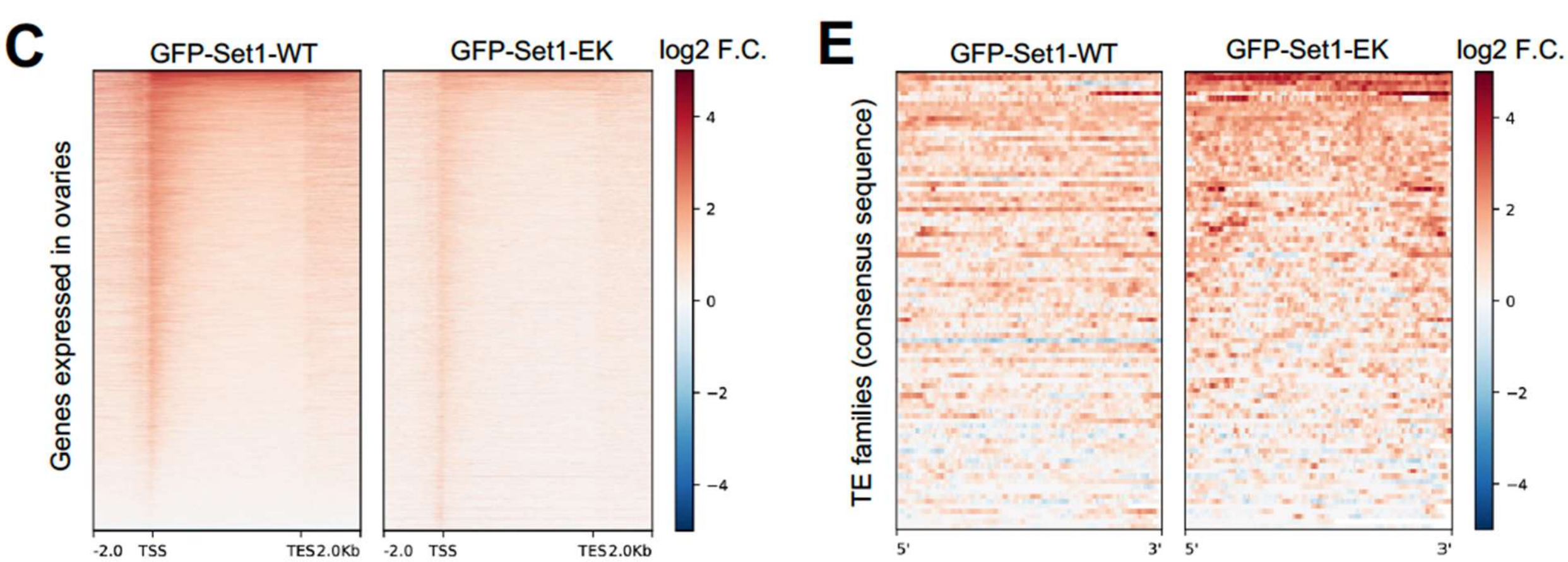

Referee #2:

Summary

To better understand how transposon silencing by the piRNA pathway is established, Isshiki and colleagues set out to characterize the proximal interactome of the PIWI-clade Argonaute proteins Piwi, Aubergine (Aub), and Ago3 in the Drosophila female germline. The authors employed nanos-driven TurboID fusion constructs of the respective PIWI proteins to biotinylate proximal factors. Due to technical challenges, they enriched for germline stem cell-like cells (GSCLCs) to achieve a robust pulldown of biotinylated targets. They then investigated the functional role of uncharacterized piRNA pathway candidates by testing the effect of their knockdown on Krimper localization as a proxy for nuage integrity and on HeT-A Gag protein accumulation as a readout of transposon derepression. Results of this validation screen prompted the authors to follow up on Hrb27C and Set1 as putative effectors of Aub- and Piwi-mediated silencing, respectively. Further experiments revealed that Hrb27C exerts TE repression in GSCLCs only upon bam depletion, with no detectable effect under standard knockdown conditions. The remainder of the manuscript focuses on Set1, an H3K4 histone methyltransferase required for TE silencing in germline cells. Rescue experiments with RNAi-resistant constructs demonstrate that a catalytic mutant of Set1 only partially rescues the silencing phenotype, indicating an H3K4 methylation-independent contribution of Set1. Profiling of piRNAs from Set1-depleted ovaries reveals a selective reduction in piRNAs mapping to telomeric transposable elements. Finally, CUT&TAG profiling of H3K4me3 and Set1 occupancy shows that wild-type Set1 binds most strongly at gene loci, whereas the catalytic-dead version displays a higher proportion of peaks overlapping with TE loci. Finally the authors show binding of Set1 to subtelomeric piRNA cluster 3, which contains truncated HeT-A 3' UTR insertions and suggest that Set1 promotes its piRNA precursor transcription.

Summary assessment

The manuscript reports an interesting observation linking Set1 to TE silencing and piRNA biology. However, some points remain incomplete (see below). The central concern is that the manuscript does not adequately address the potential effect of Set1 loss on Rhino, the well-established master regulator of piRNA cluster transcription in Drosophila ovaries. **Even a partial reduction in Rhino activity**, which might not lead to detectable nuage disassembly phenotype, could account for the observed silencing defects. While the manuscript provides an interesting observation of Set1's role in transposon silencing the points should be addressed before publication.

Thank you very much for your constructive comments. Following your advises, we were able to provide new results and improved our manuscript. Essentially, newly generated Figure 8 and Figure EV5 demonstrate that Set1 is crucial in recruiting Rhi to subtelomeric cluster loci for piRNA production.

We unexpectedly found that *set1*-GLKD has an opposite effect at 38C locus (one of well-characterized Rhi-dependent cluster locus). At 38C, Rhi were overaccumulated and piRNAs were overproduced.

The global Rhi redistribution phenotype (loss at subtelomeric loci, gain at other loci) is unlikely to be a mere consequence of reduced Rhi levels in Set1-COMPASS deficiency.

In addition, we show that Set1 catalytic mutant (EK variant) which does not restore *rhi* expression maintains the ability to recruit Rhi to subtelomeric cluster loci (similar to *HTT* rerepression activity). This result would discriminate the function to activate subtelomeric piRNA cluster loci from that as COMPASS in gene activation. These results further support the notion that Set1 plays a noncanonical role, acting with Piwi for piRNA production and TE silencing in telomere.

Major points

1. Relationship between Set1 and Rhino-dependent piRNA cluster regulation

The potential impact of Set1 on Rhino biology is insufficiently adressed in the manuscript. Given that Rhino is a well established master regulator of dual-strand piRNA clusters, it is critical to assess

whether Set1 depletion affects Rhino localization or occupancy at piRNA clusters. The authors should include **ChIP-seq profiling of Rhino in Set1-depleted ovaries**. In addition, comparison of the observed **piRNA** and **TE derepression** phenotypes with published datasets from mutants of Rhino-associated factors (**Rhino, Deadlock, Cutoff**, **Moonshiner**, **Nxf3, Kipferl,** and others) would substantially strengthen the interpretation and help to place a **Set1-specific effect** from a general Rhino pathway perturbation.

Thank you very much for your critical suggestion. We re-analyzed public poly(A) transcriptome datasets from *rhino, deadlock, cutoff, nxf2, nxf3, boot, kipferl*, and *e(z)* mutants, and found Rhi (and Nxf2, Boot) is crucial in silencing *HTT* (see below, new Figure 4D and new Figure S4E). Of note, we did not include the results from libraries using rRNA depletion, because *HTT* derepression is only mild even in *rhi* KO (*moonshiner* KO too) in the dataset (GSE97719).

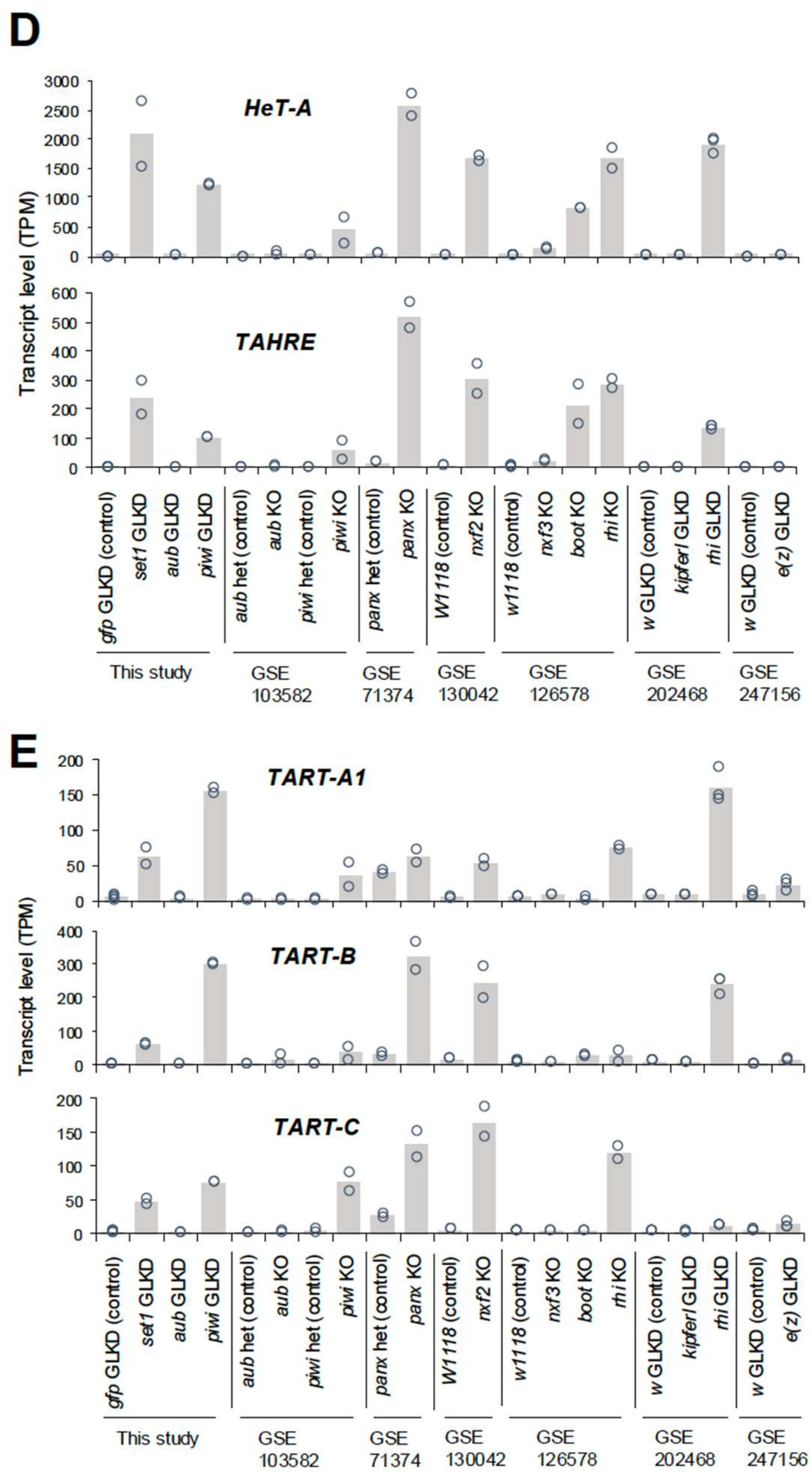

In addition to transcriptome data, we re-analyzed public piRNA-seq data from *rhi, nxf3, boot* mutants showing robust *HTT* derepression (see below, new Figure 6E and new Figure EV3C). These results show that Rhi, Nxf3, Boot are required for piRNA production for broad range of TE families including *HTT*. This global effect is contrastive to *HTT*-biased requirement of Set1 and Panx.

E

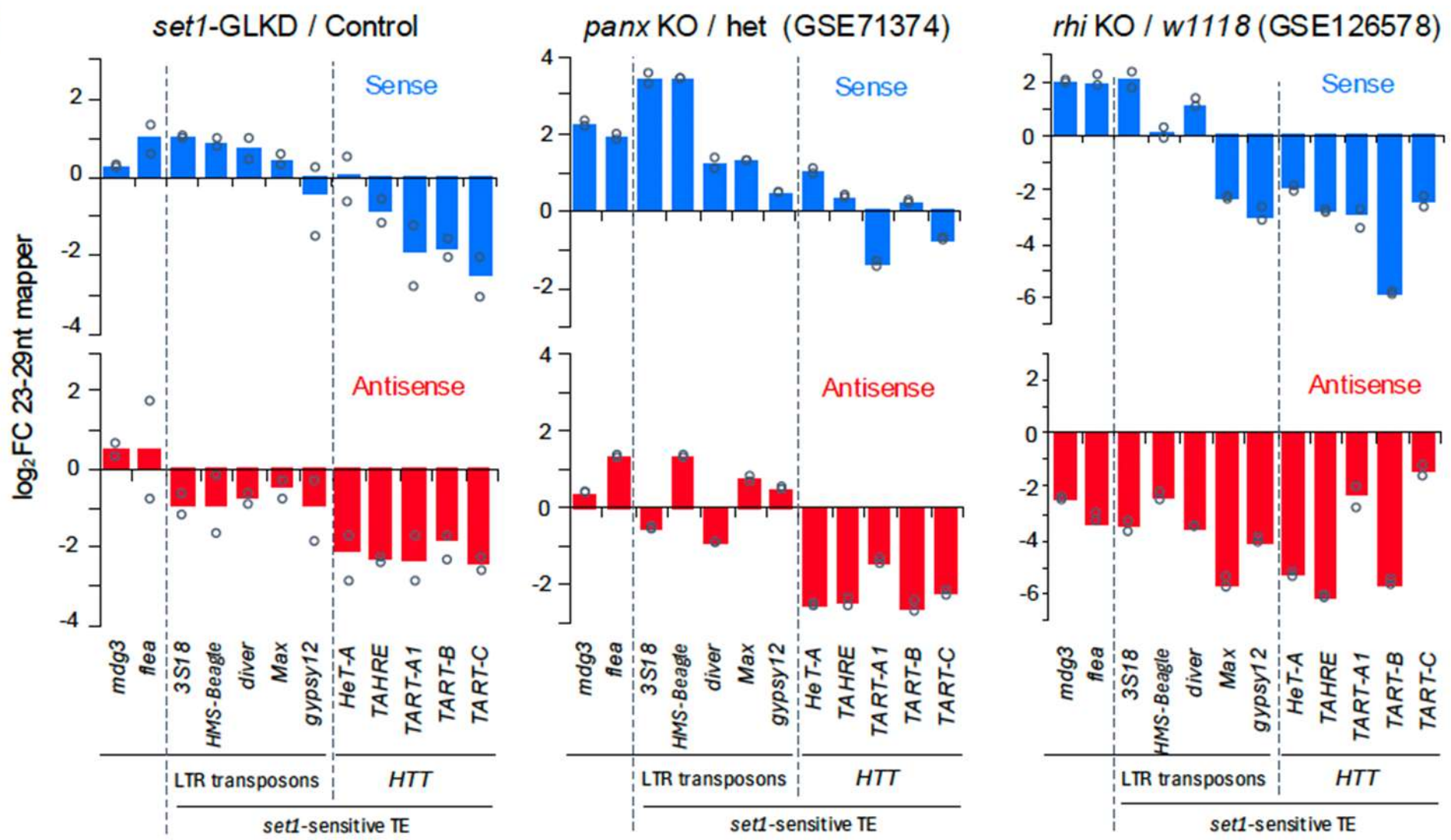

C

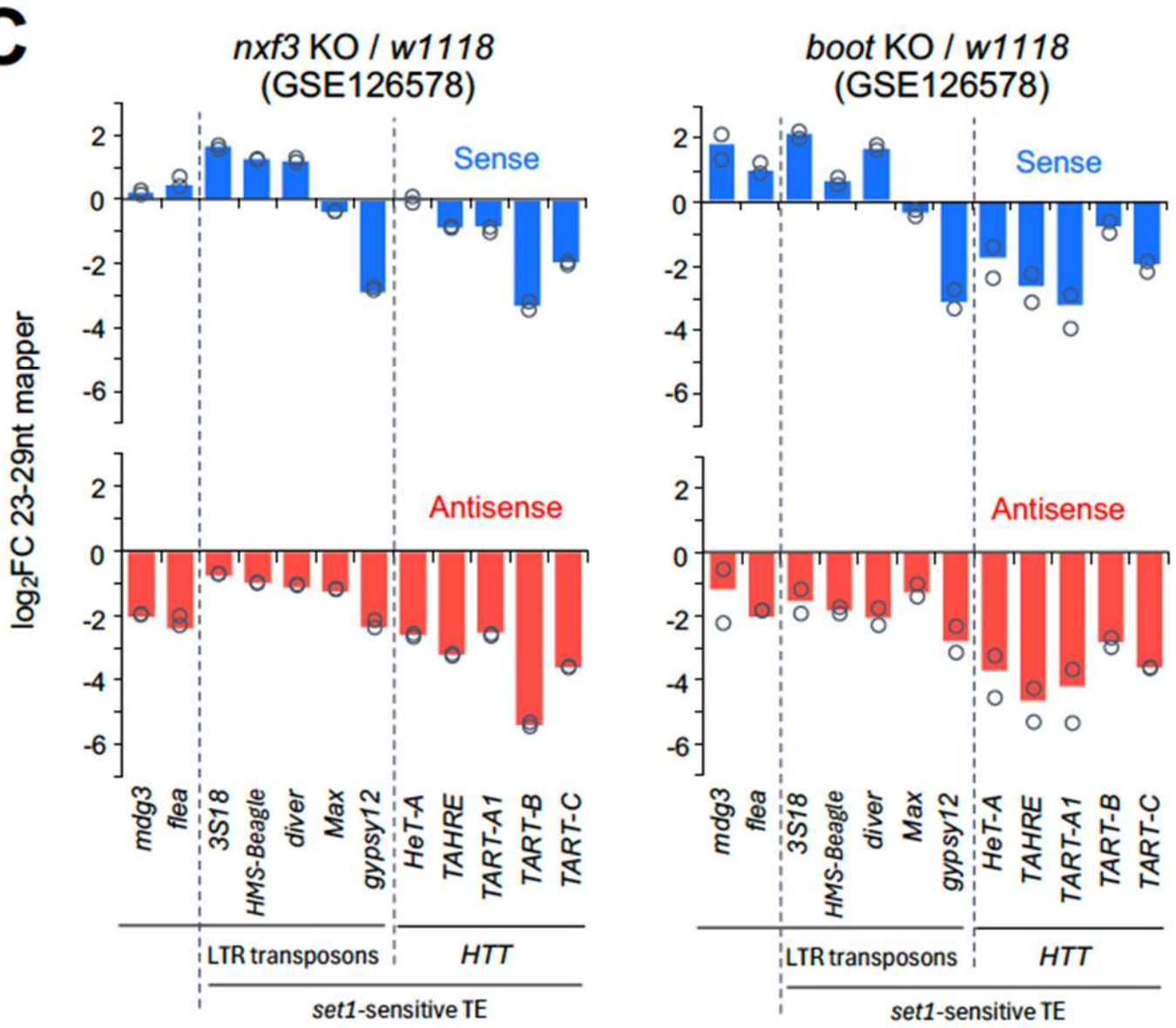

To examine how Set1 and Rhi interact in *HTT*-targeting piRNA production, we analyzed Rhi localization genome-widely by CUT&Tag using anti-Rhi antibodies. This antibody has been used for Rhi ChIP-seq (Mohn et al., 2014).

First, we observed Rhi accumulation at major piRNA cluster loci (42AB, 38C) as well as at subtelomeric cluster 3 locus. It was confirmed that Rhi is absent in 20A locus whose piRNA production is Rhi-independent.

Next, Rhi signals were compared between the four conditions; (1) control *gfp*-GLKD (germline knockdown), (2) *set1*-GLKD, (3) *set1*-GLKD expressing GFP-Set1-WT, and (4) *set1*-GLKD expressing GFP-Set1-EK (catalytic mutant).

We found striking reduction in Rhi signals upon *set1*-GLKD at subtelomeric cluster 3 (see attachment below, new Figure 8). Rhi level was fully restored with GFP-Set1-WT, and less efficiently with GFP-Set1-EK. The pattern is similar to what we observed in TE rerepression (Figure 5, full rerepression by GFP-Set1-WT and less efficient rerepression by GFP-Set1-EK). These results suggest that Set1 is required for Rhi accumulation at subtelomeric cluster loci for piRNA production.

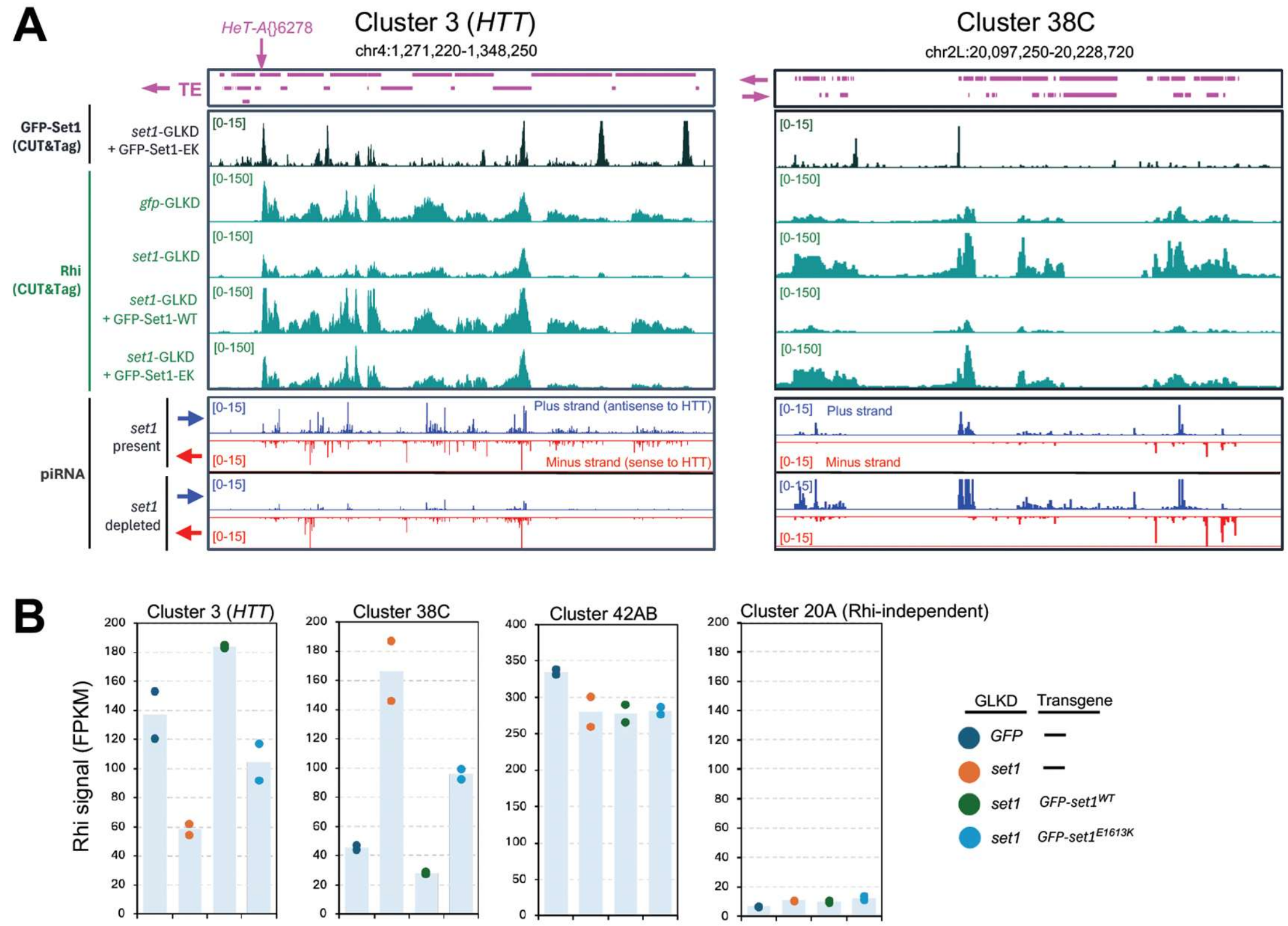

Figure 8. Effect of Set1 depletion on Rhi localization to piRNA cluster loci (A) Track view at subtelomeric cluster 3 or 38C locus. Light green: signals from anti-Rhi CUT&Tag. Dark green: GFP-Set1-EK signals in *set1*-GLKD (anti-GFP CUT&Tag). Blue and red: piRNAs mapped to genomic plus and minus strands, respectively. Mean CPM from biological replicates (n=2). (B) Quantification of anti-Rhi CUT&Tag signals. Bars: mean FPKM (fragments per kilobase per million mapped reads), dots: individual FPKM, from biological replicates (n=2).

Moreover, we unexpectedly found that Rhi is overaccumulated at a major cluster 38C upon *set1*-GLKD, which is associated with piRNA overproduction from there. On the other hand, Rhi at 42AB was not clearly affected overall, but showed local fluctuation within 42AB (see attachment below, new Figure EV5AB). We further observed a more drastic local Rhi flucuation in subtelomeric region on chromosome 2R. There, Rhi is depleted on *HTT* repeats while overaccumulated in the neighboring *invader4* repeats (new Figure S8C, attached below).

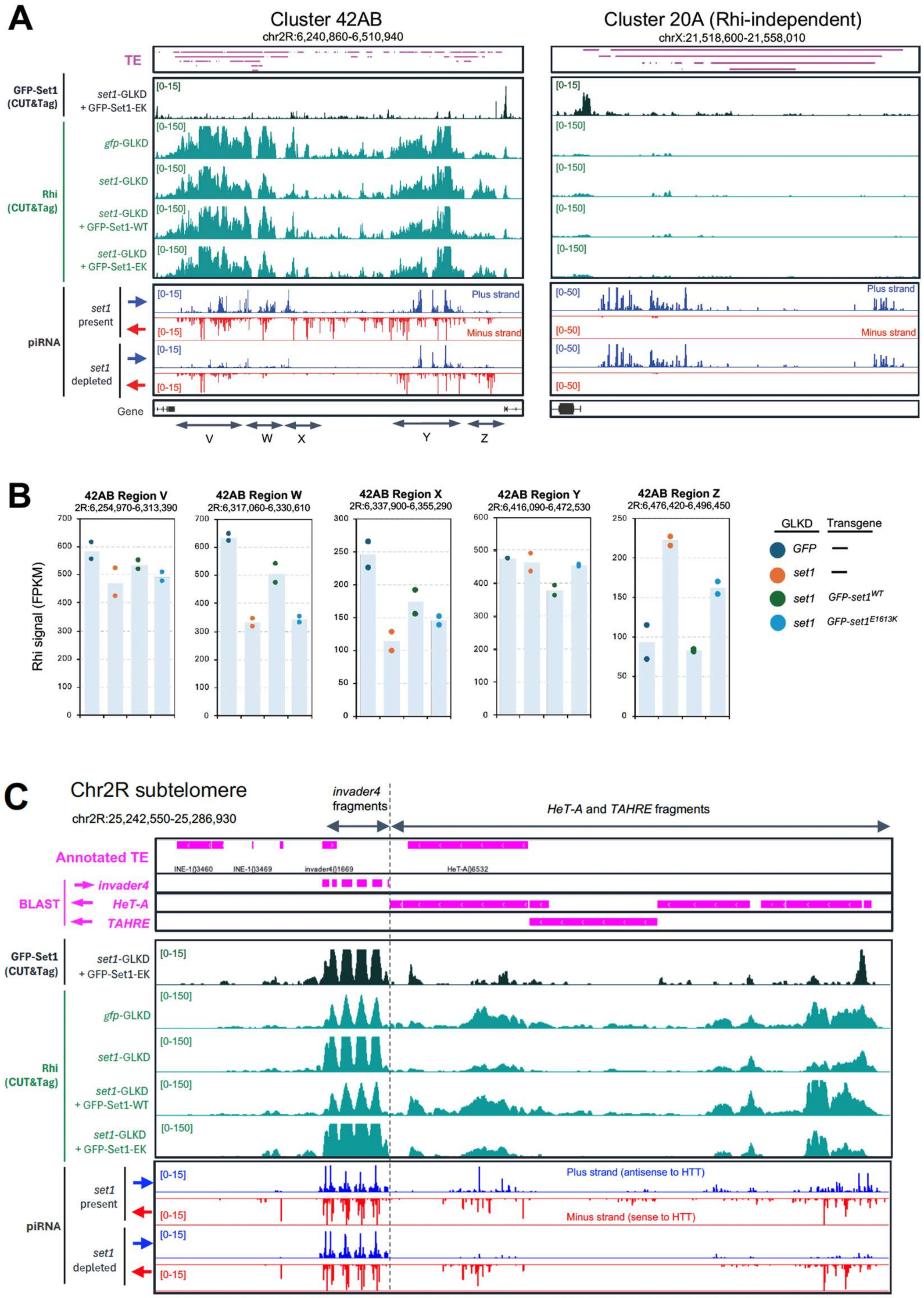

Figure EV5. Effect of Set1 depletion on Rhi localization at piRNA cluster loci, and effect of Piwi depletion on H3K9me3 deposition in subtelomeric cluster (A) Track view at 42AB or 20A locus. Light green: signals from anti-Rhi CUT&Tag. Dark green: GFP-Set1-EK signals (anti-GFP CUT&Tag). Blue and red: piRNAs mapped to genomic plus and minus strands, respectively. (B) Quantification of anti-Rhi CUT&Tag signals. Bars: mean FPKM, dots: individual FPKM, from biological replicates (n=2). Analyzed regions within 42AB are indicated in panel A. (C) Track view in the subtelomeric region on chromosome 2R. (D) Track view at subtelomeric cluster 3 showing H3K9me3 signal (mean CPM from biological replicates (n=2)). embKD: knockdown at embryonic stages. Raw data: GSE83238.

Overall, Set1 is crucial for Rhi localization on subtelomeric cluster loci. In addition, Set1 controls global Rhi distribution. The Rhi phenotype we observed in *set1*-GLKD will not be explained as a mere consequence of COMPASS dysfunction (Figure EV2, attached later as response to your point 3).

It has been reported that piRNA cluster formation is mediated by Piwi at embryonic stages (Akkouche et al., 2017). We re-analyzed their raw data (H3K9me3 ChIP-seq) and confirmed that subtelomere is sensisitve to embryonic Piwi knockdown (new Figure EV5D, attached below).

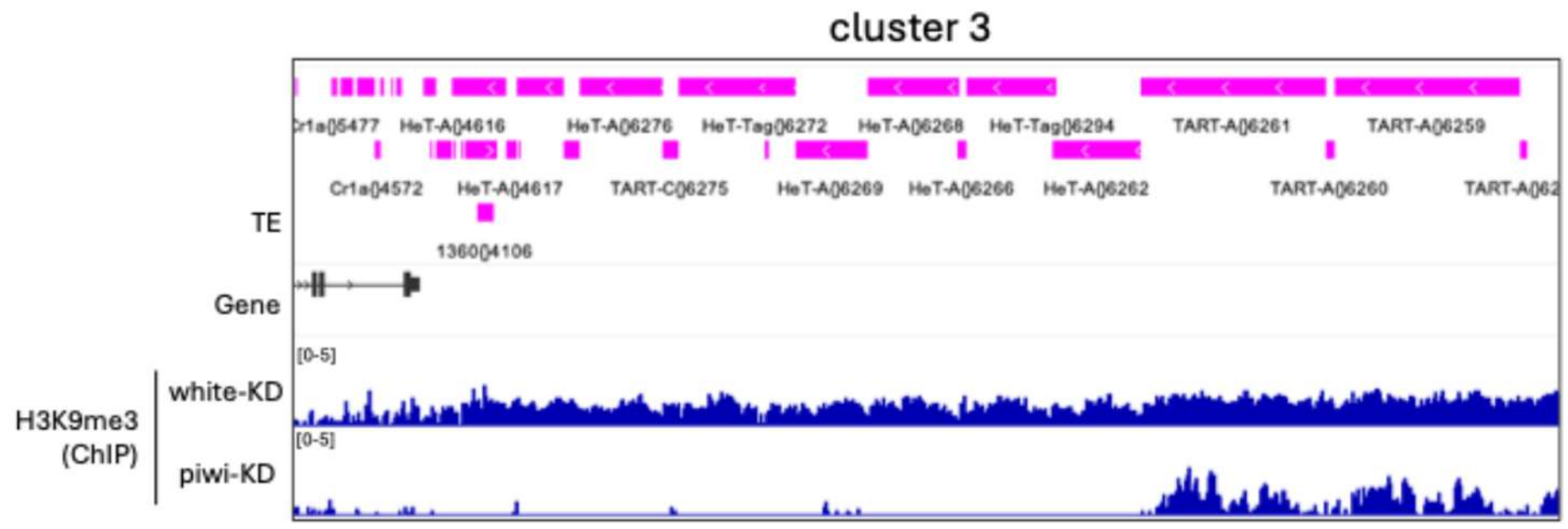

(D) Track view at subtelomeric cluster 3 showing H3K9me3 signal (mean CPM from biological replicates (n=2)). embKD: knockdown at embryonic stages. Raw data: GSE83238.

Taken together, we propose a model in which Set1 binding to *HTT* cooperates with Piwi to mediate piRNA cluster formation in subtelomeric regions (new Figure 8C, attached below).

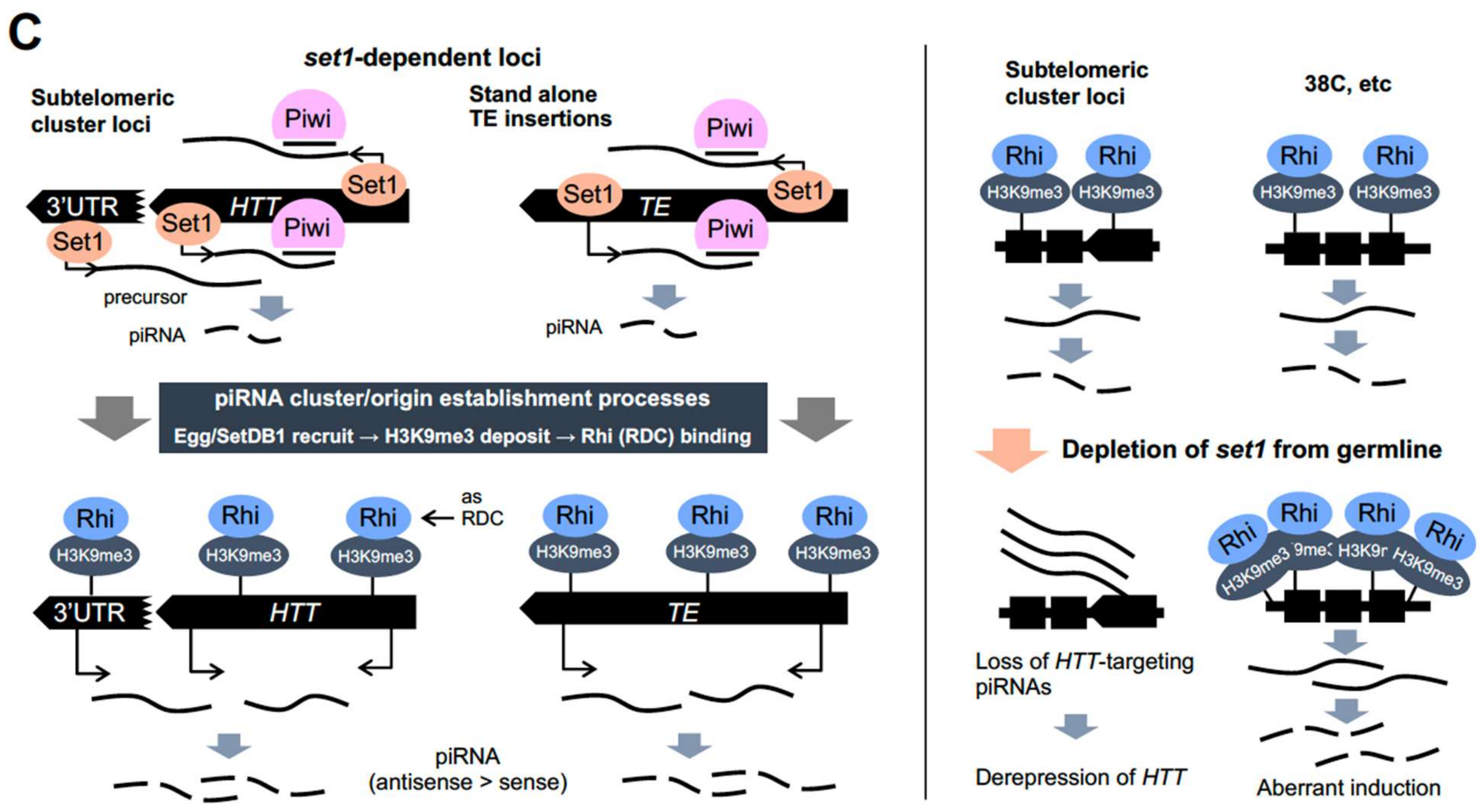

(C) Working model. Set1 interacting with *HTT* sequences cooperates with Piwi for piRNA cluster formation in subtelomeric regions. Set1 provides target templates for Piwi-piRNA complexes and/or precursor transcripts for piRNA production. Fragmented *HeT-A* 3'UTR retaining antisense TSS plays a crucial role in the latter process. Cooperation between Set1 and Piwi promotes H3K9me3 deposition and Rhi recruitment. Once this piRNA cluster status is established, Set1 and Piwi become dispensable. Similar mechanisms operate in the silencing of stand-alone TEs. Germline cells lacking the noncanonical functions of Set1 not only fail to establish piRNA clusters at subtelomeric loci, but also exhibit aberrant Rhi distribution and piRNA overproduction at certain loci.

2. Transcriptomic characterization of GFP-Set1WT versus GFP-Set1E1613K
An RNA-seq **comparison between GFP-Set1WT and GFP-Set1E1613K** expressing cells is

currently lacking. **MA plots or volcano plots** distinguishing effects on protein-coding genes from effects on TEs would allow a more rigorous assessment of the catalytic versus non-catalytic contributions of Set1 to the observed phenotypes and would directly support the rescue experiment interpretation. The comparison to a full depletion of Set1 and the different rescue constructs might help the interpretation of the catalytic mutant phenotype.

Thank you very much for your advise. Following the advise, we generated the volcano plot comparing WT and EK rescue conditions (see below). However, we think the plot is not very assisting to visualize the non-catalytic contributions of Set1 in TE silencing. This is due to the less efficient rescue effect exhibited by EK variant. The reason why EK acts less efficiently than WT is uncertain. Weak expression of GFP-Set1-EK compared to GFP-Set1-WT could have contributed (Figure 5B). Lack of canonical, catalytic activity-dependent function as COMPASS could also influence.

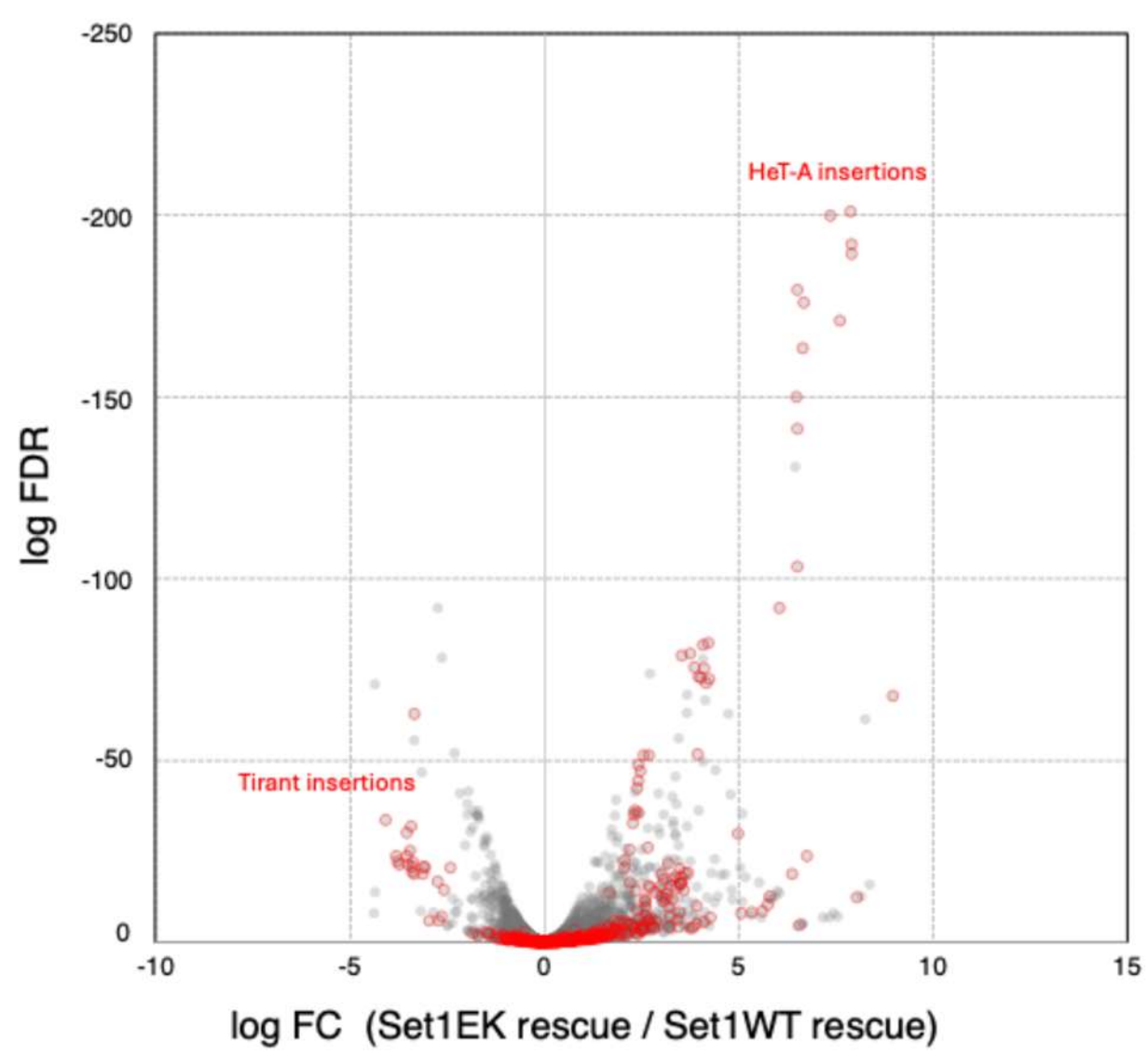

Since the plot comparing WT and EK rescues would not be helpful for readers, we prefer to show the bar graph (mean TPM) with dots (individual TPM from biological replicates) to show the contribution of catalytic activity-independent Set1 function (revised Figure 5C, attached below).

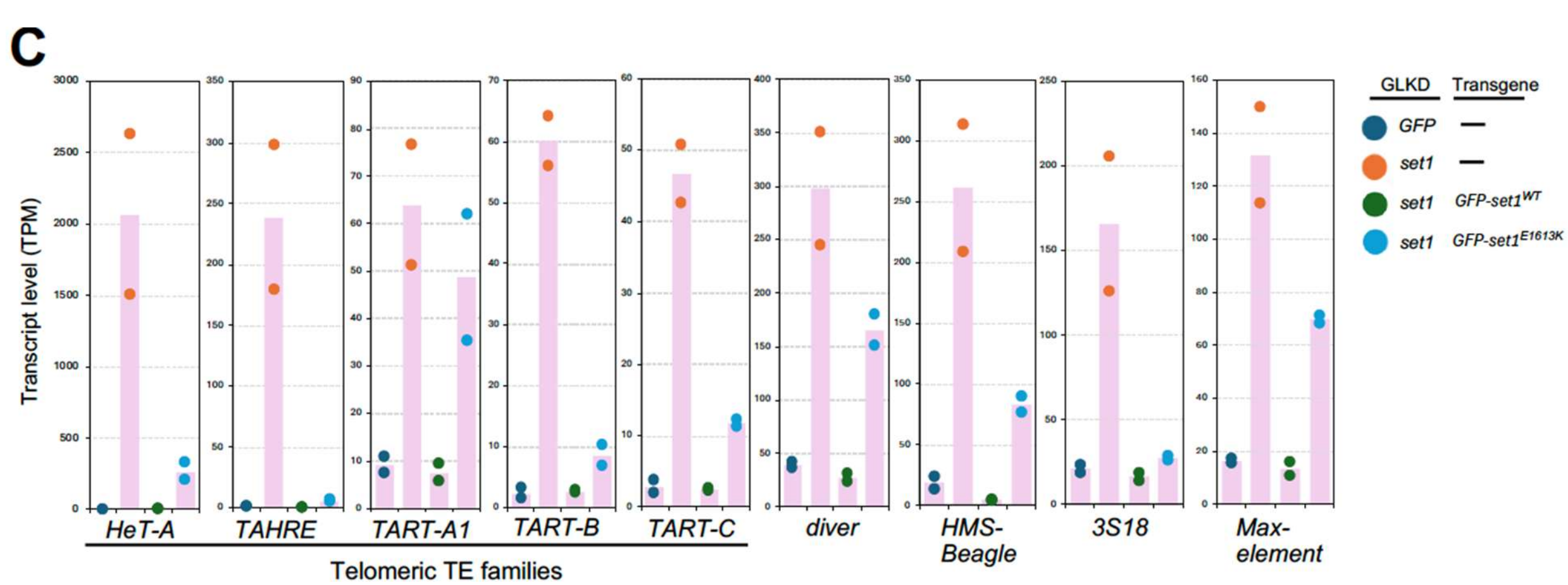

3. Incompleteness of the piRNA pathway factor list in Figure S5A

The piRNA pathway factor list used for the validation screen (Figure S5A) is incomplete. Notably,

multiple Rhino-associated factors are absent. This is a significant gap given the central relevance of Rhino biology in piRNA cluster transcription and therefore to the manuscript's conclusions.

Thank you very much for pointing this out. Indeed, our list was incomplete. Rhi, the associated factors, and downstream components are now included in the new Figure EV2A and EV2B (H3K4me3 signals and mRNA levels, respectively) in our revised manuscript (attached below). Our conclusion did not change. H3K4me3 signals are more sensitive to *set1*-GLKD, compared to steady state mRNA levels. Catalytic activity of E1613K was undetectable. However, E1613K retained the ability to recruit Rhi (Figure 8AB) and rerepress *HTT* (Figure 5C), supporting the noncanonical function of Set1.

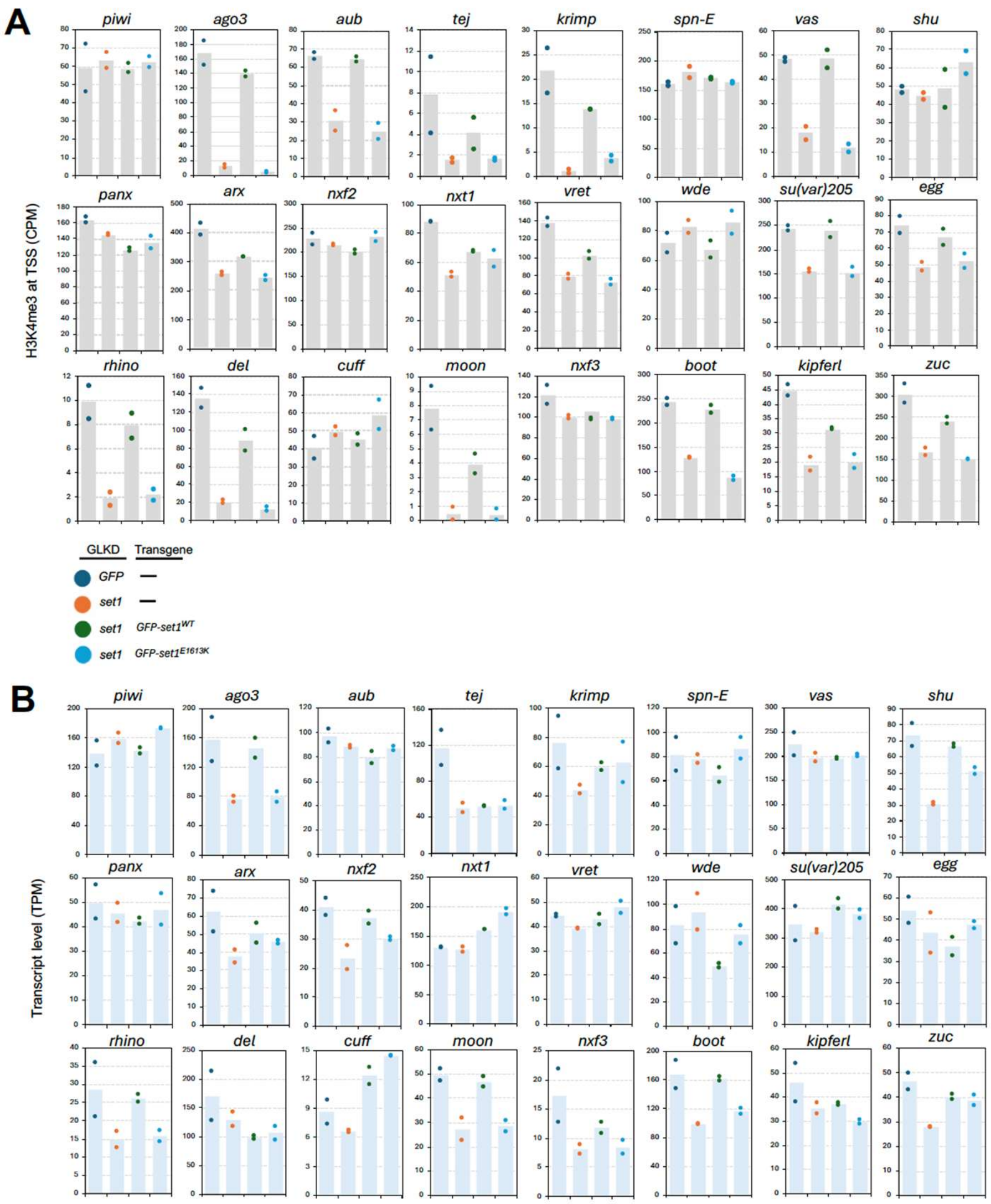

Figure EV2. Effect of *set1*-GLKD on the expression of piRNA pathway factors, and effect of COMPASS subunit GLKD on *HeT-A* expression (A) Bar graph showing mean H3K4me3 signal (RPM) around TSS (±500bp) of piRNA pathway genes. Dots: individual RPM from biological replicates (n=2). (B) Bar graph showing mean transcript levels (TPM). Dots: individual TPM from biological replicates (n=2).

4. Hrb27C data are too preliminary to be conclusive

The Hrb27C data currently show TE derepression only in the context of bam depletion in GSCLCs, with no detectable effect under standard knockdown conditions. Without demonstrating that TEs are affected in bona fide GSCs under non-bam-depletion conditions, for example by **RNA FISH,** the significance of this finding is unclear. The authors should either provide this additional evidence or

consider removing the Hrb27C section, as it does not substantially advance the manuscript's main conclusions in its current form.

Thank you for your comment. We agree that our data regarding hrb27C is preliminary to give a conclusion for this factor. However, from proteome view, Aub-TurboID identifying Hrb27C in cytoplasm is complementary to Piwi-TurboID identifying Set1 in nucleus. Hence, the identification of Hrb27C together with Set1 is supporting the advances of the proximity proteome approach we employed. Therefore, we would like to keep the data for Hrb27C in this manuscript. Nonetheless, we would like to discuss about this issue if hrb27C data removal is necessary.

Single molecule RNA FISH in GSC and other experiments are very important experiments to characterize Hrb27C, and will be performed in future study. Thank you very much again for your creative advise.

Figures and presentation:

1. Immunofluorescence image presentation:

All IF images should include **single-channel** views alongside the merged panels to allow proper assessment of signal specificity and colocalization (affected figures: **Fig. 1B**, Fig. 5B).

Thank you for your suggestion. We provide single-channel views in the new Figure 1B (attached below). On the other hand, in Figure 5B, we are not sure if single-channel views help the readers (attached below, but currently not included in the revised manuscript). Vas serves as a marker of germline cells. Only with Vas, loss of germline H3K4me3 signals can be recognized. But, we consider adding single channel images in Figure 5, if necessary.

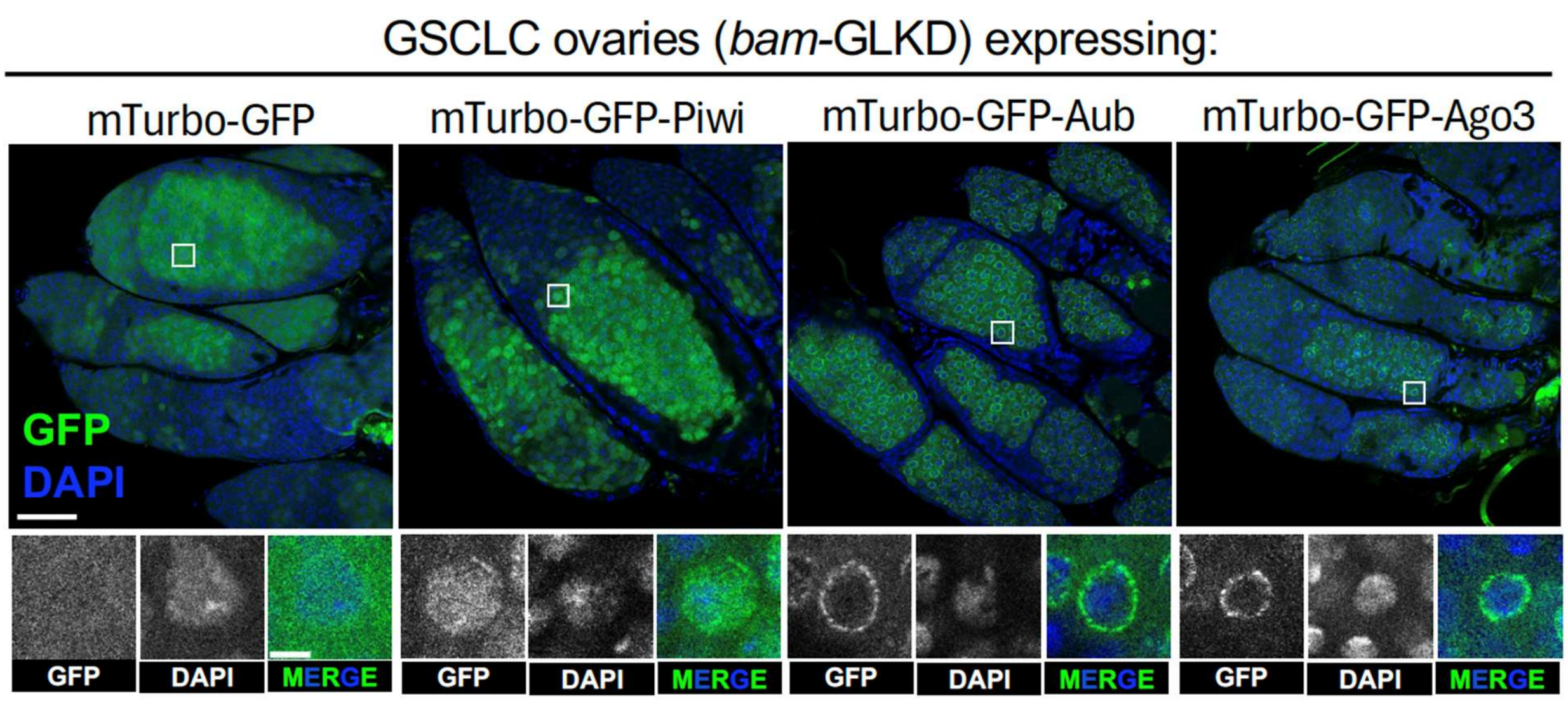

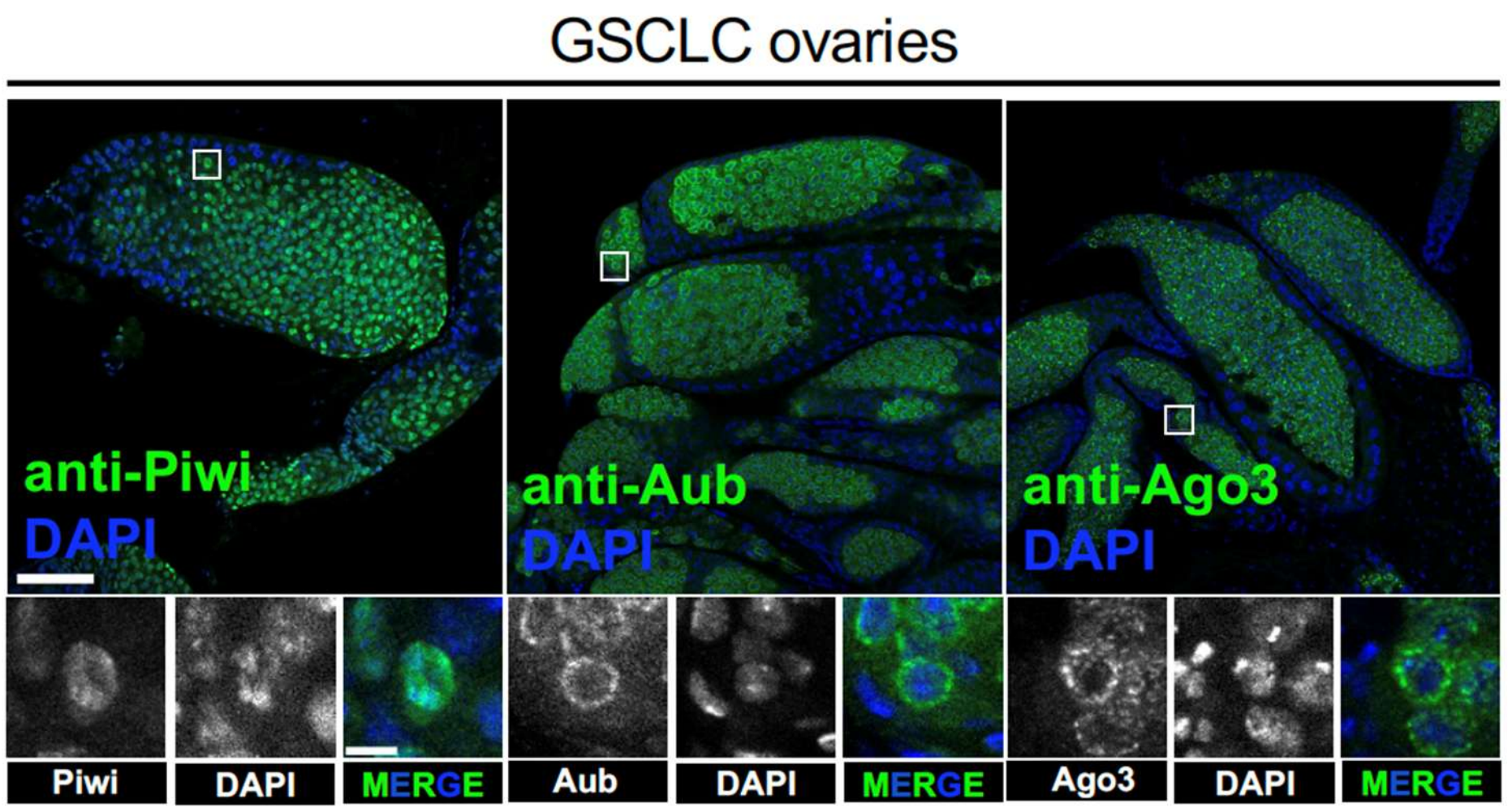
GSCLC ovaries
anti-Piwi
DAPI
anti-Aub
DAPI
anti-Ago3
DAPI
Piwi
DAPI
MERGE
Aub
DAPI
MERGE
Ago3
DAPI
MERGE

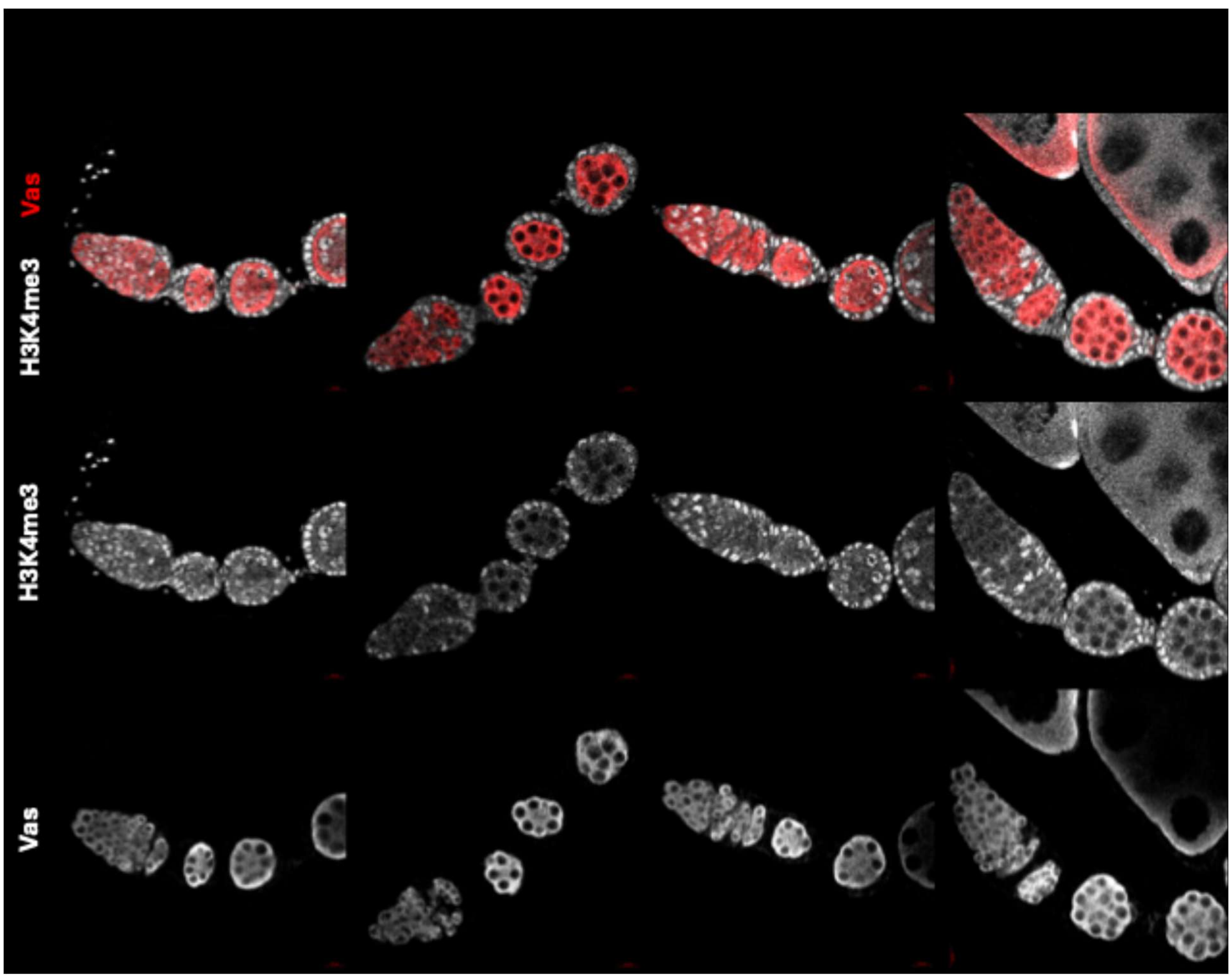
H3K4me3 Vas
H3K4me3
Vas

2. Mass spectrometry data (Figure 2A):
The presentation of the mass spectrometry data should be expanded by having volcano plots of the **combined replicates**. This will allow the readers to judge the **statistical power** and **log2FC** of the individual hits.
Thank you for your suggestion. We provide volcano plots of the combined replicates in the new Figure 2A (attached below). We further upgraded Figure S2 by highlighting known piRNA pathway components.

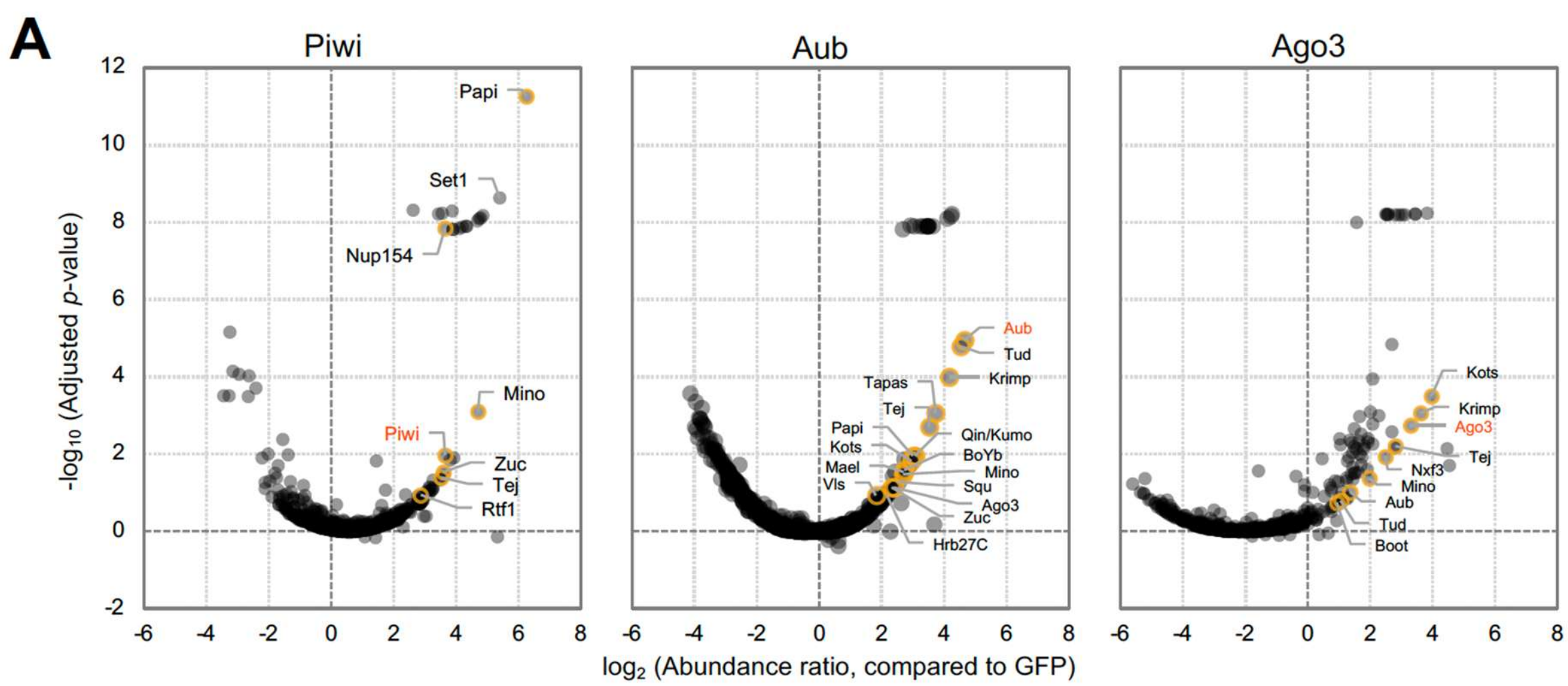

3. Comparison of GLKD screen results to published germline TE regulation data: The results of the germline knockdown (GLKD) screen should be compared to published genome-wide RNAi screen data for germline TE regulation, which would provide an orthogonal functional readout complementary to the IF-based scoring used here (Czech et al., 2013, Mol Cell 50:749-61; PMID: 23665227).
Thank you for your advise. We provide the information from Czech et al., 2013 in the revised Table EV3.

4. Figure S3A
The quantification of the localization phenotypes is hard to distinguish as all measured proteins have the same color (green). It would help the readers to associated them if different colors are used.
Thank you for your advise. It is tough to change the colors from now to all these measurement output. Appendix Figure S3ABC have clear indications for Krimp, Aub, and Piwi measured in the individual panels (attached below). We believe these are friendly enough for the readers.

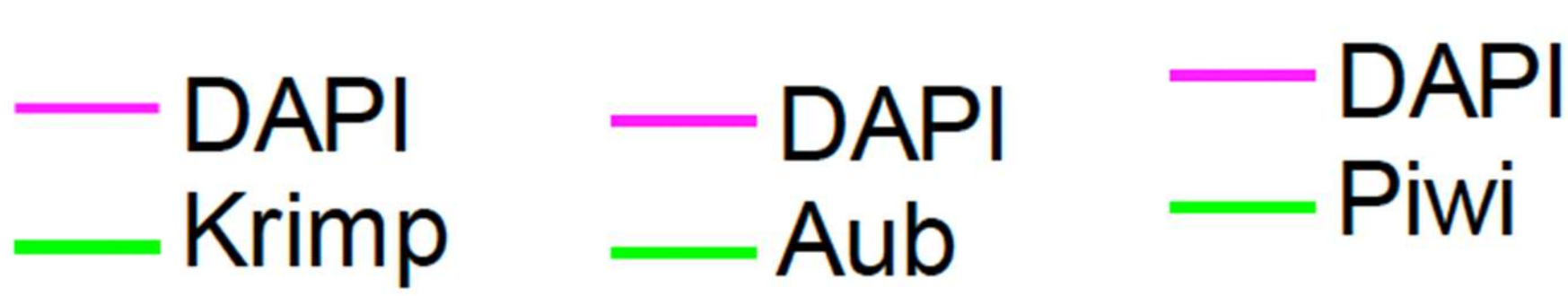

5. Figure 1 & 2
These figures could be combined as the data presented are strongly linked to provide a coherent and accessible flow.

Thank you for your suggestion. We agree with your opinion. First, we combined the related sections in the maintext. The two sections entitled "Labelling of PIWI proximity factors and purification from germline cells" and "Characterization of germline PIWI proximity proteomes" were combined to one section entitled "Biotin labelling and characterization of germline PIWI proximity factors."

We also considered combining Figure 1&2, but after adding single channel views in Figure 1 and especially, the volcano plots for Figure 2, it was found not mergeable. Keeping these panels in two figures would not seriously disturb the flow. Panels were re-sized (got smaller) having space for the legends, and each figure will not occupy a full page. Hope you accept our idea for this issue.

6. Figure 7E
Genome browser screenshots for piRNA levels still show scale factors in the background (black letters) this should be removed to avoid confusion
Thank you for finding this background. We removed the background letters and also checked other browser screenshots if there any background signals.

Last, we mention we added meta-profile for H3K4me3 and GFP-Set1 signals obtained by CUT&Tag in the revised manuscript. New Figure 5D for H3K4me3 CUT&Tag, and new Figure 7CE for GFP-Set1, as attached below. These results show the TSS-associated peaks formed by H3K4me3 and GFP-Set1. Set1 can show its enrichment on 3'UTRs of TEs with this heatmap, which is followed by track views on individual TE families.

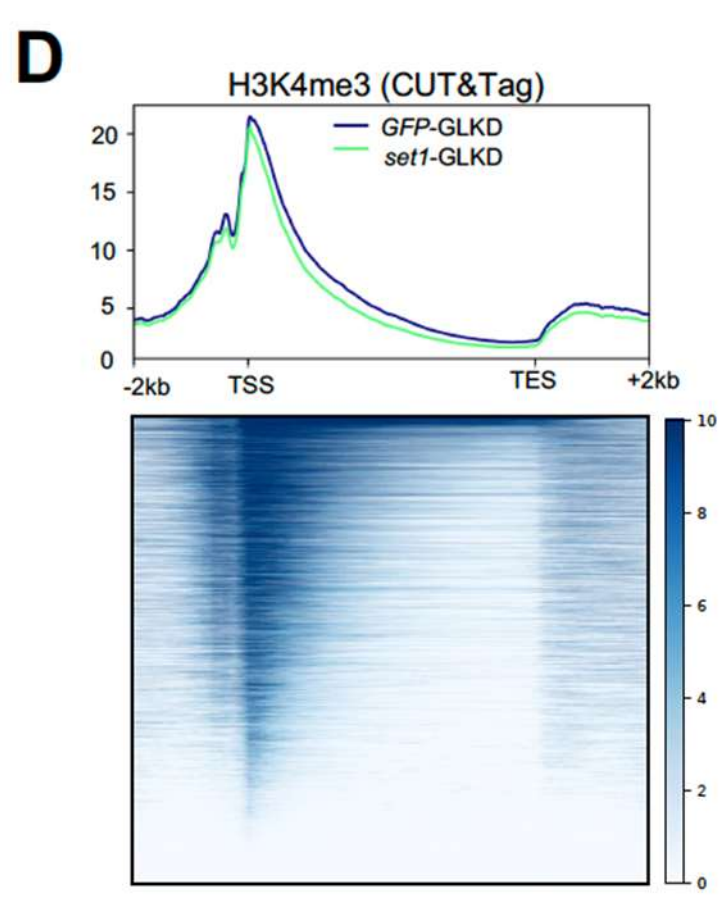

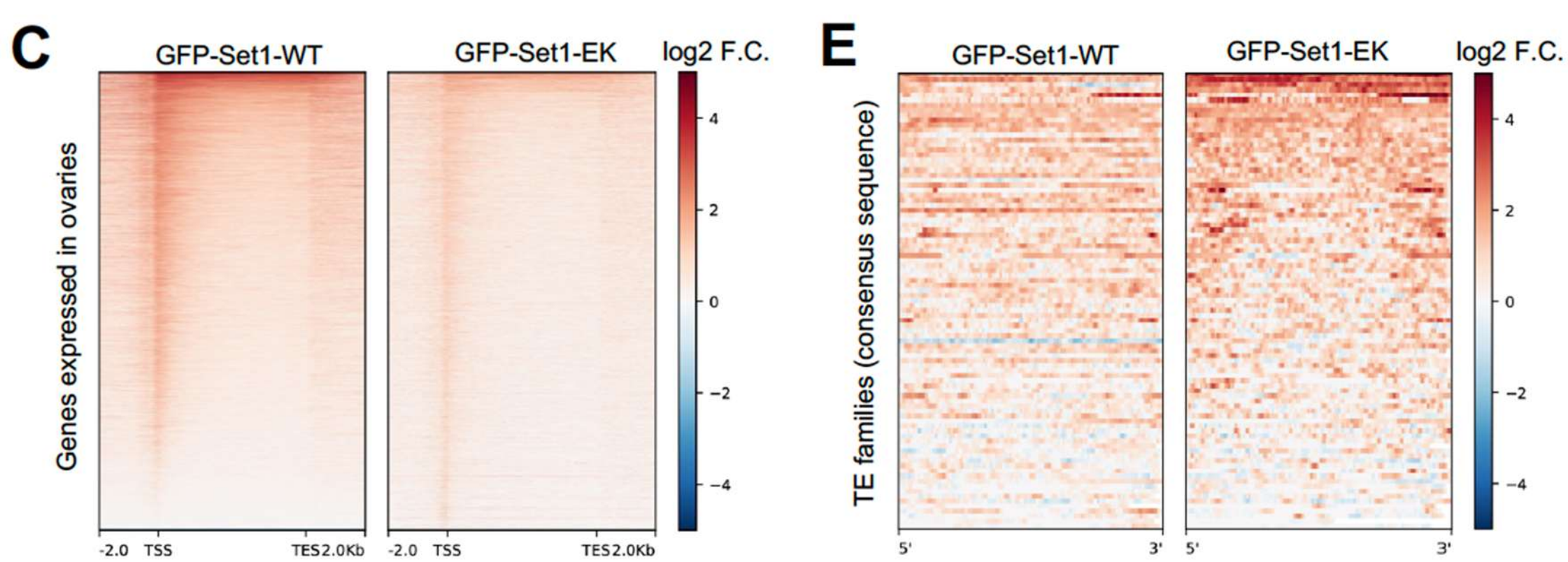

Referee #3:

I loved the paper. Through careful and comprehensive experiments, they have revealed a hitherto unknown function of SET1, which is a big advance.

The technical details are impeccable, but I feel the title undersells the paper, an alternative title such as "Set1-Mediated Antisense Transcription: A Methyltransferase-Independent Guard of the Drosophila Telomere" or ""Set1 Acts as a Catalytic-Independent Scaffold to Recruit the Piwi Pathway for Telomere Protection"" would help readers discern what is special in the paper and help drive more people to read it.

I believe any editing should be in the abstract and title to make it more impactful and help **"sell"** the paper to the audience. The way the paper is written is a little **underwhelming**. So I would shorten the paper a little bit and highlight the surprising parts, and let the piwi-proximity etc. (which led to SET1) be supporting actors. As you can see, I don't want to quibble about minor things in this paper, but want it to be more bold with its message.

Through painstaking experiments silencing 54 potential targets, they identified Set1 and Hrb27C as the most promising candidates for further study. While Hrb27C was linked to Aubergine, Set1 stood out because its depletion caused a significant accumulation of the HeT-A Gag protein, directly linking it to the silencing of telomeric transposons .

The paper is packed with several technical achievements too, such as the methodological optimization of mTurbo for the female germline. They identified that standard purification was inefficient in differentiating ovaries and overcame this by using GSCLCs (Germline Stem Cell-Like Cells) via bam knockdown to dramatically improve pulldown efficiency.
The use of the RNAi-resistant Set1 E1613K mutant and normal Set1 to demonstrate the catalytic-activity-independent function of Set1 is novel.

Great job, and a very convincing and thorough work, with all the lines of evidence (proteomic, genetic, transcriptomic, small RNA etc.) that has been amassed. I don't want to mention anything negative for the sake of it. I genuinely did not see anything amiss, but maybe some of the material could be moved to supplementary, for readers who might need the gory details. My advice could be counter to the journal policy, which I am not familiar with.

Thank you very much for your encouraging positive comments. Following the advises provided by other reviewers, we performed several new experiments, and prepared a revised manuscript containing new results. Critical improvement is in the mechanistic insight into Set1 in telomere silencing. By conducting CUT&Tag using anti-Rhino (Rhi) antibodies, we demonstrate that Set1 is crucial in recruiting Rhi to subtelomeric cluster loci. Hence, Rhi recruitment underlies Set1-mediated piRNA production and telomere silencing (new Figure 8 and Figure EV5, attached below). Moreover, germline depletion of Set1 led to overactivation of cluster loci including 38C. Hence, we proposed that Set1 is important not only for activating subtelomeric cluster loci, but also for controlling overall piRNA cluster activities in germline. Unfortunately, due to these progresses, the revised manuscript got longer than the original version, which is opposite to your advise. We understand selling the study is important, but would like to keep the title as it is. Thank you.

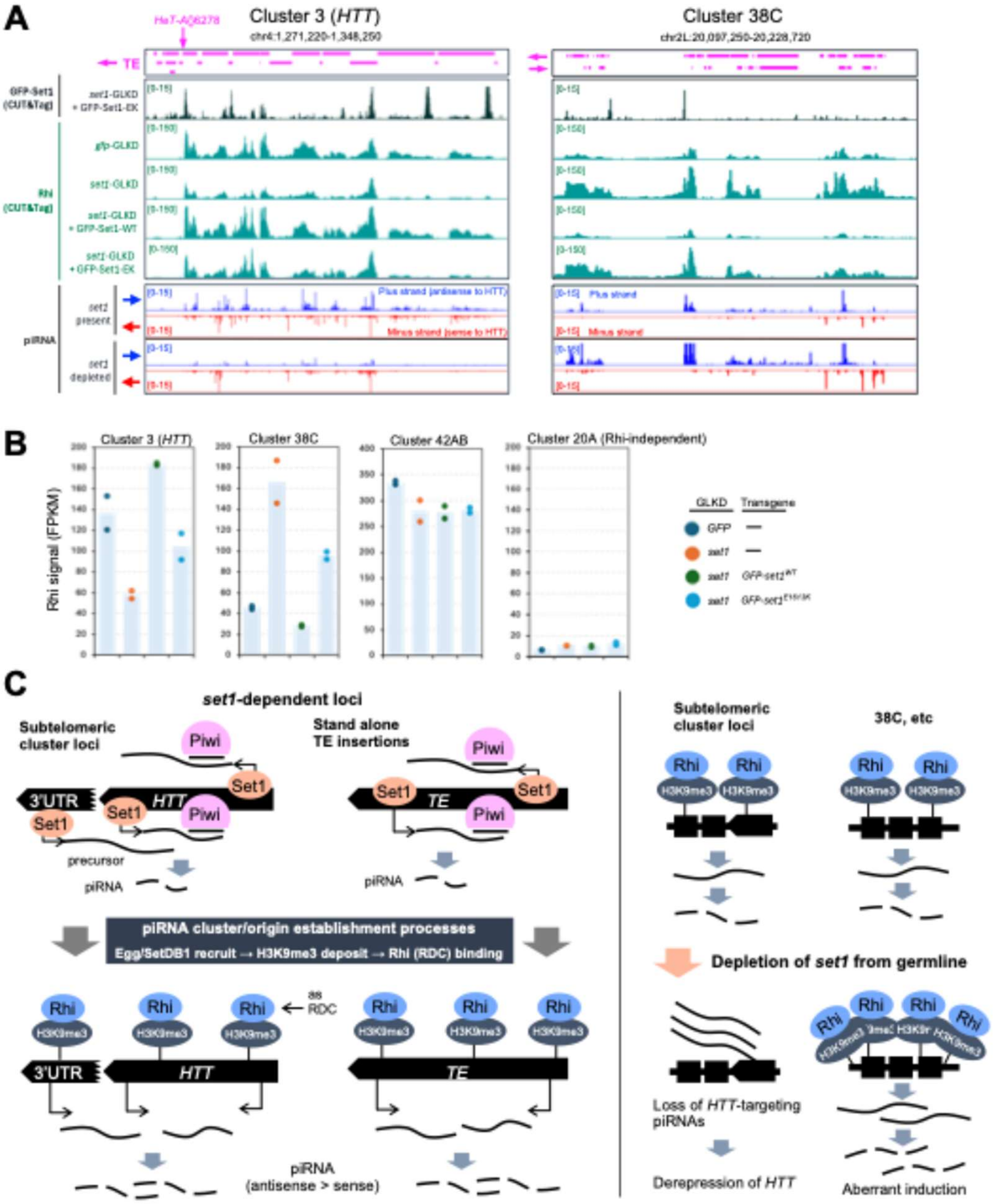

Figure 8. Effect of Set1 depletion on Rhi localization to piRNA cluster loci (A) Track view at subtelomeric cluster 3 or 38C locus. Light green: signals from anti-Rhi CUT&Tag. Dark green: GFP-Set1-EK signals in *set1*-GLKD (anti-GFP CUT&Tag). Blue and red: piRNAs mapped to genomic plus and minus strands, respectively. Mean CPM from biological replicates (n=2). (B) Quantification of anti-Rhi CUT&Tag signals. Bars: mean FPKM (fragments per kilobase per million mapped reads), dots: individual FPKM, from biological replicates (n=2). (C) Working model. Set1 interacting with *HTT* sequences cooperates with Piwi for piRNA cluster formation in subtelomeric regions. Set1 provides target templates for Piwi-piRNA complexes and/or precursor transcripts for piRNA production. Fragmented *HeT-A* 3'UTR retaining antisense TSS plays a crucial role in the latter process. Cooperation between Set1 and Piwi promotes H3K9me3 deposition and Rhi recruitment. Once this piRNA cluster status is established, Set1 and Piwi become dispensable. Similar mechanisms operate in the silencing of stand-alone TEs. Germline cells lacking the noncanonical functions of Set1 not only fail to establish piRNA clusters at subtelomeric loci, but also exhibit aberrant Rhi distribution and piRNA overproduction at certain loci.

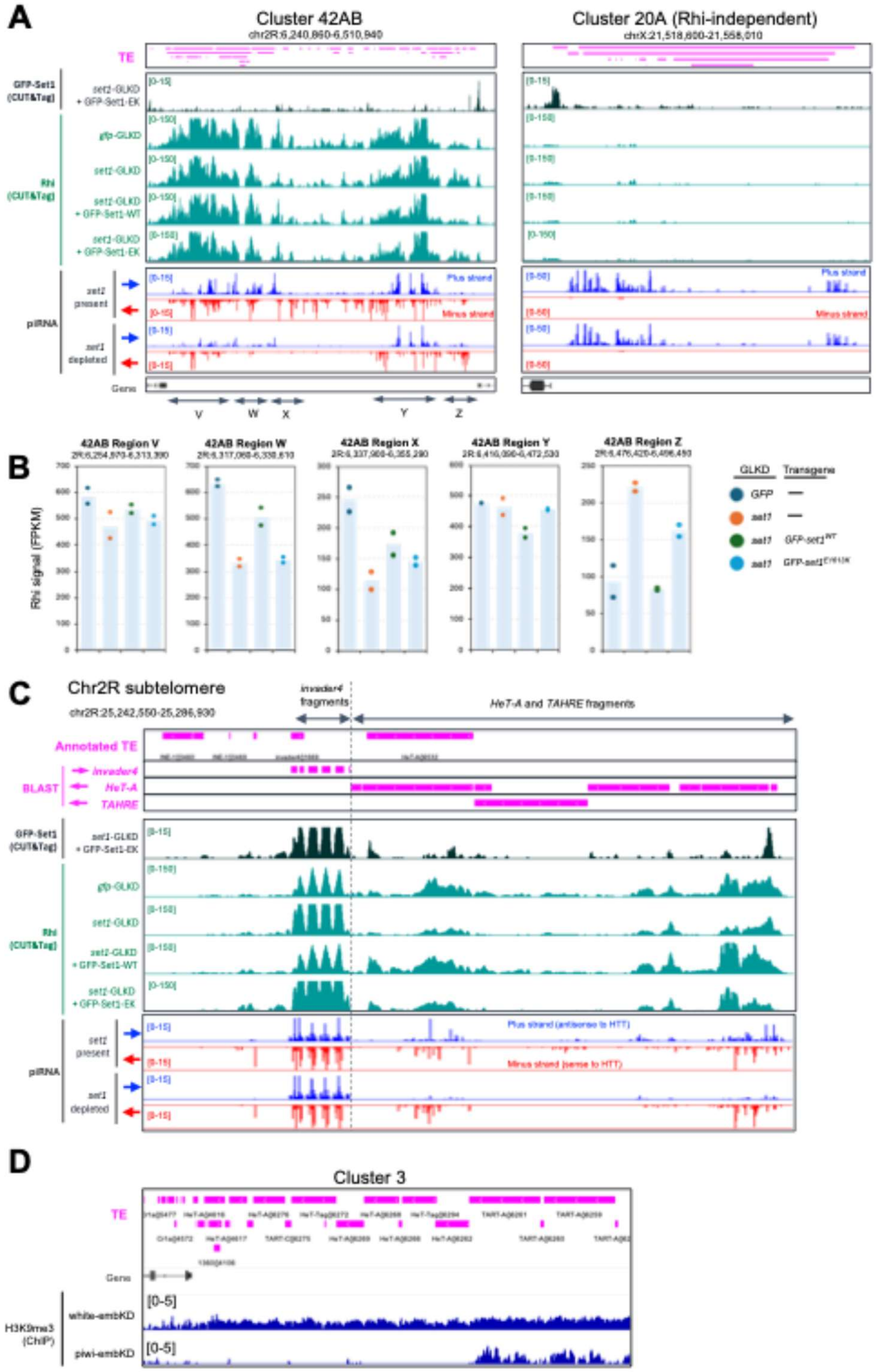

Figure EV5. Effect of Set1 depletion on Rhi localization at piRNA cluster loci, and effect of Piwi depletion on H3K9me3 deposition in subtelomeric cluster (A) Track view at 42AB or 20A locus. Light green: signals from anti-Rhi CUT&Tag. Dark green: GFP-Set1-EK signals (anti-GFP CUT&Tag). Blue and red: piRNAs mapped to genomic plus and minus strands, respectively. (B) Quantification of anti-Rhi CUT&Tag signals. Bars: mean FPKM, dots: individual FPKM, from biological replicates (n=2). Analyzed regions within 42AB are indicated in panel A. (C) Track view in the subtelomeric region on chromosome 2R. (D) Track view at subtelomeric cluster 3 showing H3K9me3 signal (mean CPM from biological replicates (n=2)). embKD: knockdown at embryonic stages. Raw data: GSE83238.

1st Revision - Editorial Decision **20th Jul 2026**

Dear Dr. Iki,

Thank you for the submission of your revised manuscript. We have now received the enclosed reports from the referees and I am happy to say that both support its publication now. Referee 2 still has a few more suggestions that I would like you to address and incorporate before we can proceed with the official acceptance of your manuscript.

A few editorial requests will also need to be addressed:

- Please correct the conflict of interest subheading to "Disclosure and Competing Interests Statement"

- The author credits need to be removed from the ms file. All credits need to be entered during online ms submission.

- It appears that one funder is missing in our online submission system - Grant from Open and Transdisciplinary Research Initiatives (OTRI) RNA Frontier Science Division. Please add this funder with your next ms submission.

- Figure 3 is missing label (C) in the figure file, please add.

- EV table legends need to be removed from the ms and from the additional tab/sheet in all tables; Table EV1 is a dataset and needs to be updated to Dataset EV1 in all places (source file name, legend, title in the system, ms callout); there is a mismatch between the file names and their titles in the system, this needs to be corrected.

- The APPENDIX FILE is missing page numbers in the ToC on the title page and throughout the file. However, you can also delete the Appendix file and instead have 8 EV figures, this is fine and probably makes it easier.

- The Source Data (SD) file type (dataset) and title of each file is incorrect: "Data Set 1B", "Data Set 3A", etc. SD need to be uploaded using Figure Source Data file type; also, all panels of one figure should be combined into zip folders, one folder per figure; the correct nomenclature should be Figure 1 Source Data (folder/panle names 1B, 1C, etc.)

- Materials and Methods should be Methods

- The manuscript sections should be in the following order: Title page - Abstract - Introduction - Results - Discussion - Methods - Data Availability - Acknowledgments - Disclosure Statement & Competing Interests - References - Figure Legends - (Main Tables with legends if applicable) - Expanded View Figure Legends.

* Figure Legends - Comments *

- Please note that information related to n is missing in the legends of figures 4A, B
- Please note that the measure of center for the error bars need to be defined in the legend of figure S3 D

I would like to suggest some minor changes to the title and abstract. Please let me know if you agree with this:

PIWI proximity proteome reveals Set1-mediated piRNA biogenesis for transposon silencing in telomeres

Silencing complexes formed by PIWI-clade Argonaute (Ago) proteins and PIWI-interacting RNAs (piRNAs) are essential guardians of genome integrity, controlling the deleterious activities of transposable elements (TEs) in the animal germline. However, our understanding of PIWI-piRNA-directed TE silencing remains incomplete. Here, we systemically characterize the proximity proteome of PIWI members, Piwi, Aubergine (Aub), and Ago3 in the germline of Drosophila ovaries. Functional screening identifies previously uncharacterized factors involved in TE silencing, including H3K4me3 writer and transcriptional coactivator Set1. Transcriptome analysis reveals that Set1 acts as an indispensable repressor of TEs, particularly of those forming telomeres. The involvement of Set1 in the Piwi pathway is further supported by its critical role in the production of antisense, TE-targeting piRNAs, requiring its catalytic activity. Genome-wide chromatin profiling by CUT&Tag demonstrates that Set1 preferentially associates with TE sequences including their 3'UTRs, and is localized at subtelomeric piRNA-producing loci, to which it recruits Rhino, a key activator of piRNA precursor transcription. Collectively, our findings uncover a noncanonical function of Set1 in Piwi-mediated TE silencing and telomere control in germline nuclei.

EMBO press papers are accompanied online by A) a short (1-2 sentences) summary of the findings and their significance, B) 2-3 bullet points highlighting key results and C) a synopsis image that is exactly 550 pixels wide and 300 pixels high. The synopsis image should provide a sketch of the major findings, like a graphical abstract. Please note that text needs to be readable at the final size. Please send us this information along with the final manuscript.

I look forward to seeing a final version of your manuscript as soon as possible. Please use this link to submit your revision: https://embor.msubmit.net/cgi-bin/main.plex

Best regards,
Esther

Esther Schnapp, PhD
Senior Editor
EMBO reports

Referee #1:

I would like to thank the authors for their careful revision. All of the points I raised in my earlier review have been appropriately addressed, and the manuscript now reads clearly and convincingly. In my assessment, the paper is suitable for publication in its present form, and I recommend acceptance.

Referee #2:

Summary:

Isshiki and colleagues improved their revised manuscript by exploring the connection between Set1 and Rhino. This points towards an interesting observation that Rhino binding to subtelomeric piRNA-producing loci is affected by the presence of Set1, however, independent of its catalytic activity. The authors also implemented many of the suggested changes made by the reviewers, which improve the accessibility of the data. However, as you see from my comments below, there are still remaining issues in terms of data presentation, interpretation and writing that should be addressed before publication.

Abstract:
Line 31: There, Set1 recruits Rhino, a key activator of piRNA precursor transcription.
The paper does not show a recruitment of Rhino by Set1, but shows that its proper localisation depends on the presence of Set1. Without more direct evidence, the authors should refrain from inferring direct recruitment at any point in the manuscript.

For Figure 5B show single chanel images, in the current presentation it is very hard to interpret the data.

Figure 5D - This presentation of the data is not helpful for the reader to understand. I assume that the majority of the signal is derived from the somatic follicle cells, which are not depleted for Set1, and therefore no reduction is seen. The authors should make a comprehensive list of germline-expressed genes (e.g., based on single-cell RNA-seq: Rust, K., Byrnes, L.E., Yu, K.S. et al. A single-cell atlas and lineage analysis of the adult Drosophila ovary. Nat Commun 11, 5628 (2020). https://doi.org/10.1038/s41467-020-19361-0) to show the effect of their GLKD and then also the effect of their rescues.

Figure EV3A has no MW markers. Also, why is there no second image of the membrane, like in Figure 6A, to show the expression of GFP? In this blot it appears to be at the same size as GFP-Set1, which, according to Figure 6A, runs at ~250 kDa.

Figure 6E: All the mutants should be shown using the same scale, as the authors make comparisons of their effects.

Line 297: Instead, H3K4me3 signals on TEs were positively correlated with the transcript abundance (Figure 5C), suggesting that these marks are deposited through transcription of TEs either by residual Set1 in set1-depleted germline cells or through Set1-independent mechanisms (Ardehali et al, 2011).

Figure 5C shows only TPM levels for different TEs; no correlation with H3K4me3 is shown, so this section is not supported.

Line 385: Moreover, Set1 can silence TEs, 386 HTT members in particular, without its catalytic activity (Figure 5).
In the paper it is not shown that Set1 can silence TEs. The authors should revise the manuscript in a way that they do not infer strong mechanistic conclusions when there are no directly supporting experimnts for the conclusions.

Line 403: This absence suggests that our proteome dataset is biased toward a specific stage which might precede H3K9me3 deposition.
In the absence of any experimental evidence for this model, the authors should at least mention that the absence of SetDB1 in their data could simply be technical, or could reflect that Piwi does not recruit SetDB1 by a mechanism that is detectable via the biotinylation radius of the fused protein.

Language:

The sentences below are grammatically imprecise or awkwardly phrased and affect readability; I flag them here so the authors can revise the wording for clarity.

Line 160: However, HeT-A Gag protein was under detectable level (Figure 3A).

Line 162: In both hrb27c- and set1-GLKD conditions, perinuclear Krimp signals could be weaker but still observed (Figure 3B).

Line 262: Supporting a link to Piwi pathway, public data re-mining revealed a similar antisense-biased reduction of HTT-mapping piRNAs in panx-deficient ovaries (Figure 6E)

Line 329: Depletion of set1 from germline (set1-GLKD) caused a striking reduction of Rhi signals at cluster 3 locus (Figure 8AB).

Figure 4: Please mark somehow which TEs are significantly regulated. The label "Tes/ Genes included in EdgeER output" is not clear an can be improved for clarity

We thank the editor and the reviewers for all of their supports to improve our manuscript for publication. Below we provide our point-by-point response for the 2nd round of review.

A few editorial requests will also need to be addressed:
- Please correct the **conflict of interest** subheading to "Disclosure and Competing Interests Statement"
Corrected.
- The author credits need to be removed from the ms file. All credits need to be entered during online ms submission.
Removed from ms.
- It appears that one funder is missing in our online submission system - Grant from Open and Transdisciplinary Research Initiatives (OTRI) RNA Frontier Science Division. Please add this funder with your next ms submission.
Will be added upon online submission.
- Figure 3 is missing label (C) in the figure file, please add.
Added.
- EV table legends need to be removed from the ms and from the additional tab/sheet in all tables; **Table EV1 is a dataset** and needs to be updated to **Dataset EV1** in all places (source file name, legend, title in the system, ms callout); there is a mismatch between the file names and their titles in the system, this needs to be corrected.
Corrected. (As a response to Reviewer#2, we generated new Dataset EV2).
- The APPENDIX FILE is missing page numbers in the ToC on the title page and throughout the file. However, you can also delete the Appendix file and instead have **8 EV figures**, this is fine and probably makes it easier.
We deleted Appendix file and made 8 EV figures instead.
- The Source Data (SD) file type (dataset) and title of each file is incorrect: "Data Set 1B", "Data Set 3A", etc. SD need to be uploaded using **Figure Source Data file type**; also, **all panels of one figure should be combined into zip folders**, **one folder per figure**; the correct nomenclature should be **Figure 1 Source Data** (folder/panle names 1B, 1C, etc.)
Corrected.
- Materials and Methods should be Methods
Corrected.
- The manuscript sections should be in the following order: Title page - Abstract - Introduction - Results - Discussion - Methods - Data Availability - Acknowledgments - Disclosure Statement & Competing Interests - References - Figure Legends - (Main Tables with legends if applicable) - Expanded View Figure Legends.
Corrected.

* Figure Legends - Comments *
- Please note that information related to n is missing in the legends of figures 4A, B
"Biological replicates (n=2)." was added in the legend.
- Please note that the measure of center for the error bars need to be defined in the legend of figure S3 D
"The center of error bars: average values." was added in the legend.

I would like to suggest some minor changes to the title and abstract.
Please let me know if you agree with this:

PIWI proximity proteome reveals Set1-mediated piRNA biogenesis for transposon silencing in telomeres

Silencing complexes formed by PIWI-clade Argonaute (Ago) proteins and PIWI-interacting RNAs (piRNAs) are essential guardians of genome integrity, controlling the deleterious activities of transposable elements (TEs) in the animal germline. However, our understanding of PIWI-piRNA-directed TE silencing remains incomplete. Here, we systemically characterize the proximity proteome of PIWI members, Piwi, Aubergine (Aub), and Ago3 in the germline of Drosophila ovaries. Functional screening identifies previously uncharacterized factors involved in TE silencing, including H3K4me3 writer and transcriptional coactivator Set1. Transcriptome analysis reveals that Set1 acts as an indispensable repressor of TEs, particularly of those forming telomeres. The involvement of Set1 in the Piwi pathway is further supported by its critical role in the production of antisense, TE-targeting piRNAs, requiring its catalytic activity. Genome-wide chromatin profiling by CUT&Tag demonstrates that Set1 preferentially associates with TE sequences including their 3'UTRs, and is localized at subtelomeric piRNA-producing loci, to which it recruits Rhino, a key activator of piRNA precursor transcription. Collectively, our findings uncover a noncanonical function of Set1 in Piwi-mediated TE silencing and telomere control in germline nuclei.
Thank you for your suggestions. We follow your ideas, but one thing, "requiring its catalytic activity" is not correctly explaining our results. Our EK mutant (catalytic mutant) rescued the phenotypes to some extent (though not perfect like WT). Therefore, we would like to keep "Notably, catalytic activity of Set1 is dispensable."

We re-considered where to place this sentence. We moved this short sentence after dscribing Rhino phenotypes, because EK is functional for Rhi accumulation. And, it gives a good connection to "noncanonical" in the last sentence.

Because of word limitation, "the" before Piwi pathway is omitted.

Since reviewer#2 asked us to avoid using "recruit" for Rhino, "recruits" has been replaced with "accumulates." Below will be the final form of Abstract.

Silencing complexes formed by PIWI-clade Argonaute (Ago) proteins and PIWI-interacting RNAs (piRNAs) are essential guardians of genome integrity, controlling the deleterious activities of transposable elements (TEs) in animal germline. However, our understanding of PIWI-piRNA-directed TE silencing remains incomplete. Here, we systemically characterize the proximity proteome of PIWI members, Piwi, Aubergine (Aub), and Ago3 in the germline of *Drosophila* ovaries. Functional screening identifies previously uncharacterized factors involved in TE silencing, including H3K4me3 writer and transcriptional coactivator Set1. Transcriptome analysis reveals that Set1 acts as an indispensable repressor of TEs, particularly of those forming telomeres. The involvement of Set1 in Piwi pathway is further supported by its critical role in the production of antisense, TE-targeting piRNAs. Genome-wide chromatin profiling by CUT&Tag demonstrates that Set1 preferentially associates with TE sequences including their 3'UTRs, and is localized at subtelomeric piRNA-producing loci, to which it accumulates Rhino, a key activator of piRNA precursor transcription. Notably, catalytic activity of Set1 is dispensable. Collectively, our findings uncover a noncanonical function of Set1 in Piwi-mediated TE silencing and telomere control in germline nuclei.
(175 words)

EMBO press papers are accompanied online by
A) a short (1-2 sentences) summary of the findings and their significance,
PIWI proteins and piRNAs protect the germline genome from transposable elements, yet the mechanisms underlying this defense remain incompletely understood. By characterizing the proximity proteome of PIWI members in the female germline of *Drosophila melanogaster*, we show that histone methyltransferase Set1 plays a noncanonical role in piRNA biogenesis and transposon silencing in telomeres.

B) 2-3 bullet points highlighting key results and
1. Proximity proteomes of the three Drosophila PIWI proteins (Piwi, Aub, and Ago3) identify previously uncharacterized factors involved in germline transposon silencing.
2. Histone methyltransferase Set1 plays a noncanonical role in the Piwi pathway to promote piRNA biogenesis and silence telomeric transposons.
3. Transposable elements 3'UTRs can serve as cis-elements used for Set1-dependent piRNA biogenesis and genome defense.

C) a synopsis image that is exactly 550 pixels wideand 300 pixels high.
The synopsis image should provide a **sketch of the major findings**, like a graphical abstract.
Please note that text needs to be readable at the final size. Please send us this information along with the final manuscript.
Prepared.

I look forward to seeing a final version of your manuscript as soon as possible. Please use this link to submit your revision: https://embor.msubmit.net/cgi-bin/main.plex
Best regards,
Esther
Esther Schnapp, PhD
Senior Editor
EMBO reports

Referee #1:
I would like to thank the authors for their careful revision. All of the points I raised in my earlier review have been appropriately addressed, and the manuscript now reads clearly and convincingly. In my assessment, the paper is suitable for publication in its present form, and I recommend acceptance.
Thank you very much for your positive response to our revised manuscript.

Referee #2:
Summary:
Isshiki and colleagues improved their revised manuscript by exploring the connection between Set1 and Rhino.
This points towards an interesting observation that Rhino binding to subtelomeric piRNA-producing loci is affected by the presence of Set1, however, independent of its catalytic activity. The authors also implemented many of the suggested changes made by the reviewers, which improve the accessibility of the data. However, as you see from my comments below, there are still remaining issues in terms of data presentation, interpretation and writing that should be addressed before publication.
Thank you very much for your positive response and suggestions for further improvement.

Abstract:

Line 31: There, Set1 **recruits** Rhino, a key activator of piRNA precursor transcription.

The paper does not show a recruitment of Rhino by Set1, but shows that its proper localisation depends on the presence of Set1. Without more direct evidence, the authors should refrain from inferring direct recruitment at any point in the manuscript.

Thank you for your advise. We replaced "recruit" with "accumulate" in the final version, including line 31 and other sentences dscribing Rhino phenotypes.

For Figure 5B show single chanel images, in the current presentation it is very hard to interpret the data.

In the final version of Figure 5B, the single chanel images were included, as attached below.

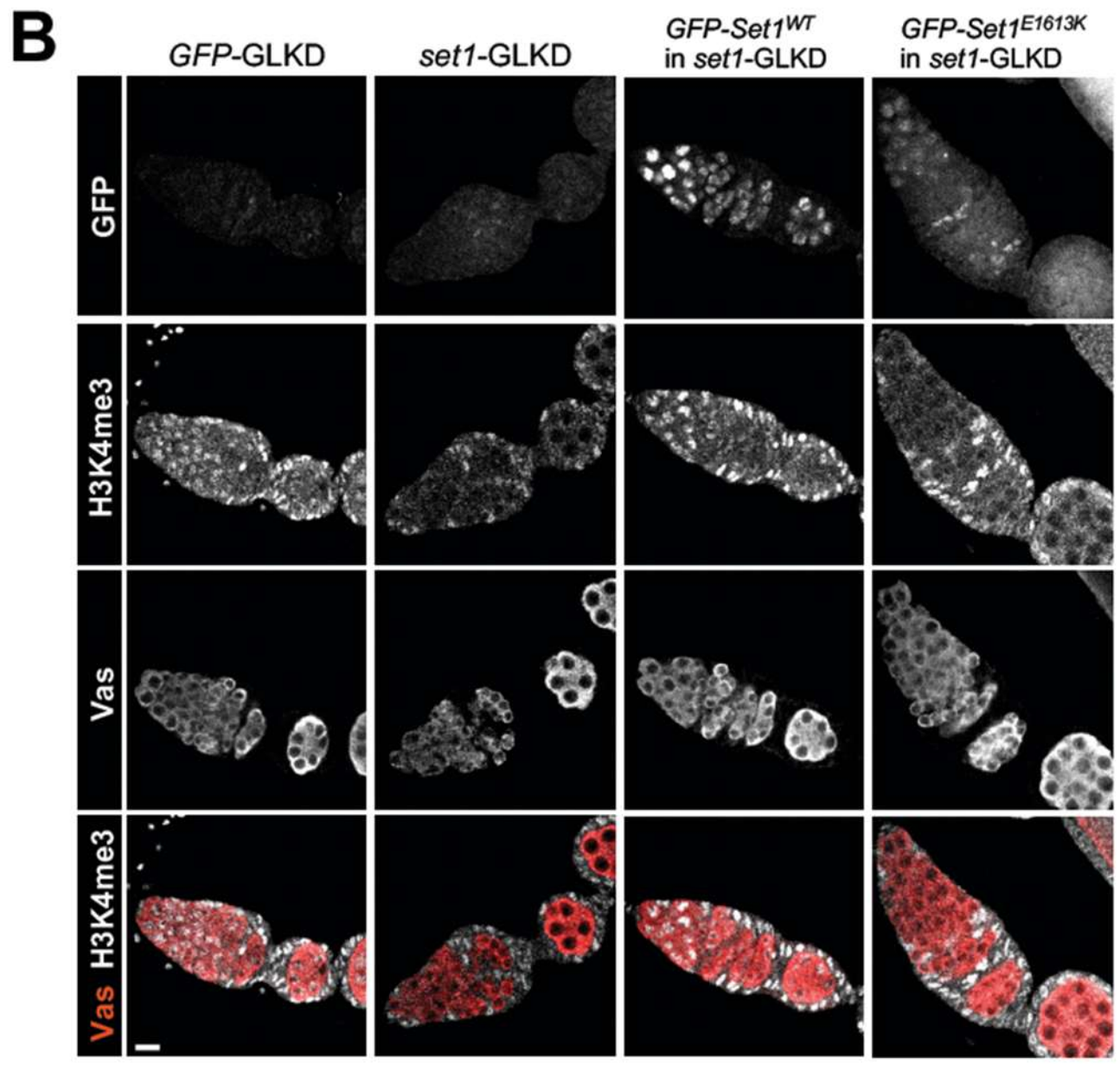

Figure 5D - This presentation of the data is not helpful for the reader to understand. I assume that the majority of the signal is derived from the somatic follicle cells, which are not depleted for Set1, and therefore no reduction is seen. The authors should make a comprehensive list of germline-expressed genes (e.g., based on single-cell RNAseq: Rust, K., Byrnes, L.E., Yu, K.S. et al. A single-cell atlas and lineage analysis of the adult Drosophila ovary. NatCommun 11, 5628 (2020). https://doi.org/10.1038/s41467-020-19361-0) to show the effect of their GLKD and then also the effect of their rescues.

Thank you very much for your advise. I guess the reviewer expects us to provide the profile using a set of genes exclusively expressed in germline but not in somatic cells of ovaries. I checked the data from the reference, but could not find useful gene list. I found the list of germline genes in Supplementary Data 8, but this list (n=1930) includes genes (e.g., *piwi*) expressed in both germline and soma lineages.

The data I checked is attached below.

Title: Supplementary Data 8: Description: Candidate genes changing expression in germ cell pseudotime identified by monocle3. This table was created using the monocle3 v0.2.1 graph_test command

Below is the metaprofile for germline genes listed from Rust et al. 2020. As expected, the pattern is similar to that we got using our gene set. This would be because both lists contain genes expressed in gonadal soma.

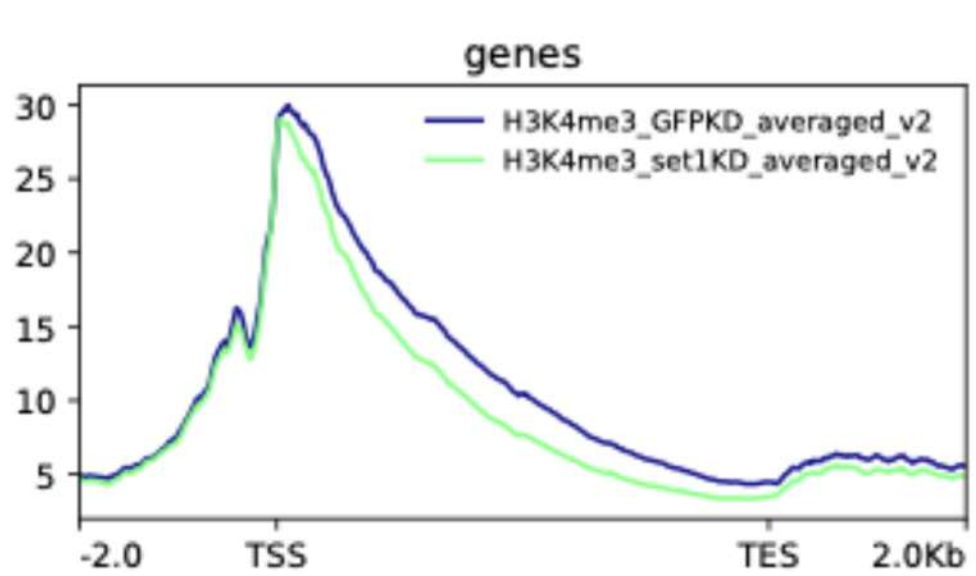

We think the current Figure 5D is helpful for readers to know that H3K4me3 libraries contains many signals which are not affected by set1-GLKD. This is, as you mentioned, because of the signals from genes expressed in somatic cells including follicle cells. To measure the effect of set1-GLKD and transgenes on germline-restricted genes, the presence of somatic signals is useful for normalization process. Therefore, we would like to keep the Figure 5D in the revised manuscript as it is.

To explain the meaning of presenting this metaprofile, we amended the sentence related to Figure 5D, as below.
"The patterns and signals were comparable between *gfp*- and *set1*-GLKD conditions, when all ovary-expressed genes were considered, regardless of their germline specificity."

Figure EV3A has no MW markers. Also, why is there no second image of the membrane, like in Figure 6A, to show the expression of GFP? In this blot it appears to be at the same size as GFP-Set1, which, according to Figure 6A, runs at ~250 kDa.
Thank you for pointing this out. MW markers have been added in the Figure EV3A.
We realized that the negative control condition in the non-crosslinking experiment is sibling control, not a condition expressing GFP. We sincerely apologize this mistake. The indication in Figure EV6A was corrected.

Figure 6E: All the mutants should be shown using the same scale, as the authors make comparisons of their effects.
Thank you for your advise. Same scale has been applied for all mutants in Figure 6E.

Line 297: Instead, H3K4me3 signals on TEs were positively correlated with the transcript abundance (Figure 5C), suggesting that these marks are deposited through transcription of TEs either by residual Set1 in set1-depleted germline cells or through Set1-independent mechanisms (Ardehali et al, 2011). Figure 5C shows only TPM levels for different TEs; no correlation with H3K4me3 is shown, so this section is not supported.
Thank you for your comment. Indeed, Figure 5C does not show the correlation. We have replaced "positively correlated" with "associated with" in line 297.

Line 385: Moreover, Set1 can silence TEs, 386 HTT members in particular, without its catalytic activity (Figure 5).
In the paper it is not shown that Set1 can **silence** TEs. The authors should revise the manuscript in a way that they do not infer strong mechanistic conclusions when there are no directly supporting experimnts for the conclusions.
Thank you for your comment. We have replaced "silence" with "repress" in line 385.

Line 403: This absence suggests that our proteome dataset is biased toward a specific stage which might precede H3K9me3 deposition.
In the absence of any experimental evidence for this model, the authors should at least mention that the absence of SetDB1 in their data could simply be technical, or could reflect that Piwi does not recruit SetDB1 by a mechanism that is detectable via the biotinylation radius of the fused protein.
Thank you for your thorough reading and valuable comment. We rephrased the sentence, as attached below.
"This absence could be due to the technical limitations of proximity labeling, or it may suggest that our proteome dataset is biased toward a specific stage preceding H3K9me3 deposition."

Language:
The sentences below are grammatically imprecise or awkwardly phrased and affect readability; I flag them here so the authors can revise the wording for clarity.
Line 160: However, HeT-A Gag protein was under detectable level (Figure 3A).
Corrected as "However, *HeT-A* Gag protein was below the detectable level (Figure 3A)."

Line 162: In both hrb27c- and set1-GLKD conditions, perinuclear Krimp signals could be weaker but still observed (Figure 3B).
Corrected as "In both hrb27c- and set1-GLKD conditions, perinuclear Krimp signals were weaker but still detectable (Figure 3B)."

Line 262: Supporting a link to Piwi pathway, public data re-mining revealed a similar antisense-biased reduction of HTT-mapping piRNAs in panx-deficient ovaries (Figure 6E)
Corrected as "Supporting a link to the Piwi pathway, re-mining of public datasets revealed a similar antisense-biased reduction in *HTT*-mapping piRNAs in *panx*-deficient ovaries (Figure 6E)."

Line 329: Depletion of set1 from germline (set1-GLKD) caused a striking reduction of Rhi signals at cluster 3 locus (Figure 8AB).
Corrected as "Depletion of Set1 from the germline (set1-GLKD) caused a striking reduction in Rhi signals at the cluster 3 locus (Figure 8AB)."

Figure 4: Please mark somehow which TEs are significantly regulated. The label "Tes/ Genes included in EdgeER output" is not clear an can be improved for clarity.
Thank you for your advise. We prepared new **Dataset EV2** that contains information of all TE insertions analyzed by EdgeR in Figure 4AB.

Line 184 "For Set1 identified as the proximity factor of Piwi (Figure 2), its GLKD caused remarkable derepression of a subset of TEs in non-GSCLC ovaries (Figure 4A, EV4A, **Dataset EV2**)."

Dr. Taichiro Iki
Kyoto University
Graduate School of Biostudies
Konoe-machi, Yoshida
Sakyo-ku
Kyoto, Kyoto 606-8501
Japan

Dear Dr. Iki,

I am very pleased to accept your manuscript for publication in the next available issue of EMBO reports. Thank you for your contribution to our journal.

Your manuscript will be processed for publication by EMBO Press. It will be copy edited and you will receive page proofs prior to publication. Please note that you will be contacted by Springer Nature Author Services to complete licensing and payment information.

You may qualify for financial assistance for your publication charges - either via a Springer Nature fully open access agreement or an EMBO initiative. Check your eligibility: https://link.springer.com/journal/44319/how-to-publish-with-us

Should you be planning a Press Release on your article, please notify the production team via this form in order to coordinate publication and release dates: https://forms.cloud.microsoft/e/eH4A8JVP6g

If you have any questions, please do not hesitate to contact the Editorial Office. Thank you for your contribution to EMBO Reports.

Best regards,
Esther

Esther Schnapp, PhD
Senior Editor
EMBO reports

>>> Please note that it is EMBO Reports policy for the transcript of the editorial process (containing referee reports and your response letter) to be published as an online supplement to each paper. If you do NOT want this, you will need to inform the Editorial Office via email immediately. More information is available here: https://link.springer.com/partners/embo-press/editorial-policies#Peer%20review

EMBO Press Author Checklist

Corresponding Author Name: Taichiro Iki	
Journal Submitted to: EMBO Reports	
Manuscript Number: EMBOR-2026-64135-T	

USEFUL LINKS FOR COMPLETING THIS FORM
The EMBO Journal - Author Guidelines
EMBO Reports - Author Guidelines
Molecular Systems Biology - Author Guidelines
EMBO Molecular Medicine - Author Guidelines

Reporting Checklist for Life Science Articles (updated January
This checklist is adapted from Materials Design Analysis Reporting (MDAR) Checklist for Authors. MDAR establishes a minimum set of requirements in transparent reporting in the life sciences (see Statement of Task: 10.31222/osf.io/9sm4x). Please follow the journal's guidelines in preparing your
Please note that a copy of this checklist will be published alongside your article.

Abridged guidelines for figures
1. Data
The data shown in figures should satisfy the following conditions:
- ➞ the data were obtained and processed according to the field's best practice and are presented to reflect the results of the experiments in an accurate and unbiased manner.
- ➞ ideally, figure panels should include only measurements that are directly comparable to each other and obtained with the same assay.
- ➞ plots include clearly labeled error bars for independent experiments and sample sizes. Unless justified, error bars should not be shown for technical
- ➞ if n<5, the individual data points from each experiment should be plotted. Any statistical test employed should be justified.
- ➞ Source Data should be included to report the data underlying figures according to the guidelines set out in the authorship guidelines on Data

2. Captions
Each figure caption should contain the following information, for each panel where they are relevant:
- ➞ a specification of the experimental system investigated (eg cell line, species name).
- ➞ the assay(s) and method(s) used to carry out the reported observations and measurements.
- ➞ an explicit mention of the biological and chemical entity(ies) that are being measured.
- ➞ an explicit mention of the biological and chemical entity(ies) that are altered/varied/perturbed in a controlled manner.
- ➞ the exact sample size (n) for each experimental group/condition, given as a number, not a range;
- ➞ a description of the sample collection allowing the reader to understand whether the samples represent technical or biological replicates (including how many animals, litters, cultures, etc.).
- ➞ a statement of how many times the experiment shown was independently replicated in the laboratory.
- ➞ definitions of statistical methods and measures:
  - common tests, such as t-test (please specify whether paired vs. unpaired), simple χ2 tests, Wilcoxon and Mann-Whitney tests, can be unambiguously identified by name only, but more complex techniques should be described in the methods section;
  - are tests one-sided or two-sided?
  - are there adjustments for multiple comparisons?
  - exact statistical test results, e.g., P values = x but not P values < x;
  - definition of 'center values' as median or average;
  - definition of error bars as s.d. or s.e.m.

Please complete ALL of the questions below.
Select "Not Applicable" only when the requested information is not relevant for your study.

Materials

Newly Created Materials	Information included in the manuscript?	In which section is the information available? (Reagents and Tools Table, Materials and Methods, Figures, Data Availability Section)
New materials and reagents need to be available; do any restrictions apply?	Not Applicable	No restrictions. We provide fly lines we generated upon request.

Antibodies	Information included in the manuscript?	In which section is the information available? (Reagents and Tools Table, Materials and Methods, Figures, Data Availability Section)
For **antibodies** provide the following information:<br>- Commercial antibodies: RRID (if possible) or supplier name, catalogue number and or/clone number<br>- Non-commercial: RRID or citation	Yes	Detail info in Reagents and Tools Table. Information can be found in Materials and Methods section.

DNA and RNA sequences	Information included in the manuscript?	In which section is the information available? (Reagents and Tools Table, Materials and Methods, Figures, Data Availability Section)
**Short novel DNA or RNA including primers, probes**: provide the sequences.	Yes	Reagents and Tools Table. We also provide info in Table EV5.

Cell materials	Information included in the manuscript?	In which section is the information available? (Reagents and Tools Table, Materials and Methods, Figures, Data Availability Section)
**Cell lines:** Provide species information, strain. Provide accession number in repository **OR** supplier name, catalog number, clone number, and/**OR** RRID.	Not Applicable	
**Primary cultures:** Provide species, strain, sex of origin, genetic modification status.	Not Applicable	
Report if the cell lines were recently **authenticated** (e.g., by STR profiling) and tested for mycoplasma contamination.	Not Applicable	

Experimental animals	Information included in the manuscript?	In which section is the information available? (Reagents and Tools Table, Materials and Methods, Figures, Data Availability Section)
**Laboratory animals or Model organisms:** Provide species, strain, sex, age, genetic modification status. Provide accession number in repository **OR** supplier name, catalog number, clone number, **OR** RRID.	Yes	Reagents and Tools Table. We also provide info in Table EV4.
**Animal observed in or captured from the field:** Provide species, sex, and age where possible.	Not Applicable	
Please detail **housing and husbandry conditions**.	Yes	Methods section in the maintext

Plants and microbes	Information included in the manuscript?	In which section is the information available? (Reagents and Tools Table, Materials and Methods, Figures, Data Availability Section)
**Plants:** provide species and strain, ecotype and cultivar where relevant, unique accession number if available, and source (including location for collected wild specimens).	Not Applicable	
**Microbes:** provide species and strain, unique accession number if available, and source.	Not Applicable	

Human research participants	Information included in the manuscript?	In which section is the information available? (Reagents and Tools Table, Materials and Methods, Figures, Data Availability Section)
If collected and within the bounds of privacy constraints report on age, sex and gender or ethnicity for all study participants.	Not Applicable	

Core facilities	Information included in the manuscript?	In which section is the information available? (Reagents and Tools Table, Materials and Methods, Figures, Data Availability Section)
If your work benefited from core facilities, was their service mentioned in the acknowledgments section?	Yes	Methods section in the maintext

Design

Study protocol	Information included in the manuscript?	In which section is the information available? (Reagents and Tools Table, Materials and Methods, Figures, Data Availability Section)
If study protocol has been **pre-registered, provide DOI in the manuscript**. For clinical trials, provide the trial registration number **OR** cite DOI.	Not Applicable	
Report the **clinical trial registration number** (at ClinicalTrials.gov or equivalent), where applicable.	Not Applicable	

Laboratory protocol	Information included in the manuscript?	In which section is the information available? (Reagents and Tools Table, Materials and Methods, Figures, Data Availability Section)
Provide DOI OR other citation details if **external detailed step-by-step protocols** are available.	Not Applicable	

Experimental study design and statistics	Information included in the manuscript?	In which section is the information available? (Reagents and Tools Table, Materials and Methods, Figures, Data Availability Section)
Include a statement about **sample size** estimate even if no statistical methods were used.	Yes	in Figure legend and Methods section in maintext
Were any steps taken to minimize the effects of subjective bias when allocating animals/samples to treatment (e.g. **randomization procedure**)? If yes, have they been described?	Not Applicable	
Include a statement about **blinding** even if no blinding was done.	Not Applicable	
Describe **inclusion/exclusion criteria** if samples or animals were excluded from the analysis. Were the criteria pre-established?<br><br>If sample or data points were omitted from analysis, report if this was due to attrition or intentional exclusion and provide justification.	Not Applicable	
For every figure, are **statistical tests** justified as appropriate? Do the data meet the assumptions of the tests (e.g., normal distribution)? Describe any methods used to assess it. Is there an estimate of variation within each group of data? **I**s the variance similar between the groups that are being statistically compared?	Yes	in Figure legend

Sample definition and in-laboratory replication	Information included in the manuscript?	In which section is the information available? (Reagents and Tools Table, Materials and Methods, Figures, Data Availability Section)
In the figure legends: state number of times the experiment was **replicated** in laboratory.	Yes	in Figure legend
In the figure legends: define whether data describe **technical or biological replicates**.	Yes	in Figure legend

Ethics

Ethics	Information included in the manuscript?	In which section is the information available? (Reagents and Tools Table, Materials and Methods, Figures, Data Availability Section)
Studies involving **human participants**: State details of **authority granting ethics approval** (IRB or equivalent committee(s), provide reference number for approval.	Not Applicable	
Studies involving **human participants**: Include a statement confirming that **informed consent** was obtained from all subjects and that the experiments conformed to the principles set out in the WMA Declaration of Helsinki and the Department of Health and Human Services Belmont Report.	Not Applicable	
Studies involving **human participants:** For publication of **patient photos**, include a statement confirming that consent to publish was obtained.	Not Applicable	
Studies involving experimental **animals**: State details of **authority granting ethics approval** (IRB or equivalent committee(s), provide reference number for approval. Include a statement of compliance with ethical regulations.	Not Applicable	
Studies involving **specimen and field samples:** State if relevant **permits** obtained, provide details of authority approving study; if none were required, explain why.	Not Applicable	

Dual Use Research of Concern (DURC)	Information included in the manuscript?	In which section is the information available? (Reagents and Tools Table, Materials and Methods, Figures, Data Availability Section)
Could your study fall under dual use research restrictions? Please check biosecurity documents and list of **select agents and toxins** (CDC): https://www.selectagents.gov/sat/list.htm	Not Applicable	
If you used a select agent, is the security level of the lab appropriate and reported in the manuscript?	Not Applicable	
If a study is subject to dual use research of concern regulations, is the name of the **authority granting approval and reference number** for the regulatory approval provided in the manuscript?	Not Applicable	

Reporting

The MDAR framework recommends adoption of discipline-specific guidelines, established and endorsed through community initiatives. Journals have their own policy about requiring specific guidelines and recommendations to complement MDAR.

Adherence to community standards	Information included in the manuscript?	In which section is the information available? (Reagents and Tools Table, Materials and Methods, Figures, Data Availability Section)
State if relevant guidelines or checklists (e.g., **ICMJE, MIBBI, ARRIVE, PRISMA**) have been followed or provided.	Not Applicable	
For **tumor marker prognostic studies,** we recommend that you follow the **REMARK** reporting guidelines (see link list at top right). See author guidelines, under 'Reporting Guidelines'. Please confirm you have followed these guidelines.	Not Applicable	
For **phase II and III randomized controlled trials**, please refer to the **CONSORT** flow diagram (see link list at top right) and submit the CONSORT checklist (see link list at top right) with your submission. See author guidelines, under 'Reporting Guidelines'. Please confirm you have submitted this list.	Not Applicable	

Data Availability

Data availability	Information included in the manuscript?	In which section is the information available? (Reagents and Tools Table, Materials and Methods, Figures, Data Availability Section)
Have **primary datasets** been deposited according to the journal's guidelines (see 'Data Deposition' section) and the respective accession numbers provided in the Data Availability Section?	Yes	data availability section in the maintext
Were **human clinical and genomic datasets** deposited in a public access-controlled repository in accordance to ethical obligations to the patients and to the applicable consent agreement?	Not Applicable	
Are **computational models** that are central and integral to a study available without restrictions in a machine-readable form? Were the relevant accession numbers or links provided?	Not Applicable	
If publicly available data were reused, provide the respective **data citations in the reference list**.	Yes	Reference includes these citations
