# Supplementary material for: PIWI proximity proteome reveals Set1-mediated piRNA biogenesis for transposon silencing at telomeres
Source: EMBO Rep. 2026 Aug 26;27(19):5490–529. doi: 10.1038/s44319-026-00901-6 (PMC13650161; doi:10.1038/s44319-026-00901-6)
Supplement: Supplementary file 3 [file 44319_2026_901_MOESM1_ESM.docx]

| **Proximity category** | ***gene*** | **Known interaction with PIWI** | **Localization** | **Germline/soma** | **Function** | **References** |
| --- | --- | --- | --- | --- | --- | --- |
| Piwi+Aub+Ago3 | *mino* |  | mitochondria | Germline/soma |  | ^1^ |
| Piwi+Aub+Ago3 | *tej* | Piwi, Aub, Ago3 | nuage | Germline | Precursor processing | ^2,3^ |
| Piwi+Aub | *papi* | Piwi, Ago3 | mitochondria | Germline/soma | Primary piRNA biogenesis | ^4–6^ |
| Piwi+Aub | *zuc* | Piwi, Aub, Ago3 | mitochondria | Germline/soma | Primary piRNA biogenesis | ^7–9^ |
| Aub+Ago3 | *kots* | Aub, Ago3 | nuage | Germline |  | ^10^ |
| Aub+Ago3 | *krimp* | Aub, Ago3 | nuage | Germline/soma | Ping-pong cycle | ^11–13^ |
| Aub+Ago3 | *tud* | Aub, Ago3 | nuage, pole granule | Germline | Ping-pong cycle | ^14,15^ |
| Piwi | *rtf1* |  | nucleus |  | Transcriptional silencing | ^16^ |
| Piwi | *nup154* |  | nucleus |  |  | ^17^ |
| Aub | *boYb* |  | nuage | Germline | Primary piRNA biogenesis | ^18^ |
| Aub | *mael* | Piwi | nucleus, nuage | Germline/soma | Transcriptional silencing | ^19–21^ |
| Aub | *qin* | Aub | nuage | Germline | Ping-pong cycle | ^22,23^ |
| Aub | *squ* | Aub, Piwi | nuage | Germline/soma |  | ^7,8^ |
| Aub | *vls* | Aub | nuage | Germline | Methyltransferase | ^13^ |
| Aub | *tapas* | Aub, Ago3 | nuage | Germline |  | ^24^ |
| Ago3 | *boot* |  | nucleus, nuage | Germline | Precursor transport | ^25,26^ |
| Ago3 | *nxf3* |  | nucleus, nuage | Germline | Precursor transport | ^25,26^ |

**Table EV1. Information of known piRNA pathway components identified by PIWI proximity labelling**

[1]	V. V. Vagin, Y. Yu, A. Jankowska, Y. Luo, K.A. Wasik, C.D. Malone, E. Harrison, A. Rosebrock, B.T. Wakimoto, D. Fagegaltier, F. Muerdter, G.J. Hannon, Minotaur is critical for primary piRNA biogenesis, RNA 19 (2013) 1064–1077. https://doi.org/10.1261/rna.039669.113.

[2]	V.S. Patil, T. Kai, Repression of Retroelements in Drosophila Germline via piRNA Pathway by the Tudor Domain Protein Tejas, Current Biology 20 (2010) 724–730. https://doi.org/10.1016/j.cub.2010.02.046.

[3]	Y. Lin, R. Suyama, S. Kawaguchi, T. Iki, T. Kai, Tejas functions as a core component in nuage assembly and precursor processing in *Drosophila* piRNA biogenesis, Journal of Cell Biology 222 (2023). https://doi.org/10.1083/jcb.202303125.

[4]	Y. Zhang, W. Liu, R. Li, J. Gu, P. Wu, C. Peng, J. Ma, L. Wu, Y. Yu, Y. Huang, Structural insights into the sequence-specific recognition of Piwi by *Drosophila* Papi, Proceedings of the National Academy of Sciences 115 (2018) 3374–3379. https://doi.org/10.1073/pnas.1717116115.

[5]	R. Hayashi, J. Schnabl, D. Handler, F. Mohn, S.L. Ameres, J. Brennecke, Genetic and mechanistic diversity of piRNA 3′-end formation, Nature 539 (2016) 588–592. https://doi.org/10.1038/nature20162.

[6]	L. Liu, H. Qi, J. Wang, H. Lin, PAPI, a novel TUDOR-domain protein, complexes with AGO3, ME31B and TRAL in the nuage to silence transposition, Development 138 (2011) 1863–1873. https://doi.org/10.1242/dev.059287.

[7]	A.D. Haase, S. Fenoglio, F. Muerdter, P.M. Guzzardo, B. Czech, D.J. Pappin, C. Chen, A. Gordon, G.J. Hannon, Probing the initiation and effector phases of the somatic piRNA pathway in *Drosophila*, Genes Dev 24 (2010) 2499–2504. https://doi.org/10.1101/gad.1968110.

[8]	A. Pane, K. Wehr, T. Schüpbach, zucchini and squash Encode Two Putative Nucleases Required for rasiRNA Production in the Drosophila Germline, Dev Cell 12 (2007) 851–862. https://doi.org/10.1016/j.devcel.2007.03.022.

[9]	H. Huang, Y. Li, K.E. Szulwach, G. Zhang, P. Jin, D. Chen, AGO3 Slicer activity regulates mitochondria–nuage localization of Armitage and piRNA amplification, Journal of Cell Biology 206 (2014) 217–230. https://doi.org/10.1083/jcb.201401002.

[10]	L.-X. Lim, W. Isshiki, T. Iki, S. Kawaguchi, T. Kai, The Tudor Domain-Containing Protein, Kotsubu (CG9925), Localizes to the Nuage and Functions in piRNA Biogenesis in D. melanogaster, Front Mol Biosci 9 (2022) 1–15. https://doi.org/10.3389/fmolb.2022.818302.

[11]	K. Sato, Y.W. Iwasaki, A. Shibuya, P. Carninci, Y. Tsuchizawa, H. Ishizu, M.C. Siomi, H. Siomi, Krimper Enforces an Antisense Bias on piRNA Pools by Binding AGO3 in the Drosophila Germline, Mol Cell 59 (2015) 553–563. https://doi.org/10.1016/j.molcel.2015.06.024.

[12]	A. Webster, S. Li, J.K. Hur, M. Wachsmuth, J.S. Bois, E.M. Perkins, D.J. Patel, A.A. Aravin, Aub and Ago3 Are Recruited to Nuage through Two Mechanisms to Form a Ping-Pong Complex Assembled by Krimper, Mol Cell 59 (2015) 564–575. https://doi.org/10.1016/j.molcel.2015.07.017.

[13]	X. Huang, H. Hu, A. Webster, F. Zou, J. Du, D.J. Patel, R. Sachidanandam, K.F. Toth, A.A. Aravin, S. Li, Binding of guide piRNA triggers methylation of the unstructured N-terminal region of Aub leading to assembly of the piRNA amplification complex, Nat Commun 12 (2021) 4061. https://doi.org/10.1038/s41467-021-24351-x.

[14]	K.M. Nishida, T.N. Okada, T. Kawamura, T. Mituyama, Y. Kawamura, S. Inagaki, H. Huang, D. Chen, T. Kodama, H. Siomi, M.C. Siomi, Functional involvement of Tudor and dPRMT5 in the piRNA processing pathway in Drosophila germlines, EMBO J 28 (2009) 3820–3831. https://doi.org/10.1038/emboj.2009.365.

[15]	Y. Kirino, A. Vourekas, N. Sayed, F. de Lima Alves, T. Thomson, P. Lasko, J. Rappsilber, T.A. Jongens, Z. Mourelatos, Arginine methylation of Aubergine mediates Tudor binding and germ plasm localization, RNA 16 (2010) 70–78. https://doi.org/10.1261/rna.1869710.

[16]	J.P. Clark, R. Rahman, N. Yang, L.H. Yang, N.C. Lau, Drosophila PAF1 Modulates PIWI/piRNA Silencing Capacity, Current Biology 27 (2017) 2718-2726.e4. https://doi.org/10.1016/j.cub.2017.07.052.

[17]	F. Muerdter, P.M. Guzzardo, J. Gillis, Y. Luo, Y. Yu, C. Chen, R. Fekete, G.J. Hannon, A Genome-wide RNAi Screen Draws a Genetic Framework for Transposon Control and Primary piRNA Biogenesis in Drosophila, Mol Cell 50 (2013) 736–748. https://doi.org/10.1016/j.molcel.2013.04.006.

[18]	D. Handler, D. Olivieri, M. Novatchkova, F.S. Gruber, K. Meixner, K. Mechtler, A. Stark, R. Sachidanandam, J. Brennecke, A systematic analysis of *Drosophila* TUDOR domain-containing proteins identifies Vreteno and the Tdrd12 family as essential primary piRNA pathway factors, EMBO J 30 (2011) 3977–3993. https://doi.org/10.1038/emboj.2011.308.

[19]	S.D. Findley, M. Tamanaha, N.J. Clegg, H. Ruohola-Baker, *Maelstrom* , a *Drosophila spindle* -class gene, encodes a protein that colocalizes with Vasa and RDE1/AGO1 homolog, Aubergine, in nuage, Development 130 (2003) 859–871. https://doi.org/10.1242/dev.00310.

[20]	R. Onishi, K. Sato, K. Murano, L. Negishi, H. Siomi, M.C. Siomi, Piwi suppresses transcription of Brahma-dependent transposons via Maelstrom in ovarian somatic cells, Sci Adv 6 (2020). https://doi.org/10.1126/sciadv.aaz7420.

[21]	G. Sienski, D. Dönertas, J. Brennecke, Transcriptional Silencing of Transposons by Piwi and Maelstrom and Its Impact on Chromatin State and Gene Expression, Cell 151 (2012) 964–980. https://doi.org/10.1016/j.cell.2012.10.040.

[22]	Z. Zhang, J. Xu, B.S. Koppetsch, J. Wang, C. Tipping, S. Ma, Z. Weng, W.E. Theurkauf, P.D. Zamore, Heterotypic piRNA Ping-Pong Requires Qin, a Protein with Both E3 Ligase and Tudor Domains, Mol Cell 44 (2011) 572–584. https://doi.org/10.1016/j.molcel.2011.10.011.

[23]	A. Anand, T. Kai, The tudor domain protein Kumo is required to assemble the nuage and to generate germline piRNAs in *Drosophila*, EMBO J 31 (2012) 870–882. https://doi.org/10.1038/emboj.2011.449.

[24]	V.S. Patil, A. Anand, A. Chakrabarti, T. Kai, The Tudor domain protein Tapas, a homolog of the vertebrate Tdrd7, functions in the piRNA pathway to regulate retrotransposons in germline of Drosophila melanogaster, BMC Biol 12 (2014) 61. https://doi.org/10.1186/s12915-014-0061-9.

[25]	M.F. ElMaghraby, P.R. Andersen, F. Pühringer, U. Hohmann, K. Meixner, T. Lendl, L. Tirian, J. Brennecke, A Heterochromatin-Specific RNA Export Pathway Facilitates piRNA Production, Cell 178 (2019) 964-979.e20. https://doi.org/10.1016/j.cell.2019.07.007.

[26]	E. Kneuss, M. Munafò, E.L. Eastwood, U.-S. Deumer, J.B. Preall, G.J. Hannon, B. Czech, Specialization of the *Drosophila* nuclear export family protein Nxf3 for piRNA precursor export, Genes Dev 33 (2019) 1208–1220. https://doi.org/10.1101/gad.328690.119.
