# Supplementary material for: PIWI proximity proteome reveals Set1-mediated piRNA biogenesis for transposon silencing at telomeres
Source: EMBO Rep. 2026 Aug 26;27(19):5490–529. doi: 10.1038/s44319-026-00901-6 (PMC13650161; doi:10.1038/s44319-026-00901-6)
Supplement: Supplementary file 6 [file 44319_2026_901_MOESM12_ESM.zip › Figure_5_Source_Data/Data Set 5D/H3K4me3_profile_genes_expressed_in_ovary.pdf]

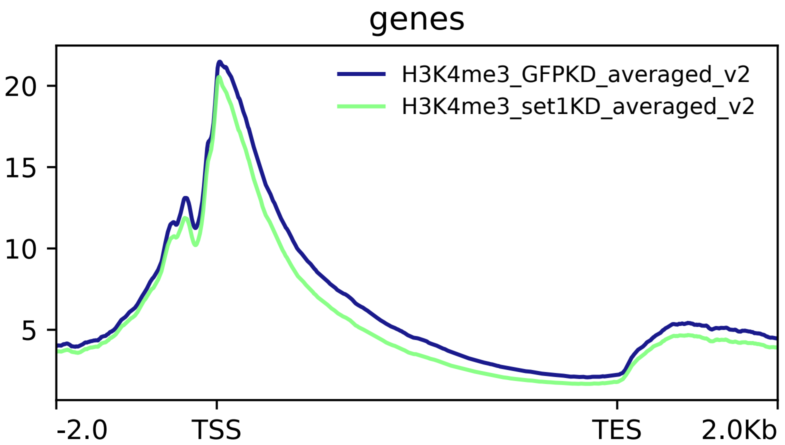
genes
H3K4me3_GFPKD_averaged_v2
H3K4me3_set1KD_averaged_v2
20
15
10
5
-2.0
TSS
TES
2.0Kb
