# Supplementary material for: PIWI proximity proteome reveals Set1-mediated piRNA biogenesis for transposon silencing at telomeres
Source: EMBO Rep. 2026 Aug 26;27(19):5490–529. doi: 10.1038/s44319-026-00901-6 (PMC13650161; doi:10.1038/s44319-026-00901-6)
Supplement: Supplementary file 8 [file 44319_2026_901_MOESM14_ESM.zip › Figure_7_Source_Data/Data Set 7E/TE_heatmap_gt3kb_enrichment_log2.pdf]

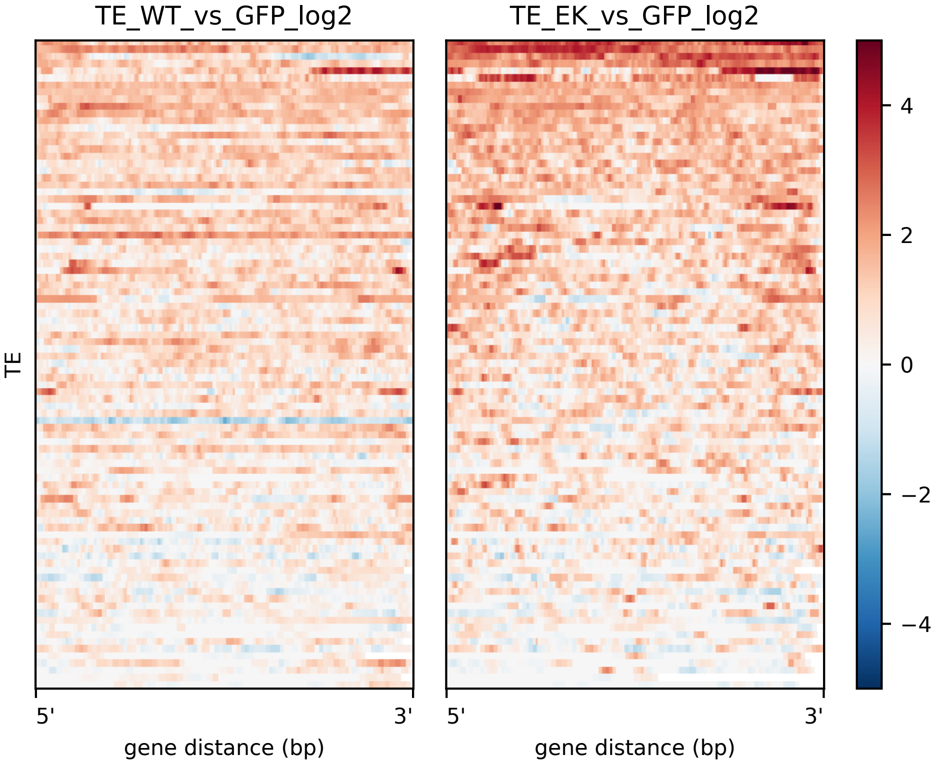

TE_WT_vs_GFP_log2
TE_EK_vs_GFP_log2
TE
5'
3'
5'
3'
gene distance (bp)
gene distance (bp)
4
2
0
−2
−4
