# Supplementary material for: PIWI proximity proteome reveals Set1-mediated piRNA biogenesis for transposon silencing at telomeres
Source: EMBO Rep. 2026 Aug 26;27(19):5490–529. doi: 10.1038/s44319-026-00901-6 (PMC13650161; doi:10.1038/s44319-026-00901-6)
Supplement: Supplementary file 8 [file 44319_2026_901_MOESM14_ESM.zip › Figure_7_Source_Data/Data Set 7C/GFPset1_heatmap_genes_expressed_in_ovary_zmax_enrichment_log2.pdf]

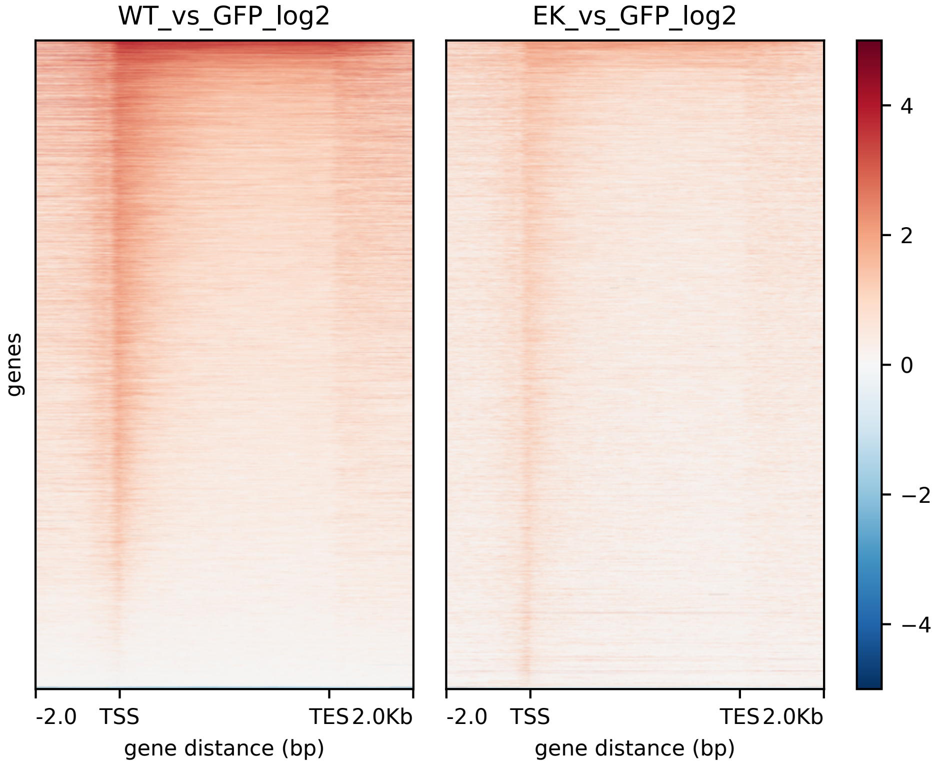
WT_vs_GFP_log2
EK_vs_GFP_log2
genes
-2.0
TSS
TES
2.0Kb
gene distance (bp)
-2.0
TSS
TES
2.0Kb
gene distance (bp)
4
2
0
−2
−4
